# Combining a diffusion model with a sparse coding strategy to improve the machine translation accuracy of culturally loaded words in English news texts

**DOI:** 10.1371/journal.pone.0356146

**Published:** 2026-08-28

**Authors:** Qin Yu, Tao Mai

**Affiliations:** 1 School of Applied Foreign Languages, Guangdong Polytechnic of Industry and Commerce, Guangzhou, Guangdong Province, China; 2 Department of Applied Linguistics, College of Chinese Language and Culture, Jinan University, Guangzhou, Guangdong Province, China; Philadelphia University, JORDAN

## Abstract

News texts are characterized by their timeliness, contextual dependence, and density of proper nouns. Cultural terms, often appearing as metaphors, allusions, or institutional terms, are frequently long-tailed words lacking sufficient corpus support, easily leading to semantic drift and cultural information loss in translation. To address these issues, this paper combines a diffusion model with a sparse coding strategy to improve the accuracy and cultural adaptability of cultural terms in English news translation. In the encoding stage, a sparse semantic extraction module based on L1 norm constraints and dictionary learning is constructed to sparsely represent the input sequence, strengthening the expression of key information about cultural terms. In the decoding stage, a diffusion generation mechanism is introduced to gradually reconstruct the target text through multi-step denoising. Simultaneously, by combining time-step-aware gating and cultural ontology knowledge constraints, end-to-end collaborative optimization of cultural feature extraction and target language generation is achieved. Experimental results show that compared with mainstream models such as Transformer, mBART-50 (many to many multilingual machine translation), and DiffuSeq (Sequence to sequence text generation with diffusion models), proposed model achieves BLEU (Bilingual Evaluation Understudy)-4 and METEOR (Metric for Evaluation of Translation with Explicit Ordering) scores of 27.8% and 29.2%, respectively, on long-tail news text (sentences of 46 words or more). In the unregistered vocabulary scenario, the accuracy and recall rates were 73.7% and 65.2%, respectively, and the cultural semantic consistency index reached 0.892. The results indicate that the proposed synergistic mechanism of sparse coding and diffusion generation facilitates weak semantic recognition and progressive semantic restoration, providing a technical path for cross-cultural machine translation in international news communication that combines accuracy with cultural adaptability.

## Introduction

Against the backdrop of accelerating global informatization, words and phrases embodying specific social, historical, and value concepts frequently appear in English news texts. Correctly translating these words is crucial for cross-cultural communication. Current Neural Machine Translation (NMT) models often suffer from semantic drift and loss of cultural connotation when processing such words [1,2]. This is particularly true when dealing with long-tail feature sets and unregistered culturally specific words, making accurate translation and adaptation difficult due to a lack of training data, inadequate context modeling, and sparse semantic representation [3,4]. Traditional translation methods based on bilingual dictionaries and rule-based post-editing have poor generalization capabilities. While pre-trained language models can improve fluency, they tend to overlook the deeper semantics underlying low-frequency cultural concepts. Improving the model’s ability to recognize culturally specific terms while ensuring their semantic integrity is a core issue currently under investigation in machine translation research.

Existing research mainly focuses on the identification and processing of culturally loaded words in English news texts in machine translation. These words often have strong cultural attributes and semantic complexity, and are prone to mistranslation or omission due to the scarcity of the corpus and insufficient context modeling [5–7]. To address the translation barriers caused by out-of-vocabulary (OOV) words, Duan Jiayan proposed an OOV processing method based on semantic similarity replacement [8]. Budaya I. Gede Bintang Arya improved the performance of NMT by combining the word vectorization strategy of Word2Vec (word to vector) in a low-resource environment, verifying the role of word embedding in promoting translation quality in knowledge-scarce scenarios [9]. Wang Jinghan combined a refined attention mechanism with a neural network to improve the semantic inaccuracy caused by insufficient contextual relationship mapping [10]. At the same time, Baniata Laith H strengthened the model’s ability to deal with unknown words by using subword units and shared vocabulary strategies [11]. Jiao Jiao proposed a cross-language dynamic masking mechanism to explore semantic alignment and cultural adaptation issues, which has positive significance for non-literal translation scenarios [12]. Overall, existing research mainly uses word vectorization, subword modeling, and attention mechanisms to improve translation accuracy. Although these methods have alleviated the scarcity and contextual bias of culturally loaded words to a certain extent, they generally lack deep semantic recovery and cultural adaptation mechanisms for long-tail and unregistered cultural words. In response to the shortcomings of traditional methods in translating long-tail and unregistered culturally loaded words, researchers have begun to try to introduce diffusion models and sparse coding strategies to enhance the semantic recovery ability and cultural sensitivity of the model [13,14]. Su Zengye systematically reviewed the development of diffusion models in natural language processing, pointing out that they have the advantages of fine-grained controllability and parallel generation in text generation and editing tasks, providing new ideas for complex semantic recovery [15]. Lovelace Justin combined the diffusion model with a pre-trained language model to construct a language autoencoder, achieving semantic generation in the latent space and effectively overcoming the limitations of discrete modeling [16]. Gulrajani Ishaan promoted a breakthrough in the performance of large-scale diffusion language models in text generation by optimizing maximum likelihood training and computing resource allocation [17]. Zhu Shaolin reviewed sparse models in large-scale multilingual NMT, showing that sparse modeling can improve parameter efficiency, suppress interference, and improve cultural adaptation translation effects [18]. Sparse coding can provide a semantic anchor for diffusion models, addressing the issue of weak initial signals. Diffusion processes utilize multi-step denoising to achieve progressive reconstruction from fuzzy representation to precise translation, constrained by cultural knowledge. However, both approaches are still largely explored independently, and research is lacking on combining their strengths to address the semantic drift of culturally loaded words or the loss of cultural information in English news texts. Consequently, cultural adaptability and translation accuracy remain to be improved.

To address these issues, this paper combines sparse coding with a diffusion generation mechanism for translating culturally loaded words in English news text. By constructing a sparse semantic space during the encoding phase to amplify cultural signals, and introducing a conditional diffusion mechanism during the decoding phase to achieve multi-step denoising of cultural perception, this approach also incorporates cultural ontology knowledge for trajectory calibration, ultimately improving the model’s accuracy in identifying cultural concepts, selecting strategies, and generating them. This paper achieves dual improvements in semantic fidelity and cultural adaptability through sparse coding and diffuse generation mechanisms. This approach can provide a reference for automated translation systems in mainstream international media and contribute to accurate cross-cultural understanding in global news dissemination.

This paper addresses the semantic drift and lack of cultural information in the translation of culturally loaded words in news English by proposing a collaborative optimization mechanism of sparse coding and diffusion generation. The main contributions of this paper include:

(1) Conditional coupling of sparse semantic anchors and diffusion trajectories: Unlike previous methods that only used sparse coding for feature compression or independent preprocessing, this paper explicitly maps the truncation coefficients obtained from sparse coding to a cultural condition matrix and injects it into each step of the diffusion decoder’s denoising process in a cross-attention manner, enabling the sparse cultural signal to continuously guide multi-step generation.
(2) Time-step-aware dynamic gating fusion: Existing diffusion models typically use fixed conditional fusion weights in text generation. This paper proposes a learnable time-step-aware gating mechanism that automatically adjusts the intervention intensity of cultural conditions based on the current denoising progress, achieving flexible control of cultural information in translation tasks.
(3) Cultural knowledge alignment with augmented Lagrange soft constraints: This paper embeds bilingual cultural ontology knowledge into the training loss in the form of augmented Lagrange multipliers, dynamically maintaining the consistency between the generation trajectory and the knowledge graph during back-diffusion, reducing the risk of cultural mistranslation while maintaining generation flexibility.

## Construction of a translation mechanism for culturally loaded words in news English

**Fig 1:**
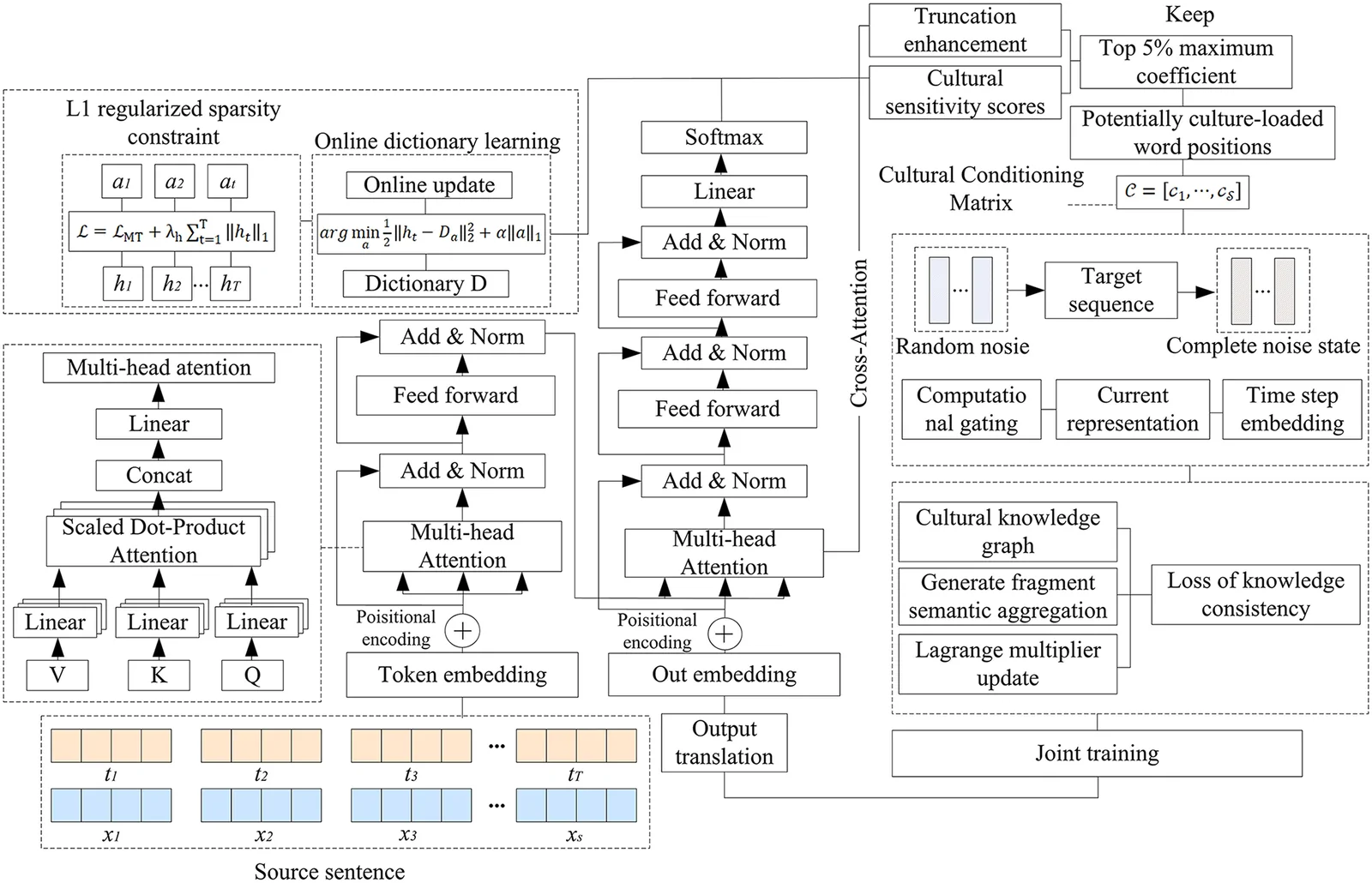
Culturally loaded word translation framework.

The framework for translating culturally loaded words in news English constructed in this paper is based on the Transformer and integrates sparse coding, diffuse generation, and cultural knowledge guidance mechanisms. The framework is shown in Fig 1.

In this framework, an English news sentence is input, and the Transformer encoder generates hidden states. The sparse coding module compresses the hidden state of each word into sparse coefficients with only a few non-zero components through L1 regularization and dictionary learning. The truncated sparse coefficients are linearly projected into a low-dimensional dense vector to form the cultural condition matrix. The decoder starts with a completely random noise sequence. In 1000 steps of reverse denoising, each step simultaneously considers three aspects: 1) the current noise sequence itself; 2) the source language content of the encoder; and 3) the cultural condition matrix. The gating mechanism dynamically adjusts the weight of the cultural conditions: in the early stages of denoising, the gating value is small, and the model mainly recovers the overall skeleton of the sentence; in the later stages of denoising, the gating value increases, and the model relies more on the cultural conditions to accurately select the translation of culturally loaded words. After each denoising step, the model compares the currently generated segment with the cultural knowledge graph. If the deviation is too large, a soft penalty is applied through the loss function to gradually pull the generated trajectory towards cultural consistency. In the inference stage, the output that best fits the cultural knowledge is selected from multiple candidate translations through reordering.

To avoid symbol ambiguity and ensure model reproducibility, this paper uses standardized notation, and the symbol explanations are shown in Table 1:

**Table 1:** Explanation of formula symbols.

Symbol	Meaning	Dimension
$h_{t}$	The encoder’s hidden state at time t	$R^{d},d=\text{5}\text{1}\text{2}$
D	Overcomplete dictionary matrix	$R^{d\times K},K=\text{1}\text{0}\text{2}\text{4}$
$a_{t}$	Sparse coefficient vector	$R^{K}$
${\tilde{a}}_{t}$	Truncation of sparse coefficients	$R^{K}$ *(Only the top 5% are non-zero)*
C	Culture condition matrix	$R^{d_{c}\times S},d_{c}=\text{30}$
$x_{u}$	Noise sequence at diffusion step u	$R^{N\times V}$ (Discrete distribution)
$g_{u}$	Time-step perceptual gating scalar	$g_{u}\in \left[\text{0},\text{1}\right]$
$L_{MT}$	Translation cross-entropy loss	Scalar
$mat\text{h}calL_{diff}$	Diffusion variational lower bound loss	Scalar
$L_{kc}$	Knowledge consistency loss	Scalar

### Cultural semantic feature extraction based on sparse regularization

For the machine translation of English news text, this paper adopts the Transformer encoder-decoder architecture as the basic framework. After the input text is tokenized and embedded, the encoder uses a multi-layer self-attention mechanism to generate a hidden state sequence ${\left\{h_{t}\right\}}_{t=\text{1}}^{T}$, which contains a comprehensive semantic representation of the source language in the context of the text. Culturally loaded words have the characteristics of low frequency distribution and cultural semantic scarcity, and relevant information is easily weakened and submerged in the hidden state [19]. This paper introduces a sparse regularization module in the encoder output layer, combined with the online dictionary learning method, to compress the high-dimensional semantic vector output by the encoder into a sparse space spanned by a small number of basis vectors, and extract the significant features corresponding to the culturally loaded words to improve their distinguishability.

Assume that the hidden state of the encoder at time t is $h_{t}\in R^{d}$. To avoid uniform activation in all dimensions, this paper introduces the L1 regularization constraint in the translation loss function, as shown in Formula (1):


$$\begin{array}{c} L=L_{MT}+\lambda_{h}\sum_{t=\text{1}}^{T}\|h_{t}{\|}_{\text{1}} \end{array}$$ (1)

$L_{MT}$ is the cross-entropy loss of the translation model, and $\lambda_{h}>\text{0}$ is the sparsification strength. L1 regularization forces the hidden state to be sparsely distributed across dimensions, retaining only a small number of components with large responses. This process amplifies the relevant semantic activations of culturally loaded words in the early encoding stage, weakening background and irrelevant features. Based on the initial sparsification, the hidden state is further mapped to the sparse semantic space spanned by the overcomplete dictionary through sparse coding. Given the encoder hidden state $h_{t}\in R^{d}$, the dictionary $D\in R^{d\times k}$ (K≫d), and the sparse coefficients $a\in R^{K}$, as shown in Formula (2):


$$\begin{array}{c} a_{t}=arg\underset{a}{min}\frac{\text{1}}{\text{2}}\|h_{t}-D_{a}{\|}_{\text{2}}^{\text{2}}+\alpha \|a{\|}_{\text{1}} \end{array}$$ (2)

The first term ensures the accuracy of the reconstruction of the hidden state by the sparse coefficients, while the second term controls sparsity through the L1 norm, withα>0 being the sparsity adjustment parameter. Thus, $h_{t}$ is re-represented as a combination of a few basis vectors, with the basis vectors corresponding to non-zero positions representing the underlying cultural semantic features. To cope with the continuous emergence of long-tail words and unregistered words in news corpus, the dictionary D is dynamically updated using an online dictionary learning algorithm. In each small batch of data, the dictionary D is first fixed and the sparse coefficient $\left\{a_{t}\right\}$ is solved using coordinate descent; then the coefficient is fixed and the dictionary is updated [20,21]:


$$\begin{array}{c} D\leftarrow P(D-l\nabla_{D}\sum_{t}\frac{\text{1}}{\text{2}}{\left\|h_{t}-D_{a_{t}}\right\|}_{\text{2}}^{\text{2}}) \end{array}$$ (3)

𝓁 is the learning rate, and P represents the projection operation of the normalized column vector. Through online updates, the dictionary continuously absorbs new cultural semantic basis vectors, improving the model’s coverage of low-frequency culturally loaded words. In order to further highlight the weight of culturally loaded words in sparse representation, a truncation retention strategy is designed. The components of $a_{t}$ are sorted by absolute value, and only the top 5% of the largest non-zero items are retained, and the rest are set to zero, to obtain the truncated coefficient ${\tilde{a}}_{t}$[22]:


$$\begin{array}{c} {\tilde{a}}_{t}=T_{m}(a_{t}),m=[\text{0}.\text{0}\text{5}K] \end{array}$$ (4)

During this process, low-amplitude but frequently occurring noise features are eliminated, retaining only sparse activations with high confidence, making the semantic patterns corresponding to culturally loaded words more prominent in the representation. Based on the truncated sparsity coefficient, a cultural sensitivity scoring function $S_{t}$ is constructed to automatically locate potential culturally loaded words. The score consists of two components: energy concentration ${EC}_{t}$ and cross-sentence stability ${ST}_{t}$. Energy concentration measures whether sparse activations are concentrated in a small number of dimensions, as shown in Formula (5):


$$\begin{array}{c} {EC}_{t}=\frac{{\left\|{\tilde{a}}_{t}\right\|}_{\text{2}}^{\text{2}}}{{\left\|a_{t}\right\|}_{\text{2}}^{\text{2}}} \end{array}$$ (5)

Cross-sentence stability reflects the stability of cultural semantic signals at the cross-sentence level, as shown in Formula (6):


$$\begin{array}{c} {ST}_{t}=\frac{\text{1}}{C^{\text{2}}}\sum_{i,j}\frac{\left\langle {\tilde{a}}_{t}^{\left(i\right)},{\tilde{a}}_{t}^{\left(j\right)}\right\rangle }{{\left\|{\tilde{a}}_{t}^{\left(i\right)}\right\|}_{\text{2}}{\left\|{\tilde{a}}_{t}^{\left(j\right)}\right\|}_{\text{2}}} \end{array}$$ (6)

$\left\{{\tilde{a}}_{t}^{\left(i\right)}\right\}$ represents the sparse coefficient set of the same word form in different contexts, and C is the number of occurrences. The final score is defined as the weighted sum, as shown in Formula (7):


$$\begin{array}{c} S_{t}=\gamma \cdot {EC}_{t}+(\text{1}-\gamma )\cdot {ST}_{t} \end{array}$$ (7)

γ∈[0,1] is a weight parameter. If $S_{t}$ is higher than the adaptive threshold τ, the word is marked as potentially culturally loaded.

### Multi-step semantic denoising translation generation based on the diffusion model

In the machine translation of English news text, traditional autoregressive decoders are limited by a unidirectional generation path and a local decision-making mechanism, making it difficult to achieve a coordinated optimization of semantic integrity and contextual consistency when dealing with long-tail culturally loaded words. In particular, when cultural information at the source is sparse or implicit, the decoding process is prone to semantic drift or cultural mistranslation [23,24]. To this end, this paper constructs a non-autoregressive decoding architecture based on a discrete diffusion process and builds a multi-step semantic denoising translation generation mechanism driven by a diffusion model. The generation of the target language sequence is modeled as an inverse denoising process that gradually recovers from a completely noisy state to a semantically complete translation. By utilizing the progressive optimization capability in the time dimension, the sparse cultural semantic features extracted are dynamically integrated in each denoising step to achieve continuous tracking and refined reconstruction of deep cultural information.

Let the source sequence length be S, the source sequence encoder output be ${\left\{h_{\text{𝓈}}\right\}}_{\text{𝓈}=\text{1}}^{S}$, and the corresponding truncated sparse coefficient be ${\left\{{\tilde{a}}_{\text{𝓈}}\right\}}_{\text{𝓈}=\text{1}}^{S}$. Let the diffusion time step beu∈{0,1,⋯,T} (assuming T=1000). The target sequence length is N, and the vocabulary size is V. For the forward noise addition process, let the true target sequence be $y=\left(y_{\text{1}},\cdots ,y_{N}\right)$, and the forward process be defined as a Markov chain [25,26]:


$$\begin{array}{c} q(x_{u}|x_{u-\text{1}})=\prod_{n=\text{1}}^{N}q(x_{u}^{\left(n\right)}|x_{u-\text{1}}^{\left(n\right)}) \end{array}$$ (8)

For single-position transitions, a parameterized transfer matrix $M_{u}\in R^{V\times V}$ is used. To achieve gradual destruction of semantics and facilitate the analysis of the posteriori, this paper adopts a gradual scrambling-preserving hybrid transfer:


$$\begin{array}{c} q(x_{u}^{\left(n\right)}=v|x_{u-\text{1}}^{\left(n\right)}=v^{\prime })=(\text{1}-\beta_{u}){\text{1}}_{v=v^{\prime }}+\beta_{u}\frac{\text{1}}{V} \end{array}$$ (9)

$\beta_{u}\in (\text{0},\text{1})$ is a noise schedule set with the step size, ensuring that the overall noise increases with increasing u, making $q(x_{T})$ approximately completely randomly distributed, meaning that the initial translation is completely corrupted. This forward process projects the true discrete sequence onto a resolvable distribution of the noise sequence, providing a criterion for reverse learning. For the forward noise-adding stage of the discrete diffusion process, this paper follows the standard discrete diffusion model definition in Formula (8) and Formula (9) and adopts a mask-uniform hybrid transfer matrix to make the initial target sequence gradually become a uniform random distribution.

The reverse process is given by the parameterized network $p_{\theta }(x_{u-\text{1}}|x_{u},H,C)$, where $H=\left\{h_{\text{𝓈}}\right\}$ is the encoder output and C is the cultural conditioning matrix projected from the sparse coefficients $\left\{{\tilde{a}}_{\text{𝓈}}\right\}$. Each truncated coefficient is mapped to a dense conditioning vector [27,28]:


$$\begin{array}{c} c_{s}=W_{c}{\tilde{a}}_{s}+b_{c},C=[c_{\text{1}},\cdots ,c_{S}]\in R^{d_{c}\times S} \end{array}$$ (10)

$W_{\text{𝒸}}\in R^{d_{\text{𝒸}}\times K}$ is the linear projection (K is the dictionary dimension), and $d_{\text{𝒸}}$ is the conditional vector dimension. The decoder adopts a multi-layer Transformer structure. In each layer, in addition to the conventional self-attention and cross-attention to the encoder, an additional cross-attention sub-layer for the cultural condition matrix C is added, and the output of this sub-layer is weighted fused through the time-step-aware gating unit. An update of the layer can be written as [29]:


$$\begin{array}{c} S^{\left(l\right)}={selfAtt}^{\left(l\right)}(Z_{u}^{\left(l-\text{1}\right)}) \end{array}$$ (11)

$$\begin{array}{c} A_{H}^{\left(l\right)}={CrossAtt}^{\left(l\right)}(S^{\left(l\right)},H) \end{array}$$ (12)

$$\begin{array}{c} A_{C}^{\left(l\right)}={CrossAtt}^{\left(l\right)}(S^{\left(l\right)},C) \end{array}$$ (13)

Assume that the gate scalar of time step u is $g_{u}\in [\text{0},\text{1}]$, then the output of l layer is as shown in Formula (14):


$$\begin{array}{c} Z_{u}^{\left(l\right)}={FFN}^{\left(l\right)}(S^{\left(l\right)}+A_{H}^{\left(l\right)}+g_{u}⨀A_{C}^{\left(l\right)}) \end{array}$$ (14)

⨀ means element-wise scaling. The output of the last layer L is linearly projected into position-level logits to give the predicted distribution of $x_{u-\text{1}}$, as shown in Formula (15) and Formula (16):


$$\begin{array}{c} {\text{𝓁}}_{u}^{\left(n\right)}=W_{o}z_{u}^{\left(L,n\right)}+b_{o} \end{array}$$ (15)

$$\begin{array}{c} p_{\theta }(x_{u-\text{1}}^{\left(n\right)}|x_{u},H,C)=softmax({\text{𝓁}}_{u}^{\left(n\right)}) \end{array}$$ (16)

To achieve a dynamic trade-off from coarse-grained to fine-grained, the gated scalar $g_{u}$ is determined by the temporal embedding and the current sequence state. First, the temporal embedding $\varsigma_{u}$ is constructed, and the current noisy representation is globally aggregated ${\overset{―}{z}}_{u}=\frac{\text{1}}{N}\sum_{n}z_{u}^{\left(\text{0},n\right)}$. The timestep embedding is $\varsigma_{u}\in R^{\text{64}}$, and the current sequence is globally pooled as $\overset{―}{z}u=\frac{\text{1}}{N}\sum nz_{u}^{\left(\text{0},n\right)}\in R^{d},(d=\text{512})$. Learnable parameters are: $W_{\varsigma }\in R^{d_{g}\times \text{6}\text{4}},W_{z}\in R^{d_{g}\times d},\omega_{g}\in R^{d_{g}},b_{g}\in R^{d_{g}}$, where the gated projection dimension $b_{g}=\text{1}\text{2}\text{8}$. The gated scalar is defined as shown in Formula (17):


$$\begin{array}{c} g_{u}=\sigma \left(\omega_{g}^{⊺}tan\text{h}(W_{\varsigma }\varsigma_{u}+W_{z}{\overset{―}{z}}_{u}+b_{g})\right) \end{array}$$ (17)

$W_{\varsigma },W_{z},\omega_{g},b_{g}$ are learnable parameters, and σ is a sigmoid. This definition enables $g_{u}$ to respond simultaneously to both the current diffusion progress and the semantic integrity of the current sequence: In the early stages of the backward pass (when u is close to T and noise is high), $\varsigma_{u}$ results in a smaller $g_{u}$, thereby reducing the direct influence of cultural conditioning while allowing the model to recover the semantic skeleton based on the encoder H. In the late stages of the backward pass (when u is close to 0 and noise is low), $g_{u}$ increases, amplifying the influence of cultural conditioning on final lexical selection and facilitating accurate restoration of culturally loaded word translation.

The $p_{\theta }(x_{u-\text{1}}|x_{u},H,C)$ predicted by the network at each step is regarded as the residual correction distribution of the posterior of the forward noise process. During training, the true posterior is fitted by minimizing the variational lower bound, as shown in Formula (18):


$$\begin{array}{c} L_{diff}=\sum_{u=\text{1}}^{T}E_{y,x_{u}\sim q(\cdot |y)}[-logp_{\theta }(x_{u-\text{1}}|x_{u},H,C)] \end{array}$$ (18)

The inference phase samples from $x_{T}\sim Uniform$ iterations in reverse order, as shown in Formula (19):


$$\begin{array}{c} x_{u-\text{1}}\sim p_{\theta }(x_{u-\text{1}}|x_{u},H,C),u=T,\cdots ,\text{1} \end{array}$$ (19)

**Fig 2:**
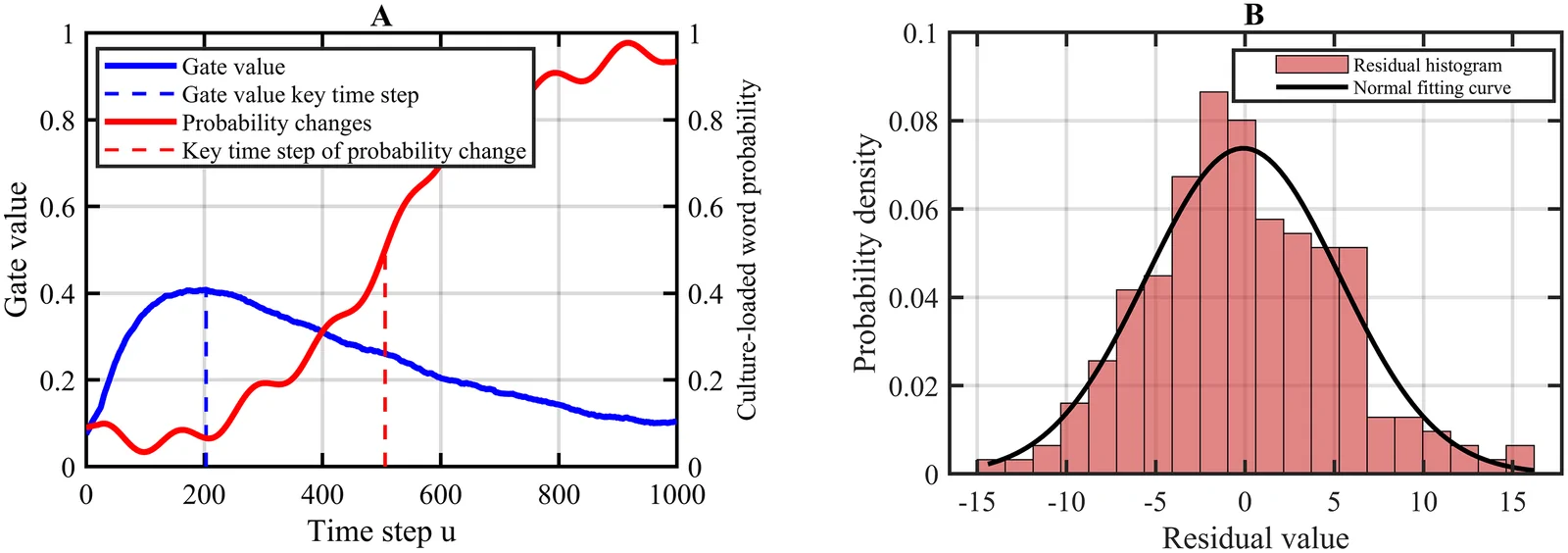
Gating dynamics and residual distribution analysis of culturally loaded word translation in the diffusion model. Panel A shows the time-step-aware gating value and the change in the probability of culturally loaded words. Panel B shows the predicted residual distribution.

At each step, prediction and residual correction are applied to gradually guide the noise distribution back to a semantically structured translation distribution. When the sequence approaches u≈0, the amplification of gated $g_{u}$ ensures that the influence of cultural features at the lexical level is strengthened, thereby improving the lexical accuracy of culturally loaded words. Fig 2 shows the time-step perceptual gate value, the change in the probability of culturally loaded words, and the predicted residual distribution:

Fig 2 shows the relationship between the time-step-aware gating value, the probability of culturally loaded words, and the predicted residual distribution during the reverse denoising process. In Fig 2A, based on the fusion of the diffusion model and sparse coding, the time-step-aware gating value shows a trend of first increasing and then decreasing. In Fig 2B, the histogram shows the distribution of residuals between the model’s predicted translation and the true translation. Most of the residuals are concentrated in a small range, indicating that the model performs stably in overall semantic recovery. The normal fitting curve closely matches the histogram, indicating that the residual distribution is approximately normal. The model reduces cultural information loss and lexical drift by restoring the semantic skeleton at the coarse-grained stage and adjusting culturally loaded words at the fine-grained stage.

### Generative trajectory constraints guided by cultural ontology knowledge

An external bilingual cultural ontology is introduced into the diffusion denoising generation process to construct a cultural equivalence relationship graph and encode it into a low-dimensional embedding with the same dimensionality as the encoder’s sparse condition. In each backward denoising step u, the knowledge embedding is semantically aligned with the decoder’s representation of the current generated segment and a knowledge consistency measure is calculated. This measure is used as a soft constraint in the form of Lagrange multipliers and embedded in the total loss. During the inference phase, a knowledge confidence-based reranking mechanism is used to screen candidate generation paths, thereby ensuring generation flexibility while enhancing the standardization of cultural equivalence and cross-cultural semantics.

This paper constructs a cultural knowledge graph G=(V,E), where nodes v∈V represent cultural entities, including bilingual terms, customary terms, and proper nouns. Edges $\left(v_{i},v_{j}\right)\in E$ represent semantic associations, and confidence weights are stored for each node. Table 2 summarizes its statistical information.

**Table 2:** Overview of cultural ontology knowledge graph.

Project	Specifications
Total number of cultural entity nodes	186
Total number of relation edges	312
Relationship type	Equivalence relation, hypernym relation, association relation, comparison relation
Embedding dimension	30
Main cultural categories	Religion, politics, festivals, social movements
Language coverage	English, Chinese

Note: Equivalence relation indicates a direct semantic correspondence in a cross-cultural context; hypernym relation indicates a genus-species relationship; association relation captures non-hierarchical cultural associations; comparison relation indicates a culturally specific distinction without a direct counterpart.

The cultural ontology knowledge graph focuses on four categories of concepts that are most challenging to translate in news English-to-Chinese translation scenarios, have high frequency of occurrence, and are most culturally semantically dense: religion, politics, festivals, and social movements. The selection of ontology nodes follows these principles: 1) Based on word frequency statistics and translation error rate analysis using the WMT18 and TED corpora, cultural terms that appear in the top 20% of news contexts and have a translation error rate higher than 30% in the existing baseline model Transformer are selected as candidate seed nodes; 2) Three bilingual experts with cross-cultural research backgrounds independently annotated and merged the nodes, and the final node set was reached after three rounds of discussion; 3) Semantic relationships between nodes are completed and verified through knowledge graph embedding to ensure the integrity and consistency of relationship edges. Based on the cross-cultural translation equivalence theory, experts determine the relationship type between each pair of nodes, including equivalence, superior, association, and comparison relationships. Each relationship must be confirmed by at least two experts before being included in the edge set. 4) The TransE model is used to map graph nodes to a low-dimensional real-valued vector space. Using head entity, relation, and tail entity triples as training units, the node embedding matrix is obtained after 100 rounds of iterative training. The margin parameter of TransE is set to 1.0, and the learning rate is 0.01. 5) 20% of the nodes and relation edges are randomly selected and blindly verified by two independent experts who did not participate in the construction. The node attribution accuracy rate is 94.7%, and the relation type accuracy rate is 91.2%, indicating that the ontology quality meets the requirements of downstream tasks.

By deduplicating all annotated cultural terms appearing in 500 test samples and calculating their hit rate in the ontology node set, the current 186 entities and 312 relationship edges cover 83.6% of the annotated culturally loaded terms in the test subset constructed in this study. The uncovered 16.4% mainly consists of highly long-tail proper nouns or emerging online cultural terms. The sparse coding module in this framework dynamically absorbs newly emerging cultural semantic basis vectors through an online dictionary learning mechanism, providing a certain degree of generalization compensation for cultural words not included in the ontology during the inference stage. Therefore, although the current ontology is compact in size, it has sufficient semantic representativeness within the scope of news texts evaluated in this study, and can support the validity requirements of cross-cultural translation quality assessment to a certain extent.

Applying the graph embedding function ϕ(·) to the graph yields a node embedding matrix$E=\left[e_{\text{1}},\cdots ,e_{\left|V\right|}\right]\in R^{d_{c}\times \left|V\right|}$, where the conditional vector embedding dimension $d_{c}$ is the same as the sparse conditional vector ${\text{𝒸}}_{\text{𝓈}}$, facilitating direct similarity calculations, as shown in Formula (20):


$$\begin{array}{c} e_{m}=\phi (v_{m}),e_{m}\in R^{d_{c}} \end{array}$$ (20)

For the set of locations that are determined to be potential cultural source locations, their conditional vectors are embedded with the graph nodes for nearest neighbor matching:


$$\begin{array}{c} {\text{𝓀}}_{\text{𝓈}}=arg\underset{m}{max}cos({\text{𝒸}}_{\text{𝓈}},e_{m}),{conf}_{\text{𝓈}}=cos({\text{𝒸}}_{\text{𝓈}},e_{{\text{𝓀}}_{\text{𝓈}}}) \end{array}$$ (21)

As shown in Formula (21), cos is the cosine similarity, and ${conf}_{\text{𝓈}}\in [\text{0},\text{1}]$ represents the matching confidence between the source position s and the graph node ${\text{𝓀}}_{\text{𝓈}}$. If ${conf}_{s}$ is less than the set lower limit, the position can be considered a “weak match” and participates in the constraint with a lower weight.

In the diffusion decoder step u, let $Z_{u}^{\left(l\right)}=\left[z_{u}^{\left(L,\text{1}\right)},\cdots ,z_{u}^{\left(L.N\right)}\right]$ be the token representation of the L layer. The semantic vector of the generator is focused on the source cultural position 𝓈 by using the cross-attention weight ${\text{𝒶}}_{u,n,\text{𝓈}}$ of the decoder to the encoder H. Let ${\text{𝒶}}_{u,n,\text{𝓈}}$ be the cross-attention weight of the target position n with respect to the source position 𝓈 in the L-th layer of the decoder (satisfying $\sum_{s}{\text{𝒶}}_{u,n,\text{𝓈}}=\text{1}$). Then the aggregate representation of the source cultural position s is: [30]:


$$\begin{array}{c} r_{u}^{\left(\text{𝓈}\right)}=\frac{\text{1}}{\sum_{n}{\text{𝒶}}_{u,n,\text{𝓈}}}\sum_{n=\text{1}}^{N}{\text{𝒶}}_{u,n,\text{𝓈}}z_{u}^{\left(L,n\right)}\in R^{d_{c}} \end{array}$$ (22)

The aggregated representation captures the semantic representation of the target sequence with respect to the source cultural position 𝓈 in the current denoised state. The cosine similarity between this representation and the knowledge embedding $e_{{\text{𝓀}}_{\text{𝓈}}}$ is calculated as shown in Formula (23):


$$\begin{array}{c} {sim}_{u}^{\left(\text{𝓈}\right)}=cos(r_{u}^{\left(\text{𝓈}\right)},e_{{\text{𝓀}}_{\text{𝓈}}}) \end{array}$$ (23)

Assume that the similarity is expected to be above the threshold $\eta_{\text{𝓈}}$, that is, the constraint form is as shown in Formula (24):


$$\begin{array}{c} g_{u}^{\left(\text{𝓈}\right)}(\theta )=\eta_{\text{𝓈}}-{sim}_{u}^{\left(\text{𝓈}\right)}\leq \text{0} \end{array}$$ (24)

This inequality constraint is incorporated into the training objective, and the augmented Lagrangian method is used to construct the knowledge loss for each step:


$$\begin{array}{c} L_{kc}=\sum_{\text{𝓈}\in C}\left[{\text{𝑜}}_{u}^{\left(\text{𝓈}\right)}g_{u}^{\left(\text{𝓈}\right)}+\frac{\rho }{\text{2}}{\left(g_{u}^{\left(\text{𝓈}\right)}\right)}^{\text{2}}\right] \end{array}$$ (25)

As shown in Formula (25), ${\text{𝑜}}_{u}^{\left(\text{𝓈}\right)}\geq \text{0}$ is the Lagrange multiplier, and ρ>0 is the penalty coefficient. If $g_{u}^{\left(\text{𝓈}\right)}\leq \text{0}$ (constraints are satisfied), this term tends to be negative or small; if $g_{u}^{\left(\text{𝓈}\right)}>\text{0}$, this term generates a positive penalty and, through the combined effects of 𝑜 and ρ, forces the model to adjust the parameter θ to improve consistency. The overall training objective is to add a knowledge loss to the original translation and diffusion terms:


$$\begin{array}{c} L_{total}=L_{MT}+L_{diff}+\sum_{u=\text{1}}^{T}L_{K}^{\left(u\right)} \end{array}$$ (26)

This paper uses the augmented Lagrange relaxation method to transform equation (24) into a differentiable soft penalty term in equation (25). Compared with the traditional simple penalty term, the unique feature of this method is the introduction of dynamically updated Lagrange multipliers, the update strategy of which is shown in equation (27). When the generated trajectory deviates from the knowledge graph, the multiplier accumulates gradient pressure to force the model to adjust its parameters; when the trajectory conforms, the constraints are automatically relaxed to avoid overfitting. The dual ascent strategy is used to update the Lagrange multiplier:


$$\begin{array}{c} {\text{𝑜}}_{u}^{\left(\text{𝓈}\right)}\leftarrow max(\text{0},{\text{𝑜}}_{u}^{\left(\text{𝓈}\right)}+\rho g_{u}^{\left(\text{𝓈}\right)}) \end{array}$$ (27)

Constraints are gradually tightened or relaxed to avoid generation degradation caused by a single, forced hard constraint. To reduce computational burden, this loss is only applied to u in the later intervals. Knowledge confidence-based reranking during inference generates several candidate paths {𝓅} through the back-diffusion process. For each path, the model log probability $logp_{\theta }(\text{𝓅})$ and knowledge matching score are calculated as shown in Formula (28):


$$\begin{array}{c} KM(\text{𝓅})=\frac{\text{1}}{\left|C\right|}\sum_{\text{𝓈}\in C}{conf}_{\text{𝓈}}\cdot cos(r_{\text{0}}^{\left(\text{𝓈},\text{𝓅}\right)},e_{{\text{𝓀}}_{\text{𝓈}}}) \end{array}$$ (28)

$r_{\text{0}}^{\left(\text{𝓈},\text{𝓅}\right)}$ is the segmented representation of path 𝓅 at source location s in the final state u=0. The final reordering score is the weighted sum:


$$\begin{array}{c} Score(\text{𝓅})=logp_{\theta }(\text{𝓅}+\mu \cdot KM(\text{𝓅})) \end{array}$$ (29)

As shown in Formula (29), μ is a balancing term, reflecting the strength of the preference for knowledge consistency. The final output is selected according to Score(𝓅), thereby striking a balance between model confidence and knowledge matching, correcting the model’s bias in selecting long-tail cultural words.

**Fig 3:**
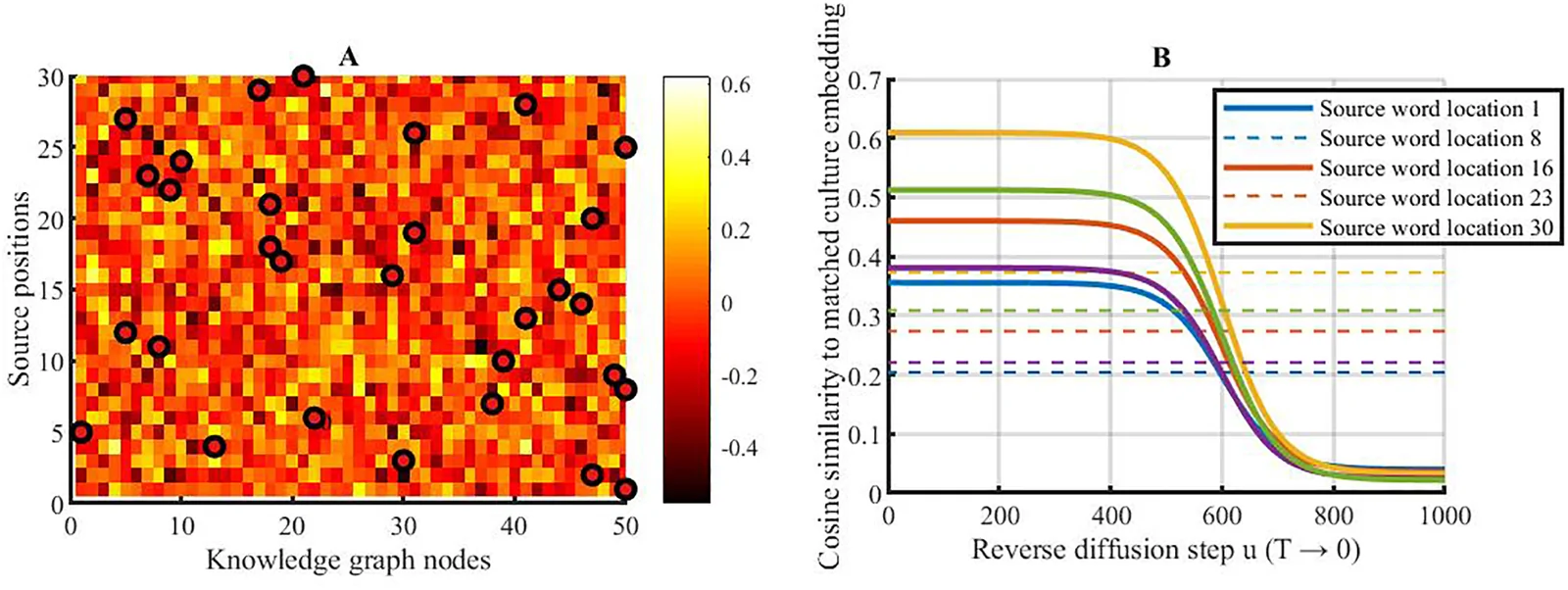
Cultural knowledge constraint mechanism. Panel A shows the matching of sparse conditional vectors and cultural embeddings. Panel B shows the knowledge consistency curve during the denoising process.

During the conditional modeling phase, an external cultural knowledge embedding with dimension $d_{c}=\text{30}$ is constructed. Sparse coding projection is performed on the locations of culturally loaded words in the source text, obtained through sensitivity screening. The cosine similarity matrix between these locations and the graph nodes is calculated, and matching points and their confidence levels are then annotated. This paper selects representative source locations and tracks the changes in their similarity with the corresponding knowledge embedding during the back-diffusion process, as shown in Fig 3:

As shown in Fig 3, proposed method primarily uses cultural knowledge to explicitly constrain long-tail and unregistered cultural terms during the translation diffusion process. In Fig 3A, the sparse conditional vectors at potentially culturally sensitive locations on the source side exhibit a clear similarity distribution with the external cultural knowledge embeddings. The model effectively captures the correspondence between culturally loaded words and graph nodes through sparse representation, demonstrating cross-cultural semantic consistency at high-confidence matching points. In Fig 3B, the similarity between different source locations gradually increases and stabilizes with the number of backdiffusion steps. The augmented Lagrangian constraint dynamically drives the generated trajectory toward cultural consistency.

### Multi-task joint training

The sparse coding reconstruction objective, the diffusion trajectory maximum likelihood objective, and the cultural knowledge consistency constraint are combined in a composite loss for end-to-end collaborative training. Through joint optimization, the sparse coding module, dictionary D, encoder/decoder parameters, and diffusion denoising network $p_{\theta }$ mutually constrain and improve under the same objective, achieving synergistic gains in the detection and generation of long-tail and unregistered culturally loaded words.

Suppose the source sequence $x=\left[x_{\text{1}},\cdots ,x_{\text{𝓈}}\right]$ is reconstructed based on sparse coding to obtain the dictionary D and sparse coefficients $\left\{a_{\text{𝓈}}\right\}$, and then truncate to obtain $\left\{{\tilde{a}}_{\text{𝓈}}\right\}$. The sparse coding reconstruction error is defined as the mean square error as shown in Formula (30):


$$\begin{array}{c} L_{sc}=\frac{\text{1}}{S}\sum_{s=\text{1}}^{S}{\left\|{\text{h}}_{\text{𝓈}}-D{\tilde{a}}_{\text{𝓈}}\right\|}_{\text{2}}^{\text{2}} \end{array}$$ (30)

This term drives the encoder output to converge toward a sparsely representable space, enhancing the semantic salience of culturally loaded words. The diffusion generation loss is based on a variational lower bound approximation: $L_{diff}=\sum_{u=\text{1}}^{T}E_{y,x_{u}\sim q(\cdot |y)}[-logp_{\theta }(x_{u-\text{1}}|x_{u},H,C)]$, which optimizes the decoder’s ability to gradually recover the target translation during multi-step denoising. The cultural knowledge consistency loss is based on an augmented Lagrangian form: $L_{kc}=\sum_{\text{𝓈}\in C}\left[{\text{𝑜}}_{u}^{\left(\text{𝓈}\right)}g_{u}^{\left(\text{𝓈}\right)}+\frac{\rho }{\text{2}}{\left(g_{u}^{\left(\text{𝓈}\right)}\right)}^{\text{2}}\right]$, which ensures that the generated trajectory continues to approximate known culturally equivalent patterns. Finally, the composite loss function is defined as the weighted sum of the three:


$$\begin{array}{c} L_{com}={\text{𝓌}}_{\text{1}}L_{sc}+{\text{𝓌}}_{\text{2}}L_{diff}+{\text{𝓌}}_{\text{3}}L_{kc} \end{array}$$ (31)

As shown in Formula (31), ${\text{𝓌}}_{\text{1}},{\text{𝓌}}_{\text{2}},{\text{𝓌}}_{\text{3}}$ are hyperparameters that control the contribution ratio of each task.

## Performance evaluation of culturally loaded word translation in news texts

### Experimental data

To verify the effectiveness of proposed method in translating culturally loaded words in English news texts, the paper used two widely used public bilingual news corpora as the baseline datasets: WMT(Workshop on Machine Translation)18 News Commentary v13 and TED (Translanguage English Database)Talks Parallel Corpus. The former, derived from news commentary, features formal language and a high concentration of culturally specific terms. The latter, derived from speech and dialogue, features highly colloquial expressions embedded with rich cultural metaphors and idioms, demonstrating strong cross-cultural communication characteristics. To enhance the breadth of coverage and accuracy of annotation for culturally loaded terms, this paper further selected sentences containing high-frequency and long-tail cultural terms from the aforementioned corpus and, combined with manual annotation, constructed a dedicated test subset. This collection consists of 500 source sentences and their reference translations, each containing at least one expert-verified culturally loaded term, covering four major categories: religion, politics, festivals, and social movements. The annotation was independently completed by three bilingual experts with cross-cultural research backgrounds. Culture-loaded words were defined as “words or phrases in the source language that carry specific social, historical, or value-based information, and whose direct translation may lead to the loss or misunderstanding of cultural information.” Each annotator first identified the location of culture-loaded words in the sentence and then provided a recommended reference translation. The Fleiss’ κ (Kappa) metric was used to calculate the consistency among the three annotators. On the culture-loaded word identification task, Fleiss’ κ = 0.838 (95% confidence interval [0.802, 0.901]), indicating good consistency. To ensure transparency and reproducibility, this paper selected an English-Chinese data subset from the WMT18 News Commentary v13 and an English-Chinese parallel corpus from the TED Talks Parallel Corpus. Table 3 shows basic information about the experimental dataset:

**Table 3:** Basic dataset information.

Classification	WMT18 News Commentary v13	TED Talks Parallel Corpus	Test subset
Source	News Commentary	Speech subtitles	Manual labeling/screening
Sentence Pair Size	200000	80000	500
Average Sentence Length (English)	24.6	22.1	31.2
Average Sentence Length (Chinese)	27.8	26.4	36.8
Culturally Loaded Word Density	14.3	18.6	39.8

This test subset contains 500 source language sentences and their reference translations. To evaluate the model’s ability to translate different types of culture-loaded words, these words are divided into four main categories: religious, political, festival, and social movement. These four categories of words appear frequently in English news texts and are culturally specific, covering multiple dimensions of material culture, spiritual culture, institutional culture, and contemporary cultural phenomena. In machine translation, these words have a high mistranslation rate and suffer significant loss of cultural information, making them representative and valuable for research. Table 4 shows the sample distribution of each category across the 500 sentences.

**Table 4:** Sample distribution of cultural categories in the test subset.

Cultural categories	Number of sentences	Percentage (%)
Religious	117	23.4
Political	156	31.2
Festival	104	20.8
Social movement	123	24.6
Total	500	100

Note: Some sentences may contain multiple categories of culture-loaded words. When making statistics, only the category to which the main culture-loaded word in the sentence belongs will be counted.

All text in data preprocessing is standardized. The SentencePiece algorithm is used for BPE (Byte Pair Encoding) word segmentation in both the source and target languages. To ensure the accurate labeling and recognition of culturally loaded words, this paper automatically tags potential culturally loaded words in the corpus using existing cross-cultural dictionaries and manual rules. This is combined with word frequency analysis and context-dependent correction to reduce mislabeling rates. Low-frequency words are sub-lexicalized while retaining their cultural semantic labels to prevent them from being overly diluted during training. Finally, the entire corpus is uniformly cleaned to remove noisy data, repeated sentence pairs, and unusually long or short sentence pairs. To demonstrate the statistical reliability of the conclusions drawn from this sample size, this study used the Bootstrap resampling method (10,000 resamplings) to calculate the 95% confidence interval of the complete model’s BLEU-4 score on the subset. The result was 27.8% ± 0.31%, and the difference between the complete model and the optimal baseline DiffuSeq was 3.8 percentage points. The interval width was much smaller than the minimum performance gap between models, indicating that the performance estimation under the current sample size has sufficient accuracy to support the comparison between models.

### Experimental setup

This model uses a Transformer-based framework, with a 6-layer encoder and decoder, a hidden dimension of 512, 8 attention heads, and a feedforward neural network dimension of 2048. The specific parameter settings are shown in Table 5:

**Table 5:** Model parameter settings.

Module	Parameter	Specifications
Sparse coding	Dictionary dimension	1024
	L1 regularization coefficient	1e^-4^
	Sparsity parameter	0.01
Diffusion generation	Time step	1000
	Time embedding dimension	64
	Lagrange multiplier initial value	0.1
	Penalty coefficient	0.01
Loss function	${\text{𝓌}}_{\text{1}}:{\text{𝓌}}_{\text{2}}:{\text{𝓌}}_{\text{3}}$	(1:2:0.5)
Model training	Optimizer	Adam
	Learning rate	2 × 10^−5^
	Batch size	64
	Gradient clipping threshold	1.0

After merging WMT18 News Commentary v13 with TED Talks Parallel Corpus, the samples were randomly divided into training, validation, and test sets in an 8:1:1 ratio. To ensure cross-set consistency in the distribution of cultural load words, a stratified sampling strategy was adopted, using the density of cultural load words in sentences as the stratification variable. All texts were standardized uniformly, and the BPE word segmentation model was trained using SentencePiece with a minimum word frequency of 2. The optimizer used was Adam ($\beta_{\text{1}}=\text{0}.\text{9},\beta_{\text{2}}=\text{0}.\text{9}\text{8}$), with a learning rate preheated to 2 × 10 − 5 (preheating steps 4000), followed by inverse square root decay. The batch size was 64, and the gradient clipping threshold was 1.0. All experiments were conducted with a fixed random seed (42) and independently trained on 5 different seeds (42, 123, 2024, 5678, 9999), reporting the mean and standard deviation to assess stability. The validation set BLEU test stopped if it did not improve for 5 consecutive epochs. The maximum number of epochs is 30. In the inference settings, the diffusion backward steps areT=1000, and linear noise scheduling is used. Iterative denoising starts from uniform noise, and the final translation is taken from the last sample result. The number of candidate paths is set to 5 during reordering, and the balance factor μ=0.3.

To comprehensively evaluate the performance of proposed method in translating culturally loaded words in English news texts, the paper conducted a validation study from three perspectives: overall translation quality, accuracy of culturally loaded word translation, and cultural adaptability. A comparative experiment was designed, encompassing three types of comparison models:

(1) Transformer

Transformer is used as the baseline model for news translation, relying solely on the self-attention mechanism to implement contextual dependency modeling.

(2) mBART-50

mBART-50 achieves multilingual transfer capabilities through cross-lingual pre-training, maintaining strong translation consistency in multilingual scenarios. Its generation process is based on direct decoding.

(3) DiffuSeq

DiffuSeq introduces a diffusion probability model into the sequence-to-sequence framework for text generation and generates target sequences through a step-by-step denoising process.

### Experimental results

#### Overall translation quality.

**Fig 4:**
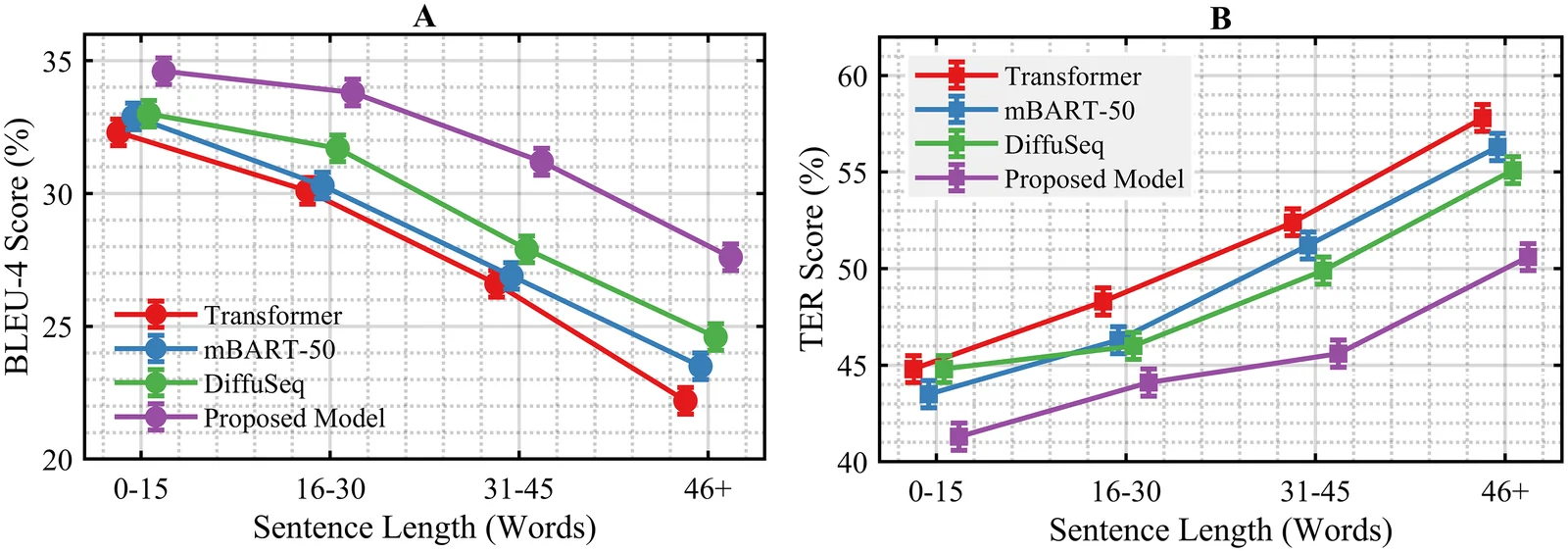
Comparison of BLEU-4 and TER metrics. Panel A shows the BLEU-4 results. Panel B shows the TER results.

To evaluate the overall translation quality of the model, this paper uses the BLEU-4 and TER (Translation Edit Rate) indicators for evaluation. BLEU-4 measures the consistency of word order and expression between the translation and the reference translation, reflecting the overall translation accuracy [31]. TER evaluates the translation error rate under different sentence length conditions by calculating the minimum number of editing operations [32]. This paper divides the experimental corpus into four intervals based on the length of the source sentence: 0–15 words, 16–30 words, 31–45 words, and 46 words and above, and tests it on the dataset. The comparison results are shown in Fig 4:

As shown in Fig 4, the proposed model significantly outperforms the three baseline models across all sentence lengths. In Fig 4A, the baseline model’s BLEU-4 performance decreases as sentence length increases. When the sentence length is 46 words or longer, the Transformer, mBART-50, and DiffuSeq performance drops to 22.1%, 23.2%, and 24.0%, respectively. The proposed model maintains a high level of 27.8%, 5.7%, 4.6%, and 3.8% higher than the other models, respectively, demonstrating its superior ability to retain contextual dependencies and culturally loaded words when processing long sentences. The TER metric in Fig 4B shows a similar trend, with the proposed model achieving the lowest TER values across all length ranges. For sentences longer than 46 words, its TER value is 50.8%, compared to 57.6%, 56.3%, and 54.9% for the other three comparison models, respectively. In comparison, the proposed model’s TER value decreases by 6.8%, 5.5%, and 4.1%, respectively, resulting in translations closer to the reference. Using sparse coding, the proposed model constructs a cultural semantic dictionary, enabling it to explicitly preserve the semantic characteristics of specific cultural vocabulary. Combined with diffusion generation, this approach strengthens sequence generation through progressive denoising, resulting in more accurate and coherent translations. Consequently, the proposed model maintains a high BLEU-4 score and low TER value for long sentence translation.

**Fig 5:**
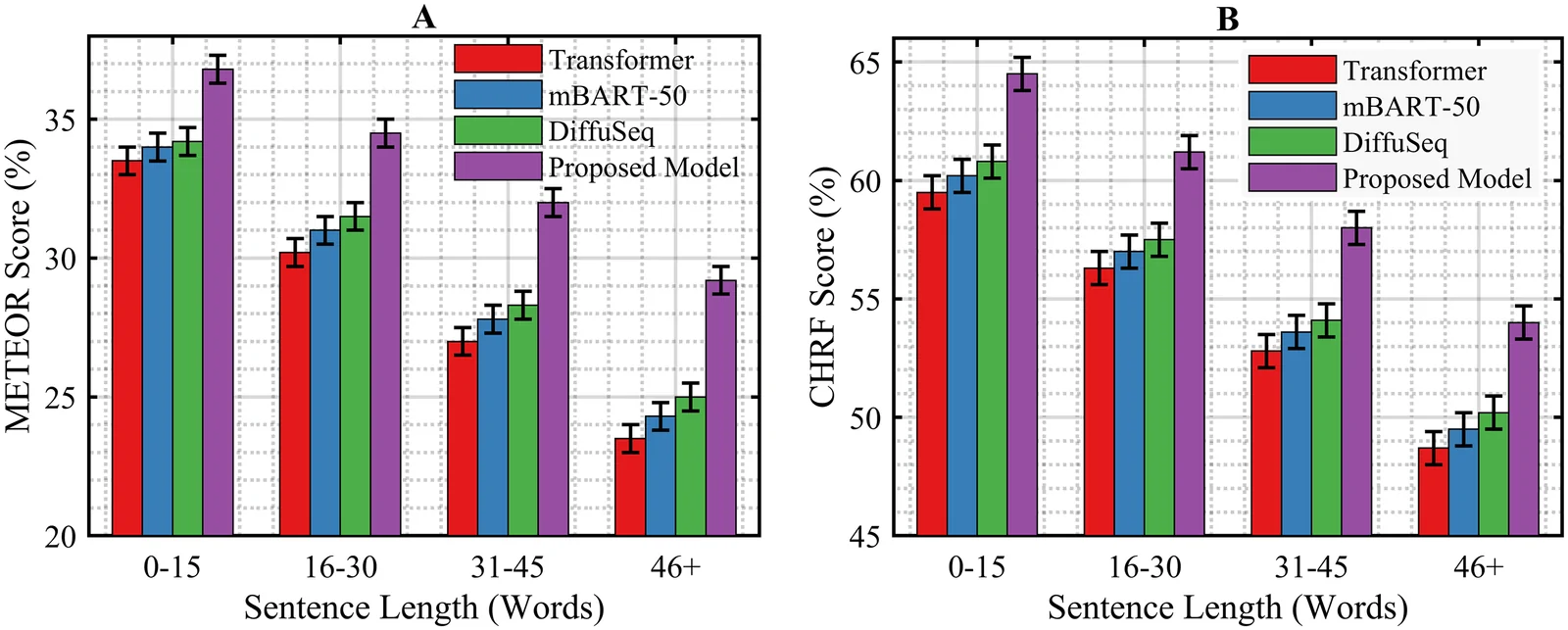
Comparison of METEOR and CHRF. Panel A shows the METEOR results. Panel B shows the CHRF results.

In order to further comprehensively evaluate the semantic fidelity and morphological adaptability of the model in non-literal translation tasks, two evaluation indicators, METEOR and CHRF (Character n-gram F-score), are introduced. METEOR can more sensitively reflect the accuracy of the translation in semantic transmission and cultural load word retention by comprehensively scoring word form changes, synonym matching and semantic alignment [33,34]; CHRF is based on character-level F-score calculation to evaluate the word segmentation and sub-word semantic retention in Chinese translation [35]. The results are shown in Fig 5:

As shown in Fig 5, the proposed model outperforms the comparison models in both METEOR and CHRF. In Fig 5A, when the sentence length exceeds 46 characters, the proposed model combining diffusion and sparse coding achieves a METEOR score of 29.2% in the translation task. The Transformer, mBART-50, and DiffuSeq achieved CHRF scores of 23.5%, 24.3%, and 25.0%, respectively. In Fig 5B, the proposed model achieved a CHRF score of 54.0% for translation tasks with a sentence length of 46 or more, compared to 48.7%, 49.5%, and 50.2%, respectively, for the comparison models. The Transformer performed the worst. While it relies on a self-attention mechanism to capture context, it has limited reliance on culturally loaded words and long sentences. While mBART-50 possesses multilingual transfer capabilities, it lacks a dedicated cultural information enhancement module, resulting in a loss of cultural metaphors. DiffuSeq is suboptimal. While it improves sequence coherence through diffuse generation, it still lacks the ability to identify culturally specific terms. Overall, the proposed model combines sparse coding with diffuse generation. This not only enhances the explicit modeling of culturally loaded words, but also optimizes the semantic coherence and morphological adaptability of the translated text through gradual generation, resulting in better translation performance for long sentences and those with high cultural density. This fully demonstrates the effectiveness of this method for cross-cultural translation tasks.

#### Translation accuracy of culturally loaded words.

**Fig 6:**
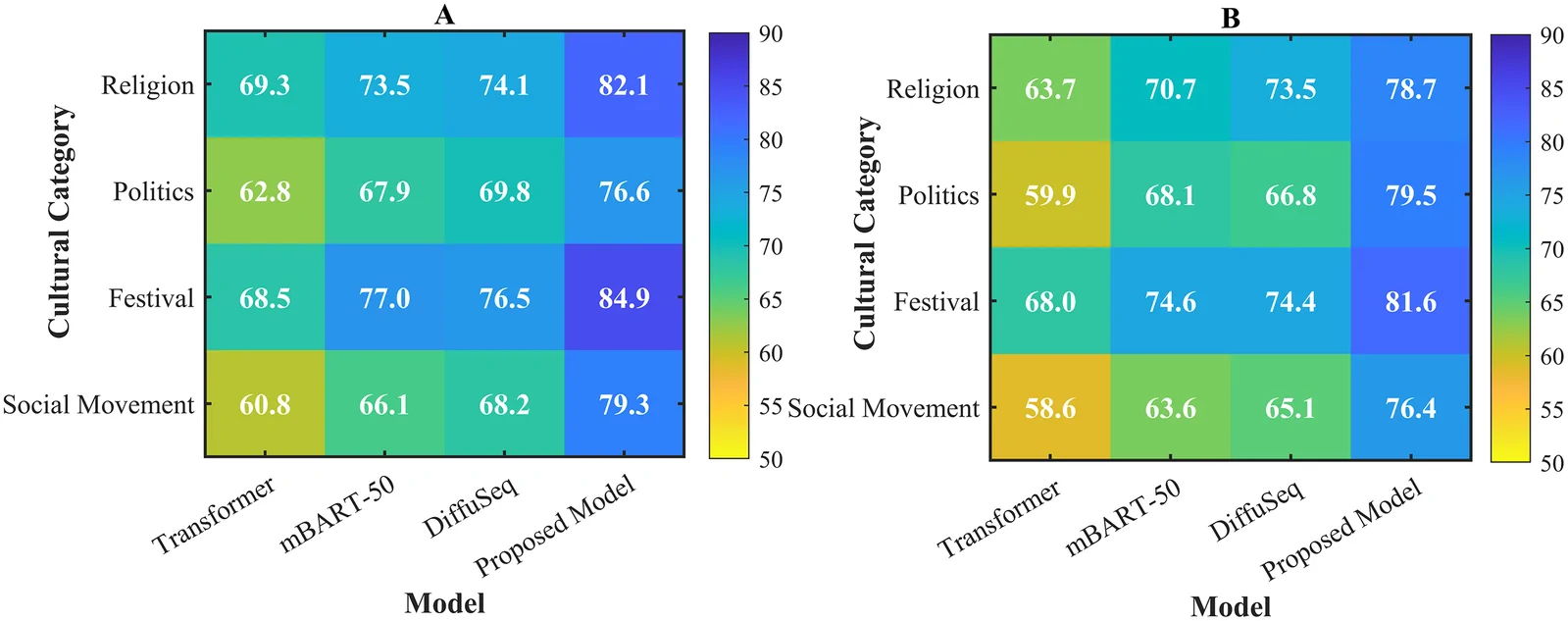
Comparison of accuracy and recall for different topics. Panel A shows the Accuracy results for different topics. Panel B shows the Recall results for different topics.

In terms of culturally loaded word translation accuracy, the paper used the accuracy and recall metrics. The Accuracy metric assesses the model’s correct translation percentage of identified culturally loaded words, while the Recall metric measures how many culturally loaded words appearing in the reference were correctly translated by the model. This compares the performance of different models on culturally loaded words across various topics in the test subset. This is shown in Fig 6.

The results in Fig 6 show that the proposed method significantly outperforms Transformer, mBART-50, and DiffuSeq in both Accuracy and Recall. In Fig 6A, the average Accuracy of the proposed model across all cultural topics reached 80.7%, while Transformer, mBART-50, and DiffuSeq achieved 65.4%, 71.1%, and 72.2%, respectively, demonstrating that the model is highly capable of identifying and translating culturally loaded words in cross-cultural scenarios. In Fig 6B, the average Recall of the proposed model was 79.1%, while the other three comparison models achieved 62.6%, 69.3%, and 70.0%, respectively. Transformer performed relatively poorly in translating cultural words across all topics, exhibiting relatively low levels of accuracy and recall. mBART-50 maintains a moderate performance across most categories thanks to its multilingual transfer capabilities. However, its lack of explicit modeling of specific cultural information leads to recall gaps in cross-cultural scenarios. DiffuSeq’s ability to accurately translate low-frequency cultural terms within a topic remains limited, and therefore underperforms the the proposed model. This translation model, combining a diffusion model with a sparse coding strategy, captures cultural feature vectors through sparse coding. It also incorporates a diffusion generation mechanism to gradually enhance the generation of culturally laden words in the target sequence. This approach preserves semantic and cultural information within the context, achieving a balance between high precision and high recall.

**Fig 7:**
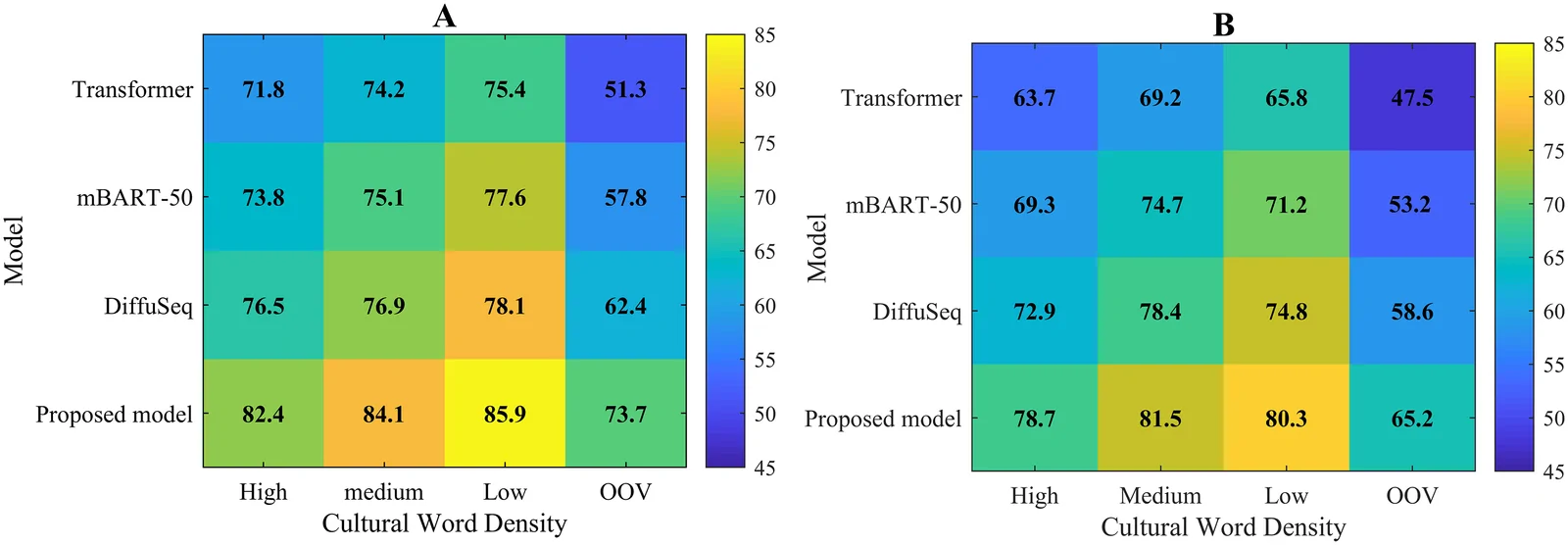
Comparison of accuracy and recall at different densities. Panel A shows the Accuracy at different densities. Panel B shows the Recall at different densities.

To further evaluate the model’s performance in translating culturally loaded words in English news text, this paper categorizes the culturally loaded words in the test subset into four categories based on sentence density: high density, medium density, low density, and out-of-scope (OOV). High-density sentences typically contain multiple culturally loaded words and are more challenging to translate; medium and low-density sentences are relatively sparse in cultural information but may contain some long-tail or unlisted words. OOV refers to culturally loaded words that do not appear in the training corpus. Accuracy and Recall metrics are also used to evaluate the translation performance of different models in each density category, as shown in Fig 7.

The results in Fig 7 clearly show significant differences in the accuracy and recall of culturally loaded word translations across different models. In Fig 7A, the proposed model achieves the best accuracy under all four density conditions. Its accuracy in the OOV vocabulary scenario is 73.7%, surpassing the Transformer, mBART-50, and DiffuSeq models by 22.4%, 15.9%, and 11.3%, respectively. In Fig 7B, the proposed model achieves a recall of 65.2% in the OOV vocabulary scenario, surpassing the other three models by 17.7%, 12.0%, and 6.6%. Overall, the proposed translation model, combining diffusion and sparse coding strategies, achieves superior translation results for unregistered words. This demonstrates the robustness of the proposed approach in context modeling and cross-cultural vocabulary recovery. In comparison, Transformer and mBART-50 maintain a reasonable level of accuracy at high, medium, and low densities, but exhibit lower accuracy and recall in out-of-view (OOV) scenarios, reflecting corpus dependency and insufficient generalization. DiffuSeq outperforms mBART-50 in OOV scenarios, but still falls short of the proposed model, demonstrating that relying solely on diffusion generation is insufficient to address the adaptation challenges for unregistered words. Overall, this model, through knowledge embedding alignment and cultural knowledge constraints, effectively mitigates semantic drift and cultural loss, improving the accuracy of loaded word translation.

#### Cultural adaptability.

**Fig 8:**
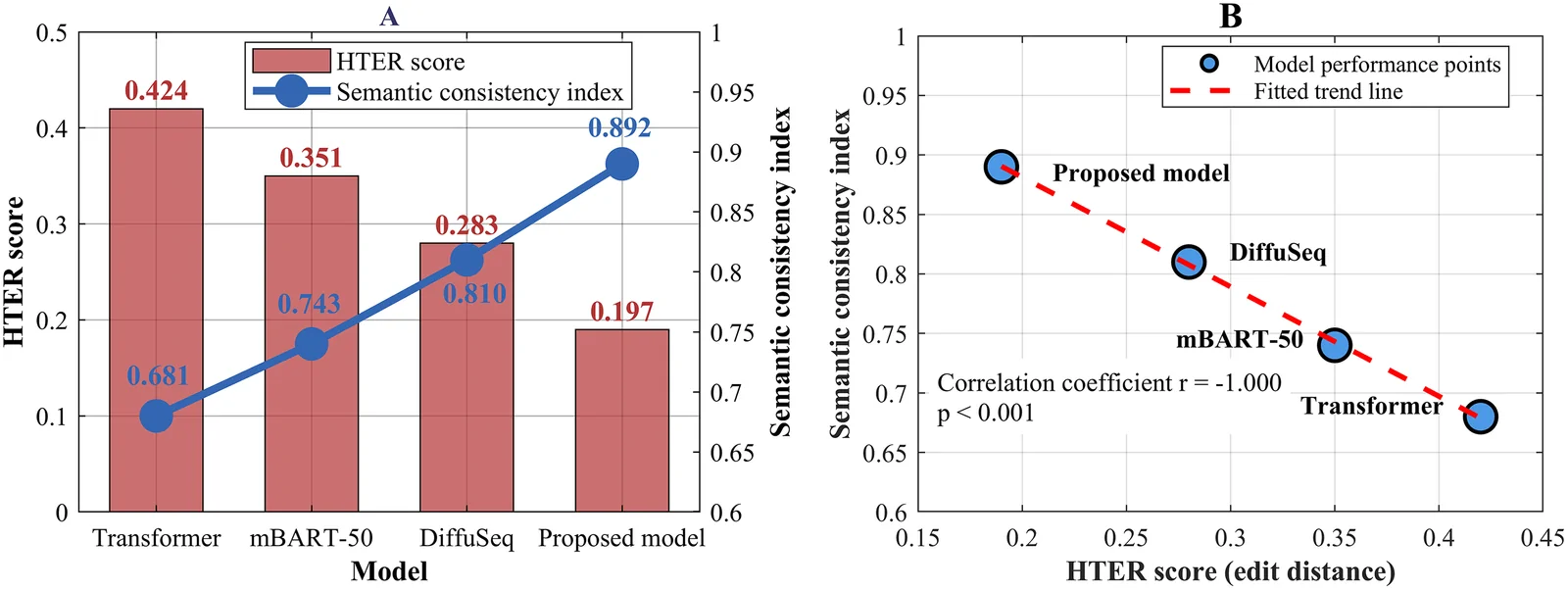
Comparison of cultural adaptability. Fig 8A shows the results of HTER and semantic consistency. Fig 8B shows the correlation analysis between HTER and semantic consistency.

In the cultural adaptability evaluation experiment, human translators first provided reference translations for the culturally loaded word samples in the test subset and annotated culturally relevant words and expressions. The Human Translation Error Rate (HTER) of each model on these samples was then calculated to measure the fidelity and integrity of cultural information at different density levels. Furthermore, a vector alignment-based Semantic Consistency Index (SCI) was used to calculate the similarity between the translation and the reference translation in terms of cultural knowledge using a multilingual semantic embedding model to reflect the degree of semantic alignment. Traditional metrics like BLEU and METEOR primarily assess the matching degree of translations at the lexical and phrase levels, making it difficult to capture the quality of cultural connotation transmission. This paper, however, uses SCI, a multilingual semantic embedding model, to directly measure the alignment between the translated text and the reference translation in the cultural knowledge space, specifically evaluating cultural semantic fidelity. While BLEU and METEOR reflect surface-level translation accuracy, SCI reflects deep-level cultural adaptability. The comparison results are shown in Fig 8:

In Fig 8, the translations of the proposed model achieve a more ideal alignment with the reference translations and authoritative cultural knowledge in the semantic embedding space. In Fig 8A, the proposed model achieved a HTER score of 0.197 on the test subset, a 53.5%, 43.9%, and 30.4% decrease compared to Transformer’s 0.424, mBART-50’s 0.351, and DiffuSeq’s 0.283, respectively. This demonstrates a significant improvement in the proposed model’s ability to faithfully convey culturally loaded words and related expressions. Furthermore, on the Semantic Consistency Index, the proposed model achieved a score of 0.892, 31.0%, 20.1%, and 10.1% higher than the three comparison models, respectively, demonstrating that the proposed model better preserves cultural context and semantic coherence. Fig 8B shows that the lower the edit distance, the higher the similarity between the translation and the reference in the semantic space, validating the effectiveness of HTER in reflecting cultural adaptability. Transformer and mBART-50 exhibit significant semantic drift for long-tail and unregistered cultural terms, resulting in high HTER and low semantic consistency. DiffuSeq enhances context modeling through progressive generation, but is still limited by the scarcity of training data, resulting in lower HTER and semantic consistency than the proposed model. This model combines sparse coding with a diffusion generation mechanism to achieve context completion and semantic restoration of culturally loaded terms, achieving optimal performance in both translation quality and cultural adaptability. Overall results demonstrate that the proposed method exhibits advantages in conveying cultural information and maintaining semantic fidelity in news texts, particularly when handling long-tail and unregistered culturally loaded words.

#### Statistical significance test and stability analysis.

To verify whether the improvement of our model relative to each baseline is statistically significant, all experiments were independently and repeatedly trained and tested using five different random seeds (42, 123, 2024, 5678, 9999), and the mean ± standard deviation of each indicator was calculated. For the differences between our model and Transformer, mBART-50, and DiffuSeq on the main indicators, a paired bootstrap test (10000 resamplings) was used to calculate the p-value, with a significance threshold of α = 0.05. Table 6 summarizes the complete statistical results for sentences longer than 46 words and OOV scenarios.

**Table 6:** Key indicators: mean ± standard deviation and statistical test results.

Evaluation indicators	This article’s model	Transformer	mBART-50	DiffuSeq	p-value (in this paper vs. baselines)
BLEU-4 (%)	27.8 ± 0.3	22.1 ± 0.5	23.2 ± 0.4	24.0 ± 0.4	<0.01 / < 0.01 / < 0.01
METEOR (%)	29.2 ± 0.4	23.5 ± 0.6	24.3 ± 0.5	25.0 ± 0.5	<0.01 / < 0.01 / < 0.01
TER (%)	50.8 ± 0.5	57.6 ± 0.7	56.3 ± 0.6	54.9 ± 0.6	<0.01 / < 0.01 / < 0.01
Accuracy (OOV) (%)	73.7 ± 1.2	51.3 ± 2.0	62.4 ± 1.5	57.8 ± 1.6	<0.01 / < 0.01 / < 0.01
Recall (OOV) (%)	65.2 ± 1.4	47.5 ± 2.2	53.2 ± 1.8	58.6 ± 1.5	<0.01 / < 0.01 / < 0.01
HTER	0.197 ± 0.011	0.424 ± 0.021	0.351 ± 0.018	0.283 ± 0.015	<0.01 / < 0.01 / < 0.01
SCI	0.892 ± 0.009	0.681 ± 0.014	0.743 ± 0.012	0.810 ± 0.010	<0.01 / < 0.01 / < 0.01

As shown in Table 6, our model significantly outperforms the three baselines across all metrics (p < 0.01) with a smaller standard deviation, indicating stable training. Particularly in the out-of-vocabulary (OV) scenario, Accuracy and Recall exceed the optimal baseline by approximately 15.9% and 6.6%, respectively, validating the effectiveness of the sparse coding and diffusion generation synergistic mechanism for long-tail culture-loaded words. The cultural fit metrics HTER and SCI also show the best performance, demonstrating that our method significantly improves the cross-cultural semantic fidelity of the translation.

#### Ablation experiment.

To accurately quantify the independent contributions of each core component—sparse coding, diffusion generation, time-step-aware gating, and cultural ontology constraints—to translation performance, this study designed five ablation variants and systematically evaluated them on two key test subsets: long sentences and out-of-vocabulary (OOV). All experiments were run five times independently with random seeds of 42, 123, 2024, 5678, and 9999. Mean ± standard deviation was reported, and the significance of differences was verified using a paired bootstrap test. The variant settings were as follows: w/o SC: sparse coding module removed, L1 regularization and dictionary learning disabled, directly using the Transformer encoder output as the conditional input; w/o Diff: diffusion decoding process replaced with a standard Transformer autoregressive decoder; w/o Gate: time-step-aware gating value fixed at 0.5, dynamic adjustment mechanism disabled; w/o KG: cultural knowledge graph constraints removed; Full: the complete model presented in this paper. The ablation experiment results and bootstrap test results are shown in Table 7 and Table 8.

**Table 7:** Ablation test results.

Model variants	BLEU-4 (%)	METEOR (%)	Accuracy (OOV) (%)	SCI
w/o SC	24.5 ± 0.4	25.8 ± 0.5	62.1 ± 1.4	0.801 ± 0.012
w/o Diff	25.1 ± 0.3	26.1 ± 0.4	64.3 ± 1.3	0.823 ± 0.011
w/o KG	26.2 ± 0.4	27.5 ± 0.5	68.0 ± 1.0	0.851 ± 0.010
w/o Gate	26.5 ± 0.3	27.8 ± 0.4	70.2 ± 1.1	0.868 ± 0.009
Full	27.8 ± 0.3	29.2 ± 0.4	73.7 ± 1.2	0.892 ± 0.009

**Table 8:** Paired bootstrap test p-value between complete model and ablation variant.

Comparison	BLEU-4	METEOR	Accuracy (OOV)	SCI
Full vs. w/o SC	< 0.001	< 0.001	< 0.001	< 0.001
Full vs. w/o Diff	< 0.001	< 0.001	< 0.001	< 0.001
Full vs. w/o KG	0.003	0.002	0.002	0.001
Full vs. w/o Gate	0.004	0.003	0.005	0.002

As shown in Table 7, the complete model outperforms any ablation variant on all metrics. Specifically, removing sparse coding (w/o SC) resulted in a 3.3 percentage point decrease in BLEU-4 and an 11.6% decrease in OOV accuracy, indicating that sparse coding is crucial for capturing low-frequency cultural semantics. Removing diffuse decoding (w/o Diff) reduced METEOR by 3.1% and SCI by 0.069, validating the positive impact of multi-step denoising generation on semantic coherence and cultural fit. The removal of knowledge graph constraints (w/o KG) and gating mechanisms (w/o Gate) also led to a 1.6% and 1.3% decrease in BLEU-4 and a loss in cultural consistency, respectively. The ablation results demonstrate that the performance improvement of the proposed model stems from the collaborative contributions of all components, rather than from a single module.

Table 8 shows that the differences between the full model and all ablation variants across all metrics were statistically significant (all p < 0.01), rejecting the null hypothesis and confirming that the performance improvement was not due to random fluctuations. Specifically, the differences between Full and w/o SC and w/o Diff were extremely significant (p < 0.001), reflecting that sparse coding and diffusion generation are the cornerstones of model performance. The p-values of Full and w/o KG and w/o Gate were relatively slightly higher (p < 0.005), but still far below the 0.05 threshold, indicating that although knowledge constraints and gating mechanisms contribute relatively little, the gains remain statistically robust and reliable.

#### External verification.

To further verify the generalizability of the conclusions, an additional 200 sentences containing culture-loaded words were randomly selected from the WMT19 news corpus as an external validation set, and the main experiment was repeated on this set. The results are shown in Table 9:

**Table 9:** External validation results.

Model	BLEU-4 (%)	METEOR (%)	TER (%)
This article’s model	27.2 ± 0.4	28.6 ± 0.5	51.5 ± 0.6
Transformer	21.8 ± 0.6	23.0 ± 0.7	58.2 ± 0.8
mBART-50	22.9 ± 0.5	23.8 ± 0.6	56.9 ± 0.7
DiffuSeq	23.7 ± 0.5	24.5 ± 0.6	55.4 ± 0.6

Note: Based on 5 independent runs (random seeds 42,123,2024,5678,9999), all paired bootstrap tests (10,000 resamplings) showed that the p-values of the differences between the model in this paper and each baseline were all < 0.01.

Table 9 presents the evaluation results on the independent external validation set. Our model significantly outperformed the three baselines in all three metrics (BLEU-4, METEOR, and TER) (p < 0.01), consistent with the findings of the main test set. While the BLEU-4 of our model (27.2%) was slightly lower than that of the main test set (27.8%), the advantage over the baselines remained stable, exceeding them by 5.4, 4.3, and 3.5 percentage points respectively, demonstrating the model’s good generalization ability to news corpora from different years and sources. The METEOR and TER metrics showed similar trends. The external validation results further support the effectiveness of our method, alleviating concerns about overfitting that might arise from the limited size of the main test set, and enhancing the reliability and generalizability of our research conclusions.

To further quantify the consistency between the conclusions of the external validation set and the main test set, this paper calculates the Pearson correlation coefficients of the BLEU scores of each model on the two datasets. For all four models, the BLEU scores of the main test set and the WMT19 external validation set are positively correlated (r = 0.787, p < 0.01), indicating that the diffusion-sparse cooperation mechanism significantly outperforms the baseline model and does not depend on the selection of a specific 500 sentences, demonstrating the robustness of the conclusions.

#### Correlation Verification between SCI and Artificial Cultural Judgment.

To verify the effectiveness of the semantic consistency index as an automatic assessment indicator of cultural fidelity, a manual evaluation correlation verification was conducted, examining the consistency between the SCI score and the cultural quality judgments of independent bilingual experts. From 500 sentences in the test subset, 100 samples were randomly selected stratified by cultural category. Three bilingual experts with cross-cultural communication research backgrounds who did not participate in the original annotation were invited to independently conduct blind evaluations of the translations generated by the four models. The evaluation was based on cultural information retention, i.e., the extent to which the translation accurately conveyed the social, historical, or value-based information carried by culturally loaded words in the source text. Each expert used a Likert scale of 1–5, where 1 = complete loss of cultural information, 3 = partial retention with bias, and 5 = complete and accurate retention of cultural information, to score each translation. The intraclass correlation coefficient (ICC) of the three experts on the randomly selected 50 samples was 0.876 (95% CI: [0.831, 0.914]), indicating good consistency in the evaluations. For each translation in the above 100 samples, its SCI score was calculated according to Formula (28). The Pearson correlation coefficient between SCI and manual CIR score under each model was also calculated, and the results are shown in Table 10.

**Table 10:** Correlation analysis results between SCI and CIR.

Model	Pearson r	95% CI	p-value	CIR
Transformer	0.883	[0.821, 0.927]	< 0.001	2.87 ± 0.52
mBART-50	0.902	[0.845, 0.941]	< 0.001	3.24 ± 0.48
DiffuSeq	0.918	[0.867, 0.951]	< 0.001	3.61 ± 0.43
Proposed	0.931	[0.885, 0.958]	< 0.001	4.38 ± 0.39
Four models merged	0.914	[0.887, 0.936]	< 0.001	–

Table 10 shows that SCI and human CIR scores were significantly positively correlated across all models (r = 0.914, p < 0.001), and the 95% CI lower bound was higher than 0.82, indicating that SCI can stably reflect human experts’ judgments on the degree of cultural information retention. The mean CIR of the model in this paper was 4.38, significantly higher than the optimal baseline DiffuSeq (p < 0.001), consistent with the ranking shown by the SCI index, further cross-validating the ability of SCI to distinguish cultural adaptability among models.

#### Computational overhead analysis of the inference phase.

The cultural ontology knowledge constraint proposed in this paper introduces additional computational overhead during the inference process, mainly in the following aspects: 1) knowledge matching and similarity calculation of the source cultural location in each reverse denoising step; 2) reordering calculation of the final candidate paths. In the standard T = 1000-step denoising process, the knowledge constraint is only calculated in the later denoising stage, i.e., the last 200 steps in this experiment, to balance efficiency and effectiveness. A single similarity calculation involves the dot product operation of low-dimensional vectors, with relatively small computational cost. The reordering stage requires calculating the knowledge matching score for each of the 5 candidate paths and reordering them. Based on the actual measurement estimate of a single NVIDIA V100, compared with the iterative denoising overhead of the diffusion model itself, the additional computational cost of the knowledge constraint part accounts for approximately 8% ~ 12% of the overall inference time. In practical deployment, the overhead can be further reduced by reducing the number of candidate paths or enabling knowledge reordering only on samples with low confidence. The computational burden brought by the ontology constraint is within an acceptable range and is exchanged for improved cultural adaptability, which has good practical value in scenarios that emphasize translation quality.

## Conclusion

To improve the accuracy of machine translation by addressing semantic drift and cultural information loss associated with long-tail and unregistered culturally loaded words in translated English news texts, this paper investigates machine translation using a combination of diffusion models and sparse coding strategies. By constructing a cultural semantic dictionary and an explicit sparse coding mechanism to capture the semantic characteristics of culturally loaded words, and combining diffusion generation with stepwise denoising to optimize sequence generation, the proposed model maintains coherence and accuracy in translations of long sentences and high cultural density contexts. In overall translation quality assessment, the proposed model significantly outperforms Transformer, mBART-50, and DiffuSeq in terms of BLEU-4, TER, METEOR, and CHRF metrics, particularly for long sentences and those with high cultural density. In terms of culturally loaded word translation accuracy, the average Accuracy and Recall across different topics reached 80.7% and 79.1%, respectively, maintaining excellent performance in high, medium, and low-density and OOV scenarios. In the cultural adaptability evaluation, the HTER reached only 0.197, and the Semantic Consistency Index reached 0.892, validating the model’s advantages in cultural fidelity and semantic alignment. The method in this paper not only improves the translation accuracy and recall of culturally loaded words in news texts, but also enhances the semantic fidelity and cultural adaptability of the translation in a cross-cultural context, providing an effective strategy and feasibility reference for the application of machine translation in complex cross-cultural tasks. However, this paper still has some limitations. The specific test subset is relatively small. Although external validation enhances the reliability of the conclusions, further validation on a larger scale and with more languages is needed in the future. The experimental evaluation in this study is based on English-Chinese news parallel corpora and four categories of culturally loaded words: religion, politics, festivals, and social movements. The empirical support for the current conclusions is limited to this scope. Methodologically, the collaborative framework of sparse encoding, diffusion generation, and knowledge constraints in this paper is designed to be language-independent. Its direct applicability is limited to the four categories of cultural vocabulary in the aforementioned English-Chinese news scenarios. Theoretically, it can be transferred to other language pairs such as English-Japanese and English-Arabic, but the transfer effect depends on the completeness of the cultural ontology of the target language pair and the scale of the training data. For other news subdomains such as finance, technology, and entertainment, or language pairs with significant morphological differences, such as English-Japanese and English-Arabic, validation needs to be performed after expanding the cultural ontology and evaluation samples. For language pairs with limited resources, cross-language pre-trained models can be used for initialization. For language pairs with complex forms or significant cultural differences, this method may need further adjustments. Future work will construct a subset of multilingual cultural assessments, explore strategies for integrating cross-lingual pre-trained models with the current framework, and empirically verify the cross-lingual universality of the framework.

## Supporting information

S1 File Python source code for the combined diffusion model and sparse coding strategy. This file contains the complete implementation of the proposed framework, including model architecture, sparse regularization module, discrete diffusion decoder, time-step-aware gating mechanism, cultural ontology embedding alignment, and the end-to-end training pipeline. All core modules (data processing, training, evaluation, and result visualization) are provided to ensure the reproducibility of the experimental results reported in this paper.
(ZIP)
