# Supplementary material for: Combining a diffusion model with a sparse coding strategy to improve the machine translation accuracy of culturally loaded words in English news texts
Source: PLoS One. 2026 Aug 28;21(8):e0356146. doi: 10.1371/journal.pone.0356146 (PMC13524296; doi:10.1371/journal.pone.0356146)
Supplement: Supplementary file 1 [file pone.0356146.s003.docx]

Dear Dr. Yu,

Thank you for submitting your manuscript to PLOS ONE. After careful consideration, we feel that it has merit but does not fully meet PLOS ONE’s publication criteria as it currently stands. Therefore, we invite you to submit a revised version of the manuscript that addresses the points raised during the review process.

==============================
While the reviewers agree that the topic is potentially interesting, the paper in its current form requires substantial revision before it can be considered further. The main concern is that the experimental evidence does not yet support the strength of the claims. The revised manuscript should report variability across repeated runs, confidence intervals, and appropriate significance testing for the reported results. It should also include clear ablation studies to separate the contribution of each major component, including sparse coding, the diffusion mechanism, and the gating module. In addition, the evaluation needs to be strengthened through broader validation, ideally with a larger or external dataset beyond the current 500-sentence manually constructed subset. The reviewers also identified important issues related to reproducibility, clarity, and presentation.

Please include the following items when submitting your revised manuscript:

- A letter that responds to each point raised by the academic editor and reviewer(s). You should upload this letter as a separate file labeled 'Response to Reviewers'.
- A marked-up copy of your manuscript that highlights changes made to the original version. You should upload this as a separate file labeled 'Revised Manuscript with Track Changes'.
- An unmarked version of your revised paper without tracked changes. You should upload this as a separate file labeled 'Manuscript'.

Journal Requirements:

[When submitting your revision, we need you to address these additional requirements.

1. Please ensure that your manuscript meets PLOS ONE's style requirements, including those for file naming. The PLOS ONE style templates can be found at
https://journals.plos.org/plosone/s/file?id=wjVg/PLOSOne_formatting_sample_main_body.pdf and
https://journals.plos.org/plosone/s/file?id=ba62/PLOSOne_formatting_sample_title_authors_affiliations.pdf](C:/Users/e753958/Desktop/PRTC Send backs/1-21-26/When submitting your revision, we need you to address these additional requirements.1. Please ensure that your manuscript meets PLOS ONE's style requirements, including those for file naming. The PLOS ONE style templates can be found at https:/journals.plos.org/plosone/s/file?id=wjVg\\PLOSOne_formatting_sample_main_body.pdf and https:\\\\journals.plos.org\\plosone\\s\\file?id=ba62\\PLOSOne_formatting_sample_title_authors_affiliations.pdf)

Reply: Thank you for your guidance. We have revised the document item by item according to the PLOS ONE template, and adjusted the headings of the abstract, introduction, methods, results, and conclusions; the citation format of the references has been standardized.

2. Please note that PLOS One has specific guidelines on code sharing for submissions in which author-generated code underpins the findings in the manuscript. In these cases, we expect all author-generated code to be made available without restrictions upon publication of the work. Please review our guidelines at https://journals.plos.org/plosone/s/materials-and-software-sharing#loc-sharing-code and ensure that your code is shared in a way that follows best practice and facilitates reproducibility and reuse.

Reply: Thank you for reminding us of PLOS ONE's code-sharing policy. We have carefully read the relevant guidelines. The self-written code used for model training, evaluation, and analysis in this study, along with its algorithmic logic and key parameters, are fully reported in Chapters 2 and 3 of the manuscript, in the Methods and Experimental Setup sections, to support the reproduction of the findings.

3. Please provide a complete Data Availability Statement in the submission form, ensuring you include all necessary access information or a reason for why you are unable to make your data freely accessible. If your research concerns only data provided within your submission, please write "All data are in the manuscript and/or supporting information files" as your Data Availability Statement.

Reply: Thank you for your reminder. In accordance with the PLOS ONE data availability policy, we have added a complete data availability statement to the manuscript: WMT18 News Commentary v13 and TED Talks Parallel Corpus used in this study are publicly available bilingual corpora, accessible through their official websites.

4. PLOS requires an ORCID iD for the corresponding author in Editorial Manager on papers submitted after December 6th, 2016. Please ensure that you have an ORCID iD and that it is validated in Editorial Manager. To do this, go to ‘Update my Information’ (in the upper left-hand corner of the main menu), and click on the Fetch/Validate link next to the ORCID field. This will take you to the ORCID site and allow you to create a new iD or authenticate a pre-existing iD in Editorial Manager.

Reply: Thank you for your reminder. I have updated my Information in the main menu.

5. If the reviewer comments include a recommendation to cite specific previously published works, please review and evaluate these publications to determine whether they are relevant and should be cited. There is no requirement to cite these works unless the editor has indicated otherwise.

Reply: Thank you for your valuable suggestions. We have carefully reviewed the cited literature and assessed its relevance to our core contribution of combining Diffusion models with sparse coding to improve the translation of culture-loaded words, without blindly adding irrelevant citations.

Reviewers' comments:

Reviewer's Responses to Questions

**Comments to the Author**

1. Is the manuscript technically sound, and do the data support the conclusions?

The manuscript must describe a technically sound piece of scientific research with data that supports the conclusions. Experiments must have been conducted rigorously, with appropriate controls, replication, and sample sizes. The conclusions must be drawn appropriately based on the data presented.

Reviewer #1: No

Reviewer #2: No

2. Has the statistical analysis been performed appropriately and rigorously?

Reviewer #1: No

Reviewer #2: No

3. Have the authors made all data underlying the findings in their manuscript fully available?

The [PLOS Data policy](http://track.editorialmanager.com/CL0/http://www.plosone.org/static/policies.action%23sharing/1/010f019d703eb033-a6bdfa6d-837b-4bdf-9369-a55046c6455b-000000/B0FgIMIMoy0JPDMzoQCzkupDMdG1-gYObtDKqP04OoM=258) requires authors to make all data underlying the findings described in their manuscript fully available without restriction, with rare exception (please refer to the Data Availability Statement in the manuscript PDF file). The data should be provided as part of the manuscript or its supporting information, or deposited to a public repository. For example, in addition to summary statistics, the data points behind means, medians and variance measures should be available. If there are restrictions on publicly sharing data—e.g. participant privacy or use of data from a third party—those must be specified.

Reviewer #1: No

Reviewer #2: No

4. Is the manuscript presented in an intelligible fashion and written in standard English?

PLOS ONE does not copyedit accepted manuscripts, so the language in submitted articles must be clear, correct, and unambiguous. Any typographical or grammatical errors should be corrected at revision, so please note any specific errors here.

Reviewer #1: No

Reviewer #2: No

5. Review Comments to the Author

Please use the space provided to explain your answers to the questions above. You may also include additional comments for the author, including concerns about dual publication, research ethics, or publication ethics. (Please upload your review as an attachment if it exceeds 20,000 characters)

Reviewer #1: 1-Details of the proposed model in the manuscript are not sufficient to reproduce it. Important factors, such as the data splits, preprocessing pipeline, random seeds and training stability across multiple runs are absent. However, the lack of reproducibility documentation undermines the scientific validity of the work despite combining sparse coding, diffusion and knowledge constraints.
Reply: Thank you for your suggestions. We have revised the manuscript accordingly: in Section 3.2, “Experimental Setup,” we have added details on data partitioning and preprocessing, stratified sampling strategy, and a random seed list (42,123,2024,5678,9999), which improves the scientific validity and transparency of the study.

2-All reported results (BLEU, METEOR, TER, Accuracy, Recall, HTER SCI) are show as single values with no variance, confidence intervals or statistical tests. There was no evidence that the respective improvements over Transformer, mBART-50 and DiffuSeq were statistically significant which casts doubt on the veracity of their conclusions.
  Reply: Based on your suggestion, we have revised Section 3.3, adding Section 3.3.4 for statistical significance testing and stability analysis, which summarizes the statistical results of each indicator. All improvements are statistically significant (p<0.01) and have small standard deviations, proving that the model is stable and reliable.
  3-The manuscript integrates several complex components (sparse coding + diffusion decoding + cultural knowledge graph + gating mechanism), without any ablation studies that could isolate the effects of individual modules. Thus it is not obvious whether the performance benefits are due to the full architecture or just certain components of it.
  Reply: Thank you for pointing out the necessity of the ablation experiments. We have added "ablation experiments" as suggested, designing five variants to remove sparse coding, diffusion decoding, knowledge graph constraints, and gating mechanisms, respectively. The results show that the complete model outperforms any ablation variant on all metrics, especially in BLEU-4 and OOV accuracy, validating the overall effectiveness of our proposed architecture.
  4-The details regarding annotation methodology, inter-annotator agreement or bias control are absent in the manually constructed test subset (500 sentences). This raises potential for low reliability of evaluation between both people and cultures, especially genuine and subjective words.
  Reply: In response to your question, we have supplemented Section 3.1 with the process for constructing and annotating the test subset: including independent annotation by three bilingual experts and Fleiss's K consistency, which improves the transparency of data construction and the reliability of evaluation results, and ensures the effectiveness and reproducibility of the test subset in different cultural contexts.
  5-Access to the processed datasets, annotated subset and intermediate data in experiments is not provided. The Data Availability statement restricts access without appropriate reasons given, which is in conflict with open science practices and PLOS data-sharing policies.
  Reply: Thank you for your feedback. We have revised the "Data Availability Statement": It now clarifies that the WMT18 and TED corpora used in this study are all publicly available resources; the construction method and annotation process of the dedicated test subset have been fully described in Section 3.1; all preprocessing, word segmentation, data partitioning, and other parameters are detailed in Section 3.2, and you can reproduce all the results yourself by following the detailed steps provided in the text.
  6-The maths are not clearly or consistently defined -- a number of the equations and model components (e.g., sparse coding formulation, gating mechanism, diffusion transition) are revealed but without in instructions that are sufficient. (Notation is sometimes ambiguous and dimensional consistency not always apparent, which hinders full comprehension/imposed of the method.)
  Reply: Thank you for pointing out the shortcomings in the formula description. We have added "Symbol Explanation" to Chapter 2 as suggested, unifying all variable symbols, dimensions, and value ranges; added dimension annotations and parameter explanations to the key formulas for sparse coding, gating mechanisms, and diffusion transfer; and corrected any ambiguities and inconsistencies in the symbols.
  7-Despite being pitched as an important advance the manuscript itself fails to motivate how their contribution is new beyond an amalgamation of techniques (Transformer, sparse coding diffusion models knowledge graph). The novelty is not well enough defined, and claims need to be toned down or better justified.

Reply: Thank you for pointing out the lack of innovativeness in our description. We have clearly identified three core innovations at the end of the introduction: conditional coupling of sparse semantic anchors and diffusion trajectories, time-step-aware dynamic gating, and augmented Lagrangian soft-constraint knowledge alignment, and have demonstrated the synergistic effect of these three elements. At the same time, we have downplayed terms such as "combination" to highlight the originality of the mechanism and distinguish our contributions from existing work.

Reviewer #2: The manuscript proposes a hybrid framework combining diffusion models, sparse coding, and cultural ontology constraints to improve machine translation of culturally loaded words in English news texts. The topic is relevant and the integration of multiple techniques is interesting. However, several methodological and presentation issues limit the current strength of the work.

First, while the proposed architecture is technically elaborate, the manuscript lacks sufficient experimental rigor to support the claimed improvements. Although the results indicate performance gains over baseline models (e.g., Transformer, mBART-50, DiffuSeq), the improvements are relatively moderate and are not supported by statistical significance testing. The manuscript does not report confidence intervals, variance across runs, or results from multiple random seeds, which makes it difficult to assess the robustness of the findings.
Reply: Regarding your point about insufficient statistical rigor, we have supplemented Section 3.3 with the mean ± standard deviation of the main indicators and the results of statistical tests, using paired bootstrap tests to calculate p-values. The results show that the improvement of our model is statistically significant, and the standard deviation is small, validating the robustness of the results.

Second, the study does not include ablation experiments to isolate the contributions of the individual components (sparse coding, diffusion mechanism, and cultural knowledge constraints). Without such analysis, it is unclear which component is primarily responsible for the observed performance gains.
Reply: Thank you for pointing out the necessity of the ablation experiments. We have added Section 3.3, "Ablation Experiments," and designed five variants to remove sparse coding, diffusion decoding, knowledge graph constraints, and gating mechanisms, respectively. The results show that the complete model outperforms any ablation variant on all metrics, with each component making a key contribution, clearly separating and quantifying the role of each module.

Third, the evaluation dataset is limited, particularly the manually constructed test subset of 500 sentences. While this dataset is useful for focused evaluation of culturally loaded words, its size may not be sufficient to support strong general conclusions. Additional large-scale or external validation datasets would strengthen the study.
Reply: Based on your suggestion, we have added external validation analysis to Section 3.1. The results show that our model achieves BLEU-4 accuracy of 27.2% ± 0.4%, significantly better than the baseline (p < 0.01), consistent with the conclusions of the main test set. Furthermore, the limitations of the dataset and future expansion directions are discussed in the conclusions, enhancing the reliability of the findings.

Fourth, the manuscript lacks clarity in methodological explanation. While the mathematical formulation is detailed, several components (e.g., gating mechanism, knowledge constraint integration) are not explained intuitively, making it difficult for readers to fully understand the model design and its practical implications.
Reply: Based on your suggestion, we have used natural language to explain the collaborative logic of sparse anchor extraction, cultural condition construction, diffusion denoising and gating mechanism, and knowledge constraint calibration step by step at the beginning of Section 2, which improves the understandability of the model design and the transparency of practical applications.

Fifth, the data availability statement indicates restrictions, which limits reproducibility. The authors are encouraged to provide clearer information regarding dataset access, preprocessing steps, and implementation details to facilitate replication.
Reply: Based on your suggestion, we have detailed the dataset source, BPE segmentation, cleaning rule preprocessing steps, and implementation parameters such as learning rate, batch size, and random seed in Sections 3.1 and 3.2, and added a data availability declaration to clearly indicate the method description and ensure reproducibility.

Finally, the manuscript requires substantial language editing. There are multiple grammatical issues, repetitive phrasing, and sections that are difficult to follow, which reduce readability.
Reply: Based on your suggestions, we have polished the entire text: correcting grammatical errors and repetitive expressions, and improving readability and professionalism. Thank you again for your meticulous corrections.

In summary, while the proposed approach is conceptually interesting and has potential, the manuscript requires significant improvements in experimental validation, clarity, and reproducibility before it can be considered for publication.

Reply: Thank you for your overall evaluation and constructive criticism. We have made improvements in three areas: experimental validation, clarity, and reproducibility. We have added statistical significance tests, ablation experiments, and external validation sets, and improved the data availability statement and formula symbol explanations.
