# Supplementary material for: Combining a diffusion model with a sparse coding strategy to improve the machine translation accuracy of culturally loaded words in English news texts
Source: PLoS One. 2026 Aug 28;21(8):e0356146. doi: 10.1371/journal.pone.0356146 (PMC13524296; doi:10.1371/journal.pone.0356146)
Supplement: Supplementary file 2 [file pone.0356146.s004.docx]

Journal Requirements:

If the reviewer comments include a recommendation to cite specific previously published works, please review and evaluate these publications to determine whether they are relevant and should be cited. There is no requirement to cite these works unless the editor has indicated otherwise. 

Please review your reference list to ensure that it is complete and correct. If you have cited papers that have been retracted, please include the rationale for doing so in the manuscript text, or remove these references and replace them with relevant current references. Any changes to the reference list should be mentioned in the rebuttal letter that accompanies your revised manuscript. If you need to cite a retracted article, indicate the article’s retracted status in the References list and also include a citation and full reference for the retraction notice.

**Additional Editor Comments:**

Kindly address the new comments regarding clarification and manuscript presentation. In addition, please ensure that all raw and source data are deposited in a publicly accessible repository, in accordance with standard data availability requirements.


[Note: HTML markup is below. Please do not edit.]

Reviewers' comments:

Reviewer's Responses to Questions

**Comments to the Author**

1. If the authors have adequately addressed your comments raised in a previous round of review and you feel that this manuscript is now acceptable for publication, you may indicate that here to bypass the “Comments to the Author” section, enter your conflict of interest statement in the “Confidential to Editor” section, and submit your "Accept" recommendation.

Reviewer #1: All comments have been addressed

2. Is the manuscript technically sound, and do the data support the conclusions?

The manuscript must describe a technically sound piece of scientific research with data that supports the conclusions. Experiments must have been conducted rigorously, with appropriate controls, replication, and sample sizes. The conclusions must be drawn appropriately based on the data presented.

Reviewer #1: Yes

3. Has the statistical analysis been performed appropriately and rigorously?

Reviewer #1: Yes

4. Have the authors made all data underlying the findings in their manuscript fully available?

The [PLOS Data policy](http://track.editorialmanager.com/CL0/http://www.plosone.org/static/policies.action%23sharing/1/010f019e8216b7b1-3c893985-ca8d-47fd-adb9-42385c629653-000000/VKzNt42-l4J_DCEAkiwKN71SC8IbqD3wVXj-Sty5N1c=258) requires authors to make all data underlying the findings described in their manuscript fully available without restriction, with rare exception (please refer to the Data Availability Statement in the manuscript PDF file). The data should be provided as part of the manuscript or its supporting information, or deposited to a public repository. For example, in addition to summary statistics, the data points behind means, medians and variance measures should be available. If there are restrictions on publicly sharing data—e.g. participant privacy or use of data from a third party—those must be specified.

Reviewer #1: Yes

5. Is the manuscript presented in an intelligible fashion and written in standard English?

PLOS ONE does not copyedit accepted manuscripts, so the language in submitted articles must be clear, correct, and unambiguous. Any typographical or grammatical errors should be corrected at revision, so please note any specific errors here.

Reviewer #1: Yes

6. Review Comments to the Author

Please use the space provided to explain your answers to the questions above. You may also include additional comments for the author, including concerns about dual publication, research ethics, or publication ethics. (Please upload your review as an attachment if it exceeds 20,000 characters)

Reviewer #1: 1-Please provide a brief summary table describing the cultural ontology knowledge graph, including the number of entities, relation types, and major cultural categories represented in the ontology.
Reply: Thank you for your suggestion. We have added Table 2 to Section 3.3, which briefly summarizes the cultural ontology knowledge graph. This ontology is constructed based on the cultural load words that actually appeared in the test subset of this experiment. It contains 186 entity nodes and 312 relation edges, covering four types of relations: equivalence, hierarchical, association, and contrast. It involves cultural categories such as religion, politics, festivals and social movements.
2-The discussion of the Cultural Semantic Consistency Index (SCI) can be strengthened by briefly explaining how SCI complements traditional metrics such as BLEU and METEOR in evaluating cultural translation quality.
Reply: Based on your suggestion, we have supplemented Section 4.3 with an explanation of the complementary relationship between the Cultural Semantic Consistency Index (SCI) and traditional metrics BLEU and METEOR. BLEU and METEOR focus on lexical and phrase-level matching, making it difficult to quantify the quality of cultural connotation delivery; while SCI, through a multilingual semantic embedding model, directly measures the alignment of the translation within the cultural knowledge space, specifically assessing cultural fidelity. Combining these three approaches provides a more comprehensive characterization of the model's overall performance in cross-cultural translation tasks.
3-Please include a short discussion on the computational overhead introduced by ontology constraints during inference, even if only in qualitative terms, to improve understanding of practical deployment considerations.
Reply: Thank you for your suggestion. We have added a qualitative discussion regarding the computational overhead of ontology-constrained inference in the last section of the experimental part. Knowledge constraints are only activated in the last 200 denoising steps, and each computation is a dot product operation of a 30-dimensional vector, with minimal overhead; reordering only applies to 5 candidate paths. Compared to the iterative denoising overhead of the diffusion model itself, the knowledge constraint part accounts for approximately 8%~12% of the overall inference time, which is within an acceptable range. In practical deployments, further optimization can be achieved by reducing the number of candidate paths or selectively enabling constraints.
4-The description of the manually constructed cultural evaluation subset would benefit from additional details regarding the distribution of samples across the four cultural categories and the rationale for category selection.
Reply: Based on your suggestion, we have supplemented Section 3.1 with details of the cultural category distribution of the test subset. The selection of the four categories—religion, politics, festivals, and social movements—is based on their representativeness in high-frequency English news texts, the strength of their cultural specificity, and the current situation where cultural information is easily lost in machine translation. The religious category focuses on spiritual culture, the political category on institutional culture, the festival category on customary culture, and the social movement category on contemporary cultural phenomena.
5-Please clarify whether the ontology resources and cultural evaluation subset will be made publicly available after publication to facilitate future research and reproducibility.
Reply: Thank you for your attention. Regarding the accessibility of ontology resources and cultural evaluation subsets, due to the involvement of multiple parties and usage agreements, no publicly available channels have been established for their release. To meet the needs of researchers seeking to reproduce and extend these resources, we have detailed the ontology construction process, entity and relation definitions, annotation standards, and sampling strategies in our paper. Researchers requiring further information or to request access can contact us via the corresponding author's email address; we will provide assistance within the scope permitted by the cooperation agreement. This section has been supplemented in the data availability statement.
6-The future research section could be improved by briefly discussing multilingual extension possibilities, particularly the applicability of the proposed framework to language pairs beyond English–Chinese translation.

Reply: Following your guidance, we have supplemented the conclusion with a discussion on multilingual extensions. Specifically, the proposed sparse coding and diffusion generation synergistic mechanism is language-independent: the sparse coding module does not rely on specific language priors, and the diffusion decoder can be extended by constructing multilingual cultural ontologies. This framework can be directly transferred to other language pairs such as English-Japanese and English-Arabic. For low-resource language pairs, cross-lingual pre-trained models can be used for initialization. We also emphasize the need for further adaptation for language pairs with complex morphology or large cultural distances, and related research will be an important direction for future work.
