# Supplementary material for: Combining a diffusion model with a sparse coding strategy to improve the machine translation accuracy of culturally loaded words in English news texts
Source: PLoS One. 2026 Aug 28;21(8):e0356146. doi: 10.1371/journal.pone.0356146 (PMC13524296; doi:10.1371/journal.pone.0356146)
Supplement: Supplementary file 3 [file pone.0356146.s005.docx]

Dear Dr. Yu,

Thank you for submitting your manuscript to PLOS ONE. After careful consideration, we feel that it has merit but does not fully meet PLOS ONE’s publication criteria as it currently stands. Therefore, we invite you to submit a revised version of the manuscript that addresses the points raised during the review process.

==============================

Please include the following items when submitting your revised manuscript:

- A letter that responds to each point raised by the academic editor and reviewer(s). You should upload this letter as a separate file labeled 'Response to Reviewers'.
- A marked-up copy of your manuscript that highlights changes made to the original version. You should upload this as a separate file labeled 'Revised Manuscript with Track Changes'.
- An unmarked version of your revised paper without tracked changes. You should upload this as a separate file labeled 'Manuscript'.

**Journal Requirements:**

If the reviewer comments include a recommendation to cite specific previously published works, please review and evaluate these publications to determine whether they are relevant and should be cited. There is no requirement to cite these works unless the editor has indicated otherwise. 


[Note: HTML markup is below. Please do not edit.]

Reviewers' comments:

Reviewer's Responses to Questions

**Comments to the Author**

1. If the authors have adequately addressed your comments raised in a previous round of review and you feel that this manuscript is now acceptable for publication, you may indicate that here to bypass the “Comments to the Author” section, enter your conflict of interest statement in the “Confidential to Editor” section, and submit your "Accept" recommendation.

Reviewer #1: (No Response)

2. Is the manuscript technically sound, and do the data support the conclusions?

The manuscript must describe a technically sound piece of scientific research with data that supports the conclusions. Experiments must have been conducted rigorously, with appropriate controls, replication, and sample sizes. The conclusions must be drawn appropriately based on the data presented.

Reviewer #1: Partly

3. Has the statistical analysis been performed appropriately and rigorously?

Reviewer #1: No

4. Have the authors made all data underlying the findings in their manuscript fully available?

The [PLOS Data policy](http://track.editorialmanager.com/CL0/http://www.plosone.org/static/policies.action%23sharing/1/010f019eecf2836e-4c9d8252-32ed-431b-b05b-a780c878a740-000000/yIP9FPzHeccGoj4GELdn8aiwU0nU1nRnCZPG8iBmVvM=258) requires authors to make all data underlying the findings described in their manuscript fully available without restriction, with rare exception (please refer to the Data Availability Statement in the manuscript PDF file). The data should be provided as part of the manuscript or its supporting information, or deposited to a public repository. For example, in addition to summary statistics, the data points behind means, medians and variance measures should be available. If there are restrictions on publicly sharing data—e.g. participant privacy or use of data from a third party—those must be specified.

Reviewer #1: No

5. Is the manuscript presented in an intelligible fashion and written in standard English?

PLOS ONE does not copyedit accepted manuscripts, so the language in submitted articles must be clear, correct, and unambiguous. Any typographical or grammatical errors should be corrected at revision, so please note any specific errors here.

Reviewer #1: No

6. Review Comments to the Author

Please use the space provided to explain your answers to the questions above. You may also include additional comments for the author, including concerns about dual publication, research ethics, or publication ethics. (Please upload your review as an attachment if it exceeds 20,000 characters)

**Reviewer #1:** 1. The manuscript claims that the proposed diffusion-sparse coding framework significantly improves the translation of culturally loaded words; however, the contribution of each component (sparse coding, diffusion generation, time-step-aware gating, and ontology constraints) is not sufficiently isolated. Additional ablation studies are required to quantify the individual impact of each module on translation performance.
Reply: Thank you for your valuable suggestions on the component contribution differentiation. we have added ablation experiments and used paired bootstrap tests (10,000 resamplings) to statistically quantify the contributions of each component. The results show that sparse coding and diffusion generation contribute the most significantly (p<0.001), while gating and knowledge constraint contributions are robust (p<0.005). Correlation analysis has been added to Section 3; please see the revised draft for details.

2. The cultural ontology knowledge graph contains only 186 entities and 312 relations. The manuscript does not provide sufficient evidence that this ontology is comprehensive enough to support the broad claims regarding cultural adaptability and semantic preservation across diverse news domains.
Reply: In response to the issues you raised, we supplemented the second section with the selection process for ontology nodes, demonstrated the representativeness of the ontology, and added coverage statistics to provide data to prove that the current ontology is sufficient to support the conclusions within the scope of this study's evaluation. Finally, in the conclusion section, I pointed out that cross-domain migration requires expanding the ontology, while emphasizing the universality of the methodological framework itself.

3. The manually constructed cultural evaluation subset contains only 500 sentences covering four cultural categories. The limited size and category coverage raise concerns regarding the generalizability of the reported results to broader real-world translation scenarios.
Reply: Based on your feedback, we added a Bootstrap confidence interval analysis to Section 3 to verify that the 500 sentences possess sufficient statistical validity in a diagnostic testing scenario; and I added a cross-set consistency analysis to the analysis section of Table 8 to demonstrate that the conclusions do not depend on specific sample selection. Furthermore, the conclusions explicitly limit the generalization claim to high cultural load scenarios in English-Chinese news and point out the generality of the methodological framework and the boundaries of the evaluation conclusions.

4. The proposed Cultural Semantic Consistency Index (SCI) plays a central role in evaluating cultural fidelity, yet its validation remains limited. Additional evidence is needed to demonstrate that SCI reliably correlates with human judgments of cultural translation quality.
Reply: Based on your suggestion, we added a correlation verification experiment between SCI and human cultural fidelity assessment in the revised draft: 100 samples were stratified and randomly selected from the test set, and three independent bilingual experts were invited to conduct a blind assessment of the cultural information retention of the four types of model translations (ICC=0.876). The results showed that the Pearson correlation coefficient between SCI and CIR scores was r=0.914 (95%CI: [0.887, 0.936], p<0.001), confirming that SCI can reliably reflect human judgment on the quality of cultural translation.

5. The manuscript reports improvements in BLEU, METEOR, accuracy, recall, and SCI, but no statistical significance testing is provided. Statistical validation should be included to confirm that the observed performance gains are robust and not due to dataset-specific effects.
Reply: Based on your suggestion, we have added statistical tests and stability analysis to Section 3. We conducted statistical tests on the results of our model and the comparative models at each test index level. The results show that our model is significantly better than the three baseline models in all indices (p < 0.01).

6. The reproducibility of the study remains limited because the ontology resources, cultural evaluation subset, and associated annotation materials are not publicly available. The current “available upon request” approach restricts independent verification of the results.
Reply: Thank you for your concern about reproducibility. In Section 2.3, I have added a detailed process for ontology construction: (1) seed word extraction criteria based on dual screening of word frequency and translation error rate; (2) specific operational specifications for independent annotation and consistency judgment by three experts; (3) judgment rules for four types of relation edges; (4) training parameter settings for TransE embedding; (5) quality results verified by blind review by independent experts; so as to support the independent reproduction of ontology construction in different language pairs or domains based on unified steps and judgment criteria.

7. The experimental evaluation is restricted to English–Chinese news translation. Although multilingual applicability is discussed, no empirical evidence is provided to support the claimed transferability of the framework to other language pairs or cross-cultural translation settings.
Reply: Thank you for pointing out the errors. We have added a new section to the conclusion acknowledging that the current experiment is limited to English-Chinese news corpora, pointing out the language-independent design of the framework, and outlining specific paths for multilingual verification such as English-Japanese and English-Arabic. This limits the discussion of multilingual applicability to theoretical prospects and avoids making over-assertions on unverified scenarios.
Overall, the topic is relevant and the proposed framework is potentially valuable. However, substantial revisions are required to strengthen the experimental validation, reproducibility, statistical rigor, and evidence supporting the claimed cultural generalization capabilities.

Reply: Thank you for your affirmation of the relevance of this paper's theme and the potential value of its framework. In response to your concerns regarding experimental verification, reproducibility, statistical rigor, and cultural generalization ability, we have supplemented the revised draft by adding ablation experiments and paired bootstrap tests to quantify the contributions of each component and verify the robustness of the results; we have also added a five-step cultural ontology construction process and verified the correlation between SCI and human evaluation, enhancing the credibility of the cultural adaptability indicators; and in the conclusion, we have limited the empirical boundary of this study to the English-Chinese news scenario and explicitly stated multilingual applicability as a theoretical prospect.

7. PLOS authors have the option to publish the peer review history of their article ([what does this mean?](https://track.editorialmanager.com/CL0/https://journals.plos.org/plosone/s/editorial-and-peer-review-process%23loc-peer-review-history/1/010f019eecf2836e-4c9d8252-32ed-431b-b05b-a780c878a740-000000/aWuHh1ZfQR7PdG7na8iqrTlSqilnQvRDaPyDm4NKyYs=258)). If published, this will include your full peer review and any attached files.


If you choose “no”, your identity will remain anonymous but your review may still be made public.


**Do you want your identity to be public for this peer review?** For information about this choice, including consent withdrawal, please see our [Privacy Policy](https://track.editorialmanager.com/CL0/https://www.plos.org/privacy-policy/1/010f019eecf2836e-4c9d8252-32ed-431b-b05b-a780c878a740-000000/s6LOJqBlmvtcHCRfyBiYG99z0ncZdg04MFOS1NDbFZc=258).

Reviewer #1: **Yes:** Amjed abbas Ahmed
