# Supplementary material for: Combining a diffusion model with a sparse coding strategy to improve the machine translation accuracy of culturally loaded words in English news texts
Source: PLoS One. 2026 Aug 28;21(8):e0356146. doi: 10.1371/journal.pone.0356146 (PMC13524296; doi:10.1371/journal.pone.0356146)
Supplement: Supplementary file 4 [file pone.0356146.s006.docx]

While the reviewer has indicated that the authors have addressed the comments, the manuscript still contains two issues that must be resolved to comply with PLOS ONE requirements:

Incomplete equations and missing symbols: Several equations and mathematical expressions still appear to be missing symbols, possibly due to issues during PDF generation. Please ensure that the final PDF is complete and properly rendered. For example, in Table 1, the symbol in Gu appears to be missing. In addition, all equations should be explicitly cited and discussed in the text unless they represent standard formulations that are not part of the authors' original contribution.

Answer: According to the formula rendering and citation issues you pointed out, we have corrected the formula symbols in Table 1, added references to all formulas one by one, and clarified the specific role of key formulas in sparse coding, dynamic gating and cultural constraints, improving the readability of the article.

Code availability for reproducibility: The source code is necessary to fully reproduce the results presented in the paper. While the datasets are available and Section 3.1 provides a discussion of the methodology, these alone are insufficient to ensure reproducibility. The authors should make the code available or provide a clear justification consistent with the journal's reproducibility requirements.

Answer: Thank you for pointing out the importance of code usability. We highly value the reproducibility of our research and, in accordance with journal policy, have uploaded the source code as supplementary material.
