# Supplementary material for: Combining a diffusion model with a sparse coding strategy to improve the machine translation accuracy of culturally loaded words in English news texts
Source: PLoS One. 2026 Aug 28;21(8):e0356146. doi: 10.1371/journal.pone.0356146 (PMC13524296; doi:10.1371/journal.pone.0356146)
Supplement: Supplementary file 5 [file pone.0356146.s007.docx]

PONE-D-26-07189R4

Combining a diffusion model with a sparse coding strategy to improve the machine translation accuracy of culturally loaded words in English news texts

PLOS One

Dear Dr. Yu,

Thank you for submitting your manuscript to PLOS ONE. After careful consideration, we feel that it has merit but does not fully meet PLOS ONE’s publication criteria as it currently stands. Therefore, we invite you to submit a revised version of the manuscript that addresses the points raised during the review process.
Answer: We sincerely thank you and the reviewers for your positive feedback and valuable comments. We have revised the manuscript according to each reviewer's comment, paying particular attention to the display issues of formula symbols, correcting rendering errors in mathematical symbols and table variables, and generating a new PDF for verification. The revised manuscript has been uploaded in its entirety, and we kindly request your review. We hope the manuscript will meet PLOS ONE's publication standards. Thank you again for your support and guidance!

==============================

This is the third time that equation symbols have not rendered correctly. Please check the generated file before approval. The issue affects not only Table 1 but multiple equations throughout the manuscript. For example, in Table 1, the formula displays ℝ，□gu∈[0,1] with an empty square (□) before g, indicating a missing symbol. Please review all equations and mathematical symbols carefully.

Answer: We sincerely apologize for the issue you pointed out regarding the display of formula symbols. We have rectified the mathematical symbols with rendering errors: we specifically corrected "□gu" in Table 1 and checked all inline and cross-line formulas one by one to ensure that the symbols were rendered correctly. The revised version has been recompiled and a new PDF has been generated for verification. Thank you for your meticulous correction!

==============================

Please submit your revised manuscript by Aug 30 2026 11:59PM. If you will need more time than this to complete your revisions, please reply to this message or contact the journal office at [plosone@plos.org](mailto:plosone@plos.org). When you're ready to submit your revision, log on to https://www.editorialmanager.com/pone/ and select the 'Submissions Needing Revision' folder to locate your manuscript file.

Please include the following items when submitting your revised manuscript:

- A letter that responds to each point raised by the academic editor and reviewer(s). You should upload this letter as a separate file labeled 'Response to Reviewers'.
- A marked-up copy of your manuscript that highlights changes made to the original version. You should upload this as a separate file labeled 'Revised Manuscript with Track Changes'.
- An unmarked version of your revised paper without tracked changes. You should upload this as a separate file labeled 'Manuscript'.

If you would like to make changes to your financial disclosure, please include your updated statement in your cover letter. Guidelines for resubmitting your figure files are available below the reviewer comments at the end of this letter.
Answer: Thank you for providing the detailed revision submission guidelines. We have prepared all materials as required: 1) A separate response letter to each reviewer's comment, titled "Response to Reviewers"; 2) An uploaded revised manuscript with tracked changes, named "Revised Manuscript with Track Changes"; 3) An uploaded final manuscript without any markings, named "Manuscript". All figures and tables have been resubmitted according to the guidelines. These revisions address all reviewer comments; your review is appreciated.

If applicable, we recommend that you deposit your laboratory protocols in protocols.io to enhance the reproducibility of your results. Protocols.io assigns your protocol its own identifier (DOI) so that it can be cited independently in the future. For instructions see: [https://journals.plos.org/plosone/s/submission-guidelines#loc-laboratory-protocols](https://track.editorialmanager.com/CL0/https://journals.plos.org/plosone/s/submission-guidelines%23loc-laboratory-protocols/1/010f019f6e28719c-17bb2c76-fc5c-4f1f-8ea9-4339eb941f11-000000/XfTdKrpLayrBJ7eXxObCqR8J6wXIeuw36kyuYodNAlY=258). Additionally, PLOS ONE offers an option for publishing peer-reviewed Lab Protocol articles, which describe protocols hosted on protocols.io. Read more information on sharing protocols at [https://plos.org/protocols?utm_medium=editorial-email&utm_source=authorletters&utm_campaign=protocols](https://track.editorialmanager.com/CL0/https://plos.org/protocols?utm_medium=editorial-email&utm_source=authorletters&utm_campaign=protocols/1/010f019f6e28719c-17bb2c76-fc5c-4f1f-8ea9-4339eb941f11-000000/eCf_4qK4zvHgwtPnTFZtvuX7sS03P3O-8fqnePeWz0A=258).

Answer: Thank you for your suggestion regarding sharing the experimental protocol. We fully understand the protocols.io platform and its value in improving research reproducibility. Considering that this research primarily involves model architecture and algorithm implementation, and does not involve traditional wet testing procedures, we have not yet established a dedicated experimental protocol on protocols.io. However, we have described the model parameters, training process, and code implementation details in detail in the manuscript and methods sections, and will provide access to the core code in the data availability statement to ensure the reproducibility of the research results to the greatest extent possible. We are happy to cooperate if there are any further requests.

As the corresponding author, your ORCID iD is verified in the submission system and will appear in the published article. PLOS supports the use of ORCID, and we encourage all coauthors to register for an ORCID iD and use it as well. Please encourage your coauthors to verify their ORCID iD within the submission system before final acceptance, as unverified ORCID iDs will not appear in the published article. Only the individual author can complete the verification step; PLOS staff cannot verify ORCID iDs on behalf of authors.

Answer: Thank you for your reminder regarding ORCID verification. As the corresponding author, I verified my own ORCID in the system upon submission. I also notified all co-authors via email, urging them to log in to the submission system and verify their respective ORCIDs as soon as possible before final acceptance of the article, ensuring that all named authors' ORCIDs are displayed correctly upon publication. I will continue to follow up on this matter and will reconfirm the verification status of all authors before acceptance.

We look forward to receiving your revised manuscript.

Kind regards,

Issa Atoum
Academic Editor
PLOS One

Journal Requirements:

If the reviewer comments include a recommendation to cite specific previously published works, please review and evaluate these publications to determine whether they are relevant and should be cited. There is no requirement to cite these works unless the editor has indicated otherwise. 

Please review your reference list to ensure that it is complete and correct. If you have cited papers that have been retracted, please include the rationale for doing so in the manuscript text, or remove these references and replace them with relevant current references. Any changes to the reference list should be mentioned in the rebuttal letter that accompanies your revised manuscript. If you need to cite a retracted article, indicate the article’s retracted status in the References list and also include a citation and full reference for the retraction notice.
Answer: Thank you for your reminder regarding the references. We have reviewed the full-text reference list and confirmed that all cited references have not been retracted and are formatted correctly and completely. Any additions or deletions to the references were clearly indicated in the reply email. We will continue to monitor the status of the references to ensure the reliability of the cited content. Thank you for your meticulous review!

[Note: HTML markup is below. Please do not edit.]

[NOTE: If reviewer comments were submitted as an attachment file, they will be attached to this email and accessible via the submission site. Please log into your account, locate the manuscript record, and check for the action link "View Attachments". If this link does not appear, there are no attachment files.]

To ensure your figures meet our technical requirements, please review our figure guidelines: https://journals.plos.org/plosone/s/figures

You may also use PLOS’s free figure tool, NAAS, to help you prepare publication quality figures: https://journals.plos.org/plosone/s/figures#loc-tools-for-figure-preparation.

NAAS will assess whether your figures meet our technical requirements by comparing each figure against our figure specifications.

Answer: Thank you for providing the figures guide and the NAAS tool link. We have carefully reviewed the relevant technical requirements to ensure that the documents meet the specifications regarding format, resolution, size, and file size. The revised manuscript includes figures that meet publication quality requirements. If you encounter any technical issues, we will contact the journal support promptly. Thank you for your detailed guidance!

###### Copilot said:

This is the third time that equation symbols have not rendered correctly. Please check the generated file before approval. The issue affects not only Table 1 but multiple equations throughout the manuscript. For example, in Table 1, the formula displays ℝ， gu∈[0,1] with an empty square (□) before **g**, indicating a missing symbol. Please review all equations and mathematical symbols carefully.

Answer: We sincerely apologize for the issue you pointed out regarding the display of formula symbols. We have rectified the mathematical symbols with rendering errors: we specifically corrected "□gu" in Table 1 and checked all inline and cross-line formulas one by one to ensure that the symbols were rendered correctly. The revised version has been recompiled and a new PDF has been generated for verification. Thank you for your meticulous correction!
