# Supplementary material for: Combining a diffusion model with a sparse coding strategy to improve the machine translation accuracy of culturally loaded words in English news texts
Source: PLoS One. 2026 Aug 28;21(8):e0356146. doi: 10.1371/journal.pone.0356146 (PMC13524296; doi:10.1371/journal.pone.0356146)
Supplement: Supplementary file 6 [file pone.0356146.s008.docx]

ear Dr. Yu,

Thank you for submitting your manuscript to PLOS ONE. After careful consideration, we feel that it has merit but does not fully meet PLOS ONE’s publication criteria as it currently stands. Therefore, we invite you to submit a revised version of the manuscript that addresses the points raised during the review process.

==============================
Unfortunately, the equations are still not displayed correctly. Please try to use LaTeX. Moreover, the provided code is not fully reproducible because it is isolated from the datasets and corresponding outputs. If sharing the datasets is not possible, please provide executable notebooks that reproduce the reported results and demonstrate the complete analysis workflow.
==============================
Answer: Thank you for your valuable feedback. Regarding the formula display issue, after review, we found that some formula symbols in the original manuscript were mistakenly converted to plain text during the format conversion process, resulting in rendering distortion. Therefore, we have rewritten all formulas using standard LaTeX syntax and output files for verification. Error-free compiled PDF files are included as supplementary files in the folder. Regarding code and data reproduction, the WMT18 and TED corpora used in this study are publicly available resources. To ensure reproducibility, we have provided complete executable files in the supplementary materials, including preprocessing, model training, and evaluation procedures, and fixed random seeds and hyperparameters. Approximate test sets can be reconstructed from the publicly available corpora according to the instructions.

Please submit your revised manuscript by Sep 06 2026 11:59PM. If you will need more time than this to complete your revisions, please reply to this message or contact the journal office at [plosone@plos.org](mailto:plosone@plos.org). When you're ready to submit your revision, log on to https://www.editorialmanager.com/pone/ and select the 'Submissions Needing Revision' folder to locate your manuscript file.
Answer: Thank you for your letter and deadline reminder. We confirm that we will submit the revised manuscript through the system by 23:59 on September 6, 2026. Regarding the reviewers' comments, we have corrected all formulas to LaTeX format and provided detailed data construction instructions to ensure reproducibility. We will contact the editorial office promptly if necessary. Thank you again for your support!
Please include the following items when submitting your revised manuscript:

- A letter that responds to each point raised by the academic editor and reviewer(s). You should upload this letter as a separate file labeled 'Response to Reviewers'.
- A marked-up copy of your manuscript that highlights changes made to the original version. You should upload this as a separate file labeled 'Revised Manuscript with Track Changes'.
- An unmarked version of your revised paper without tracked changes. You should upload this as a separate file labeled 'Manuscript'.

If you would like to make changes to your financial disclosure, please include your updated statement in your cover letter. Guidelines for resubmitting your figure files are available below the reviewer comments at the end of this letter.
Answer: Thank you for your detailed instructions regarding the submission of the revised manuscript. We have prepared the following as required: a letter responding to each of the reviewers' comments, a revised manuscript with revision marks, and a final, unmarked manuscript. All documents were uploaded to the system before the deadline. Regarding the supplementary instructions on resubmitting financial disclosures and charts, we will carefully review each requirement to ensure that the materials are complete and compliant. Thank you again for your guidance!

If applicable, we recommend that you deposit your laboratory protocols in protocols.io to enhance the reproducibility of your results. Protocols.io assigns your protocol its own identifier (DOI) so that it can be cited independently in the future. For instructions see: [https://journals.plos.org/plosone/s/submission-guidelines#loc-laboratory-protocols](https://track.editorialmanager.com/CL0/https://journals.plos.org/plosone/s/submission-guidelines%23loc-laboratory-protocols/1/010f019f91fc325c-c0a29c38-5b68-4e48-8da2-5d59add92cbd-000000/6zhpMcLkBVdKlEPKGbJHpZu2ejgG-xz04FPwXXkkq7I=258). Additionally, PLOS ONE offers an option for publishing peer-reviewed Lab Protocol articles, which describe protocols hosted on protocols.io. Read more information on sharing protocols at [https://plos.org/protocols?utm_medium=editorial-email&utm_source=authorletters&utm_campaign=protocols](https://track.editorialmanager.com/CL0/https://plos.org/protocols?utm_medium=editorial-email&utm_source=authorletters&utm_campaign=protocols/1/010f019f91fc325c-c0a29c38-5b68-4e48-8da2-5d59add92cbd-000000/TcGMvurMwlLgbINrW_sDRJEQ5cFWy3GDuy7CCJ_fbNg=258).

Answer: Thank you for your suggestion regarding sharing the experimental protocol. We fully understand the protocols.io platform and its value in improving research reproducibility. Considering that this research primarily involves model architecture and algorithm implementation, and does not involve traditional wet testing procedures, we have not yet established a dedicated experimental protocol on protocols.io. However, we have described the model parameters, training process, and code implementation details in detail in the manuscript and methods sections, and will provide access to the core code in the data availability statement to ensure the reproducibility of the research results to the greatest extent possible. We are happy to cooperate if there are any further requests.

We look forward to receiving your revised manuscript.

Kind regards,

Issa Atoum
Academic Editor
PLOS One

Journal Requirements:

If the reviewer comments include a recommendation to cite specific previously published works, please review and evaluate these publications to determine whether they are relevant and should be cited. There is no requirement to cite these works unless the editor has indicated otherwise. 

Answer: Thank you for your reminder regarding the references. We have reviewed the full-text reference list and confirmed that all cited references have not been retracted and are formatted correctly and completely. Any additions or deletions to the references were clearly indicated in the reply email. We will continue to monitor the status of the references to ensure the reliability of the cited content. Thank you for your meticulous review!

[Note: HTML markup is below. Please do not edit.]


[NOTE: If reviewer comments were submitted as an attachment file, they will be attached to this email and accessible via the submission site. Please log into your account, locate the manuscript record, and check for the action link "View Attachments". If this link does not appear, there are no attachment files.]

To ensure your figures meet our technical requirements, please review our figure guidelines: https://journals.plos.org/plosone/s/figures

You may also use PLOS’s free figure tool, NAAS, to help you prepare publication quality figures: https://journals.plos.org/plosone/s/figures#loc-tools-for-figure-preparation.

NAAS will assess whether your figures meet our technical requirements by comparing each figure against our figure specifications.

Answer: Thank you for providing the figures guide and the NAAS tool link. We have carefully reviewed the relevant technical requirements to ensure that the documents meet the specifications regarding format, resolution, size, and file size. The revised manuscript includes figures that meet publication quality requirements. If you encounter any technical issues, we will contact the journal support promptly. Thank you for your detailed guidance!
