# Supplementary material for: Combining a diffusion model with a sparse coding strategy to improve the machine translation accuracy of culturally loaded words in English news texts
Source: PLoS One. 2026 Aug 28;21(8):e0356146. doi: 10.1371/journal.pone.0356146 (PMC13524296; doi:10.1371/journal.pone.0356146)
Supplement: Peer review history

# Peer review: Decision Letter 0

**DOI:** 10.1371/journal.pone.0356146.r001

**Authors:** Issa Atoum

8 Apr 2026

Dear Dr. Yu,

Thank you for submitting your manuscript to PLOS ONE. After careful consideration, we feel that it has merit but does not fully meet PLOS ONE’s publication criteria as it currently stands. Therefore, we invite you to submit a revised version of the manuscript that addresses the points raised during the review process.

==============================

While the reviewers agree that the topic is potentially interesting, the paper in its current form requires substantial revision before it can be considered further. The main concern is that the experimental evidence does not yet support the strength of the claims. The revised manuscript should report variability across repeated runs, confidence intervals, and appropriate significance testing for the reported results. It should also include clear ablation studies to separate the contribution of each major component, including sparse coding, the diffusion mechanism, and the gating module. In addition, the evaluation needs to be strengthened through broader validation, ideally with a larger or external dataset beyond the current 500-sentence manually constructed subset. The reviewers also identified important issues related to reproducibility, clarity, and presentation.

==============================

Please submit your revised manuscript by May 23 2026 11:59PM. If you will need more time than this to complete your revisions, please reply to this message or contact the journal office at plosone@plos.org. When you're ready to submit your revision, log on to https://www.editorialmanager.com/pone/ and select the 'Submissions Needing Revision' folder to locate your manuscript file.

- A letter that responds to each point raised by the academic editor and reviewer(s). You should upload this letter as a separate file labeled 'Response to Reviewers'.
- A marked-up copy of your manuscript that highlights changes made to the original version. You should upload this as a separate file labeled 'Revised Manuscript with Track Changes'.
- An unmarked version of your revised paper without tracked changes. You should upload this as a separate file labeled 'Manuscript'.

If you would like to make changes to your financial disclosure, please include your updated statement in your cover letter. Guidelines for resubmitting your figure files are available below the reviewer comments at the end of this letter.

If applicable, we recommend that you deposit your laboratory protocols in protocols.io to enhance the reproducibility of your results. Protocols.io assigns your protocol its own identifier (DOI) so that it can be cited independently in the future. For instructions see: https://journals.plos.org/plosone/s/submission-guidelines#loc-laboratory-protocols. Additionally, PLOS ONE offers an option for publishing peer-reviewed Lab Protocol articles, which describe protocols hosted on protocols.io. Read more information on sharing protocols at https://plos.org/protocols?utm_medium=editorial-email&utm_source=authorletters&utm_campaign=protocols.

As the corresponding author, your ORCID iD is verified in the submission system and will appear in the published article. PLOS supports the use of ORCID, and we encourage all coauthors to register for an ORCID iD and use it as well. Please encourage your coauthors to verify their ORCID iD within the submission system before final acceptance, as unverified ORCID iDs will not appear in the published article. *Only*  the individual author can complete the verification step; PLOS staff *cannot*  verify ORCID iDs on behalf of authors.

We look forward to receiving your revised manuscript.

Kind regards,

Issa Atoum

Academic Editor

PLOS One

Journal Requirements:

When submitting your revision, we need you to address these additional requirements.

1. Please ensure that your manuscript meets PLOS ONE’s style requirements, including those for file naming. The PLOS ONE style templates can be found at

https://journals.plos.org/plosone/s/file?id=wjVg/PLOSOne_formatting_sample_main_body.pdf and

https://journals.plos.org/plosone/s/file?id=ba62/PLOSOne_formatting_sample_title_authors_affiliations.pdf

2. Please note that PLOS One has specific guidelines on code sharing for submissions in which author-generated code underpins the findings in the manuscript. In these cases, we expect all author-generated code to be made available without restrictions upon publication of the work. Please review our guidelines at https://journals.plos.org/plosone/s/materials-and-software-sharing#loc-sharing-code and ensure that your code is shared in a way that follows best practice and facilitates reproducibility and reuse.

3. Please provide a complete Data Availability Statement in the submission form, ensuring you include all necessary access information or a reason for why you are unable to make your data freely accessible. If your research concerns only data provided within your submission, please write "All data are in the manuscript and/or supporting information files" as your Data Availability Statement.

4. PLOS requires an ORCID iD for the corresponding author in Editorial Manager on papers submitted after December 6th, 2016. Please ensure that you have an ORCID iD and that it is validated in Editorial Manager. To do this, go to ‘Update my Information’ (in the upper left-hand corner of the main menu), and click on the Fetch/Validate link next to the ORCID field. This will take you to the ORCID site and allow you to create a new iD or authenticate a pre-existing iD in Editorial Manager.

5. If the reviewer comments include a recommendation to cite specific previously published works, please review and evaluate these publications to determine whether they are relevant and should be cited. There is no requirement to cite these works unless the editor has indicated otherwise.

[Note: HTML markup is below. Please do not edit.]

Reviewers' comments:

Reviewer’s Responses to Questions

**Comments to the Author**

1. Is the manuscript technically sound, and do the data support the conclusions?

Reviewer #1: No

Reviewer #2: No

**********

2. Has the statistical analysis been performed appropriately and rigorously?

Reviewer #1: No

Reviewer #2: No

**********

3. Have the authors made all data underlying the findings in their manuscript fully available?

The PLOS Data policy

Reviewer #1: No

Reviewer #2: No

**********

4. Is the manuscript presented in an intelligible fashion and written in standard English?

Reviewer #1: No

Reviewer #2: No

**********

Reviewer #1: 1-Details of the proposed model in the manuscript are not sufficient to reproduce it. Important factors, such as the data splits, preprocessing pipeline, random seeds and training stability across multiple runs are absent. However, the lack of reproducibility documentation undermines the scientific validity of the work despite combining sparse coding, diffusion and knowledge constraints.

2-All reported results (BLEU, METEOR, TER, Accuracy, Recall, HTER SCI) are show as single values with no variance, confidence intervals or statistical tests. There was no evidence that the respective improvements over Transformer, mBART-50 and DiffuSeq were statistically significant which casts doubt on the veracity of their conclusions.

3-The manuscript integrates several complex components (sparse coding + diffusion decoding + cultural knowledge graph + gating mechanism), without any ablation studies that could isolate the effects of individual modules. Thus it is not obvious whether the performance benefits are due to the full architecture or just certain components of it.

4-The details regarding annotation methodology, inter-annotator agreement or bias control are absent in the manually constructed test subset (500 sentences). This raises potential for low reliability of evaluation between both people and cultures, especially genuine and subjective words.

5-Access to the processed datasets, annotated subset and intermediate data in experiments is not provided. The Data Availability statement restricts access without appropriate reasons given, which is in conflict with open science practices and PLOS data-sharing policies.

6-The maths are not clearly or consistently defined -- a number of the equations and model components (e.g., sparse coding formulation, gating mechanism, diffusion transition) are revealed but without in instructions that are sufficient. (Notation is sometimes ambiguous and dimensional consistency not always apparent, which hinders full comprehension/imposed of the method.)

7-Despite being pitched as an important advance the manuscript itself fails to motivate how their contribution is new beyond an amalgamation of techniques (Transformer, sparse coding diffusion models knowledge graph). The novelty is not well enough defined, and claims need to be toned down or better justified.

Reviewer #2: The manuscript proposes a hybrid framework combining diffusion models, sparse coding, and cultural ontology constraints to improve machine translation of culturally loaded words in English news texts. The topic is relevant and the integration of multiple techniques is interesting. However, several methodological and presentation issues limit the current strength of the work.

First, while the proposed architecture is technically elaborate, the manuscript lacks sufficient experimental rigor to support the claimed improvements. Although the results indicate performance gains over baseline models (e.g., Transformer, mBART-50, DiffuSeq), the improvements are relatively moderate and are not supported by statistical significance testing. The manuscript does not report confidence intervals, variance across runs, or results from multiple random seeds, which makes it difficult to assess the robustness of the findings.

Second, the study does not include ablation experiments to isolate the contributions of the individual components (sparse coding, diffusion mechanism, and cultural knowledge constraints). Without such analysis, it is unclear which component is primarily responsible for the observed performance gains.

Third, the evaluation dataset is limited, particularly the manually constructed test subset of 500 sentences. While this dataset is useful for focused evaluation of culturally loaded words, its size may not be sufficient to support strong general conclusions. Additional large-scale or external validation datasets would strengthen the study.

Fourth, the manuscript lacks clarity in methodological explanation. While the mathematical formulation is detailed, several components (e.g., gating mechanism, knowledge constraint integration) are not explained intuitively, making it difficult for readers to fully understand the model design and its practical implications.

Fifth, the data availability statement indicates restrictions, which limits reproducibility. The authors are encouraged to provide clearer information regarding dataset access, preprocessing steps, and implementation details to facilitate replication.

Finally, the manuscript requires substantial language editing. There are multiple grammatical issues, repetitive phrasing, and sections that are difficult to follow, which reduce readability.

In summary, while the proposed approach is conceptually interesting and has potential, the manuscript requires significant improvements in experimental validation, clarity, and reproducibility before it can be considered for publication.

**********

what does this mean?). If published, this will include your full peer review and any attached files.

If you choose “no”, your identity will remain anonymous but your review may still be made public.

**Do you want your identity to be public for this peer review?**  For information about this choice, including consent withdrawal, please see our Privacy Policy

Reviewer #1: **Yes:** AMJED ABBAS AHMED

Reviewer #2: No

**********

[NOTE: If reviewer comments were submitted as an attachment file, they will be attached to this email and accessible via the submission site. Please log into your account, locate the manuscript record, and check for the action link "View Attachments". If this link does not appear, there are no attachment files.]

To ensure your figures meet our technical requirements, please review our figure guidelines: https://journals.plos.org/plosone/s/figures

You may also use PLOS’s free figure tool, NAAS, to help you prepare publication quality figures: https://journals.plos.org/plosone/s/figures#loc-tools-for-figure-preparation.

NAAS will assess whether your figures meet our technical requirements by comparing each figure against our figure specifications.

# Author comment: Author response to Decision Letter 1

**DOI:** 10.1371/journal.pone.0356146.r002

14 Apr 2026

Journal Requirements:

When submitting your revision, we need you to address these additional requirements.

1. Please ensure that your manuscript meets PLOS ONE’s style requirements, including those for file naming. The PLOS ONE style templates can be found at

https://journals.plos.org/plosone/s/file?id=wjVg/PLOSOne_formatting_sample_main_body.pdf and

https://journals.plos.org/plosone/s/file?id=ba62/PLOSOne_formatting_sample_title_authors_affiliations.pdf

Reply: Thank you for your guidance. We have revised the document item by item according to the PLOS ONE template, and adjusted the headings of the abstract, introduction, methods, results, and conclusions; the citation format of the references has been standardized.

2. Please note that PLOS One has specific guidelines on code sharing for submissions in which author-generated code underpins the findings in the manuscript. In these cases, we expect all author-generated code to be made available without restrictions upon publication of the work. Please review our guidelines at https://journals.plos.org/plosone/s/materials-and-software-sharing#loc-sharing-code and ensure that your code is shared in a way that follows best practice and facilitates reproducibility and reuse.

Reply: Thank you for reminding us of PLOS ONE’s code-sharing policy. We have carefully read the relevant guidelines. The self-written code used for model training, evaluation, and analysis in this study, along with its algorithmic logic and key parameters, are fully reported in Chapters 2 and 3 of the manuscript, in the Methods and Experimental Setup sections, to support the reproduction of the findings.

3. Please provide a complete Data Availability Statement in the submission form, ensuring you include all necessary access information or a reason for why you are unable to make your data freely accessible. If your research concerns only data provided within your submission, please write "All data are in the manuscript and/or supporting information files" as your Data Availability Statement.

Reply: Thank you for your reminder. In accordance with the PLOS ONE data availability policy, we have added a complete data availability statement to the manuscript: WMT18 News Commentary v13 and TED Talks Parallel Corpus used in this study are publicly available bilingual corpora, accessible through their official websites.

4.PLOS requires an ORCID iD for the corresponding author in Editorial Manager on papers submitted after December 6th, 2016. Please ensure that you have an ORCID iD and that it is validated in Editorial Manager. To do this, go to ‘Update my Information’ (in the upper left-hand corner of the main menu), and click on the Fetch/Validate link next to the ORCID field. This will take you to the ORCID site and allow you to create a new iD or authenticate a pre-existing iD in Editorial Manager.

Reply: Thank you for your reminder. I have updated my Information in the main menu.

5. If the reviewer comments include a recommendation to cite specific previously published works, please review and evaluate these publications to determine whether they are relevant and should be cited. There is no requirement to cite these works unless the editor has indicated otherwise.

Reply: Thank you for your valuable suggestions. We have carefully reviewed the cited literature and assessed its relevance to our core contribution of combining Diffusion models with sparse coding to improve the translation of culture-loaded words, without blindly adding irrelevant citations.

Reviewers' comments:

Reviewer’s Responses to Questions

Comments to the Author

1. Is the manuscript technically sound, and do the data support the conclusions?

The manuscript must describe a technically sound piece of scientific research with data that supports the conclusions. Experiments must have been conducted rigorously, with appropriate controls, replication, and sample sizes. The conclusions must be drawn appropriately based on the data presented.

Reviewer #1: No

Reviewer #2: No

2. Has the statistical analysis been performed appropriately and rigorously?

Reviewer #1: No

Reviewer #2: No

3. Have the authors made all data underlying the findings in their manuscript fully available?

The PLOS Data policy requires authors to make all data underlying the findings described in their manuscript fully available without restriction, with rare exception (please refer to the Data Availability Statement in the manuscript PDF file). The data should be provided as part of the manuscript or its supporting information, or deposited to a public repository. For example, in addition to summary statistics, the data points behind means, medians and variance measures should be available. If there are restrictions on publicly sharing data—e.g. participant privacy or use of data from a third party—those must be specified.

Reviewer #1: No

Reviewer #2: No

4. Is the manuscript presented in an intelligible fashion and written in standard English?

PLOS ONE does not copyedit accepted manuscripts, so the language in submitted articles must be clear, correct, and unambiguous. Any typographical or grammatical errors should be corrected at revision, so please note any specific errors here.

Reviewer #1: No

Reviewer #2: No

5. Review Comments to the Author

Please use the space provided to explain your answers to the questions above. You may also include additional comments for the author, including concerns about dual publication, research ethics, or publication ethics. (Please upload your review as an attachment if it exceeds 20,000 characters)

Reviewer #1: 1-Details of the proposed model in the manuscript are not sufficient to reproduce it. Important factors, such as the data splits, preprocessing pipeline, random seeds and training stability across multiple runs are absent. However, the lack of reproducibility documentation undermines the scientific validity of the work despite combining sparse coding, diffusion and knowledge constraints.

Reply: Thank you for your suggestions. We have revised the manuscript accordingly: in Section 3.2, “Experimental Setup,” we have added details on data partitioning and preprocessing, stratified sampling strategy, and a random seed list (42,123,2024,5678,9999), which improves the scientific validity and transparency of the study.

2-All reported results (BLEU, METEOR, TER, Accuracy, Recall, HTER SCI) are show as single values with no variance, confidence intervals or statistical tests. There was no evidence that the respective improvements over Transformer, mBART-50 and DiffuSeq were statistically significant which casts doubt on the veracity of their conclusions.

Reply: Based on your suggestion, we have revised Section 3.3, adding Section 3.3.4 for statistical significance testing and stability analysis, which summarizes the statistical results of each indicator. All improvements are statistically significant (p<0.01) and have small standard deviations, proving that the model is stable and reliable.

3-The manuscript integrates several complex components (sparse coding + diffusion decoding + cultural knowledge graph + gating mechanism), without any ablation studies that could isolate the effects of individual modules. Thus it is not obvious whether the performance benefits are due to the full architecture or just certain components of it.

Reply: Thank you for pointing out the necessity of the ablation experiments. We have added "ablation experiments" as suggested, designing five variants to remove sparse coding, diffusion decoding, knowledge graph constraints, and gating mechanisms, respectively. The results show that the complete model outperforms any ablation variant on all metrics, especially in BLEU-4 and OOV accuracy, validating the overall effectiveness of our proposed architecture.

4-The details regarding annotation methodology, inter-annotator agreement or bias control are absent in the manually constructed test subset (500 sentences). This raises potential for low reliability of evaluation between both people and cultures, especially genuine and subjective words.

Reply: In response to your question, we have supplemented Section 3.1 with the process for constructing and annotating the test subset: including independent annotation by three bilingual experts and Fleiss’s K consistency, which improves the transparency of data construction and the reliability of evaluation results, and ensures the effectiveness and reproducibility of the test subset in different cultural contexts.

5-Access to the processed datasets, annotated subset and intermediate data in experiments is not provided. The Data Availability statement restricts access without appropriate reasons given, which is in conflict with open science practices and PLOS data-sharing policies.

Reply: Thank you for your feedback. We have revised the "Data Availability Statement": It now clarifies that the WMT18 and TED corpora used in this study are all publicly available resources; the construction method and annotation process of the dedicated test subset have been fully described in Section 3.1; all preprocessing, word segmentation, data partitioning, and other parameters are detailed in Section 3.2, and you can reproduce all the results yourself by following the detailed steps provided in the text.

6-The maths are not clearly or consistently defined -- a number of the equations and model components (e.g., sparse coding formulation, gating mechanism, diffusion transition) are revealed but without in instructions that are sufficient. (Notation is sometimes ambiguous and dimensional consistency not always apparent, which hinders full comprehension/imposed of the method.)

Reply: Thank you for pointing out the shortcomings in the formula description. We have added "Symbol Explanation" to Chapter 2 as suggested, unifying all variable symbols, dimensions, and value ranges; added dimension annotations and parameter explanations to the key formulas for sparse coding, gating mechanisms, and diffusion transfer; and corrected any ambiguities and inconsistencies in the symbols.

7-Despite being pitched as an important advance the manuscript itself fails to motivate how their contribution is new beyond an amalgamation of techniques (Transformer, sparse coding diffusion models knowledge graph). The novelty is not well enough defined, and claims need to be toned down or better justified.

Reply: Thank you for pointing out the lack of innovativeness in our description. We have clearly identified three core innovations at the end of the introduction: conditional coupling of sparse semantic anchors and diffusion trajectories, time-step-aware dynamic gating, and augmented Lagrangian soft-constraint knowledge alignment, and have demonstrated the synergistic effect of these three elements. At the same time, we have downplayed terms such as "combination" to highlight the originality of the mechanism and distinguish our contributions from existing work.

Reviewer #2: The manuscript proposes a hybrid framework combining diffusion models, sparse coding, and cultural ontology constraints to improve machine translation of culturally loaded words in English news texts. The topic is relevant and the integration of multiple techniques is interesting. However, several methodological and presentation issues limit the current strength of the work.

First, while the proposed architecture is technically elaborate, the manuscript lacks sufficient experimental rigor to support the claimed improvements. Although the results indicate performance gains over baseline models (e.g., Transformer, mBART-50, DiffuSeq), the improvements are relatively moderate and are not supported by statistical significance testing. The manuscript does not report confidence intervals, variance across runs, or results from multiple random seeds, which makes it difficult to assess the robustness of the findings.

Reply: Regarding your point about insufficient statistical rigor, we have supplemented Section 3.3 with the mean ± standard deviation of the main indicators and the results of statistical tests, using paired bootstrap tests to calculate p-values. The results show that the improvement of our model is statistically significant, and the standard deviation is small, validating the robustness of the results.

Second, the study does not include ablation experiments to isolate the contributions of the individual components (sparse coding, diffusion mechanism, and cultural knowledge constraints). Without such analysis, it is unclear which component is primarily responsible for the observed performance gains.

Reply: Thank you for pointing out the necessity of the ablation experiments. We have added Section 3.3, "Ablation Experiments," and designed five variants to remove sparse coding, diffusion decoding, knowledge graph constraints, and gating mechanisms, respectively. The results show that the complete model outperforms any ablation variant on all metrics, with each component making a key contribution, clearly separating and quantifying the role of each module.

Third, the evaluation dataset is limited, particularly the manually constructed test subset of 500 sentences. While this dataset is useful for focused evaluation of culturally loaded words, its size may not be sufficient to support strong general conclusions. Additional large-scale or external validation datasets would strengthen the study.

Reply: Based on your suggestion, we have added external validation analysis to Section 3.1. The results show that our model achieves BLEU-4 accuracy of 27.2% ± 0.4%, significantly better than the baseline (p < 0.01), consistent with the conclusions of the main test set. Furthermore, the limitations of the dataset and future expansion directions are discussed in the conclusions, enhancing the reliability of the findings.

Fourth, the manuscript lacks clarity in methodological explanation. While the mathematical formulation is detailed, several components (e.g., gating mechanism, knowledge constraint integration) are not explained intuitively, making it difficult for readers to fully understand the model design and its practical implications.

Reply: Based on your suggestion, we have used natural language to explain the collaborative logic of sparse anchor extraction, cultural condition construction, diffusion denoising and gating mechanism, and knowledge constraint calibration step by step at the beginning of Section 2, which improves the understandability of the model design and the transparency of practical applications.

Fifth, the data availability statement indicates restrictions, which limits reproducibility. The authors are encouraged to provide clearer information regarding dataset access, preprocessing steps, and implementation details to facilitate replication.

Reply: Based on your suggestion, we have detailed the dataset source, BPE segmentation, cleaning rule preprocessing steps, and implementation parameters such as learning rate, batch size, and random seed in Sections 3.1 and 3.2, and added a data availability declaration to clearly indicate the method description and ensure reproducibility.

Finally, the manuscript requires substantial language editing. There are multiple grammatical issues, repetitive phrasing, and sections that are difficult to follow, which reduce readability.

Reply: Based on your suggestions, we have polished the entire text: correcting grammatical errors and repetitive expressions, and improving readability and professionalism. Thank you again for your meticulous corrections.

In summary, while the proposed approach is conceptually interesting and has potential, the manuscript requires significant improvements in experimental validation, clarity, and reproducibility before it can be considered for publication.

Reply: Thank you for your overall evaluation and constructive criticism. We have made improvements in three areas: experimental validation, clarity, and reproducibility. We have added statistical significance tests, ablation experiments, and external validation sets, and improved the data availability statement and formula symbol explanations.

Attachment Submitted filename: Response to Reviewers.docx

# Peer review: Decision Letter 1

**DOI:** 10.1371/journal.pone.0356146.r003

**Authors:** Issa Atoum

1 Jun 2026

Dear Dr. Yu,

Thank you for submitting your manuscript to PLOS ONE. After careful consideration, we feel that it has merit but does not fully meet PLOS ONE’s publication criteria as it currently stands. Therefore, we invite you to submit a revised version of the manuscript that addresses the points raised during the review process.

Please submit your revised manuscript by Jul 16 2026 11:59PM. If you will need more time than this to complete your revisions, please reply to this message or contact the journal office at plosone@plos.org. When you're ready to submit your revision, log on to https://www.editorialmanager.com/pone/ and select the 'Submissions Needing Revision' folder to locate your manuscript file.

- A letter that responds to each point raised by the academic editor and reviewer(s). You should upload this letter as a separate file labeled 'Response to Reviewers'.
- A marked-up copy of your manuscript that highlights changes made to the original version. You should upload this as a separate file labeled 'Revised Manuscript with Track Changes'.
- An unmarked version of your revised paper without tracked changes. You should upload this as a separate file labeled 'Manuscript'.

If you would like to make changes to your financial disclosure, please include your updated statement in your cover letter. Guidelines for resubmitting your figure files are available below the reviewer comments at the end of this letter.

If applicable, we recommend that you deposit your laboratory protocols in protocols.io to enhance the reproducibility of your results. Protocols.io assigns your protocol its own identifier (DOI) so that it can be cited independently in the future. For instructions see: https://journals.plos.org/plosone/s/submission-guidelines#loc-laboratory-protocols. Additionally, PLOS ONE offers an option for publishing peer-reviewed Lab Protocol articles, which describe protocols hosted on protocols.io. Read more information on sharing protocols at https://plos.org/protocols?utm_medium=editorial-email&utm_source=authorletters&utm_campaign=protocols.

As the corresponding author, your ORCID iD is verified in the submission system and will appear in the published article. PLOS supports the use of ORCID, and we encourage all coauthors to register for an ORCID iD and use it as well. Please encourage your coauthors to verify their ORCID iD within the submission system before final acceptance, as unverified ORCID iDs will not appear in the published article. *Only*  the individual author can complete the verification step; PLOS staff *cannot*  verify ORCID iDs on behalf of authors.

We look forward to receiving your revised manuscript.

Kind regards,

Issa Atoum

Academic Editor

PLOS One

Journal Requirements:

If the reviewer comments include a recommendation to cite specific previously published works, please review and evaluate these publications to determine whether they are relevant and should be cited. There is no requirement to cite these works unless the editor has indicated otherwise.

Please review your reference list to ensure that it is complete and correct. If you have cited papers that have been retracted, please include the rationale for doing so in the manuscript text, or remove these references and replace them with relevant current references. Any changes to the reference list should be mentioned in the rebuttal letter that accompanies your revised manuscript. If you need to cite a retracted article, indicate the article’s retracted status in the References list and also include a citation and full reference for the retraction notice.

**Additional Editor Comments:**

Kindly address the new comments regarding clarification and manuscript presentation. In addition, please ensure that all raw and source data are deposited in a publicly accessible repository, in accordance with standard data availability requirements.

[Note: HTML markup is below. Please do not edit.]

Reviewers' comments:

Reviewer’s Responses to Questions

**Comments to the Author**

Reviewer #1: All comments have been addressed

**********

2. Is the manuscript technically sound, and do the data support the conclusions?

Reviewer #1: Yes

**********

3. Has the statistical analysis been performed appropriately and rigorously?

Reviewer #1: Yes

**********

4. Have the authors made all data underlying the findings in their manuscript fully available?

The PLOS Data policy

Reviewer #1: Yes

**********

5. Is the manuscript presented in an intelligible fashion and written in standard English?

Reviewer #1: Yes

**********

Reviewer #1: 1-Please provide a brief summary table describing the cultural ontology knowledge graph, including the number of entities, relation types, and major cultural categories represented in the ontology.

2-The discussion of the Cultural Semantic Consistency Index (SCI) can be strengthened by briefly explaining how SCI complements traditional metrics such as BLEU and METEOR in evaluating cultural translation quality.

3-Please include a short discussion on the computational overhead introduced by ontology constraints during inference, even if only in qualitative terms, to improve understanding of practical deployment considerations.

4-The description of the manually constructed cultural evaluation subset would benefit from additional details regarding the distribution of samples across the four cultural categories and the rationale for category selection.

5-Please clarify whether the ontology resources and cultural evaluation subset will be made publicly available after publication to facilitate future research and reproducibility.

6-The future research section could be improved by briefly discussing multilingual extension possibilities, particularly the applicability of the proposed framework to language pairs beyond English–Chinese translation.

**********

what does this mean?). If published, this will include your full peer review and any attached files.

If you choose “no”, your identity will remain anonymous but your review may still be made public.

**Do you want your identity to be public for this peer review?**  For information about this choice, including consent withdrawal, please see our Privacy Policy

Reviewer #1: **Yes:** Amjed Abbas Ahmed

**********

[NOTE: If reviewer comments were submitted as an attachment file, they will be attached to this email and accessible via the submission site. Please log into your account, locate the manuscript record, and check for the action link "View Attachments". If this link does not appear, there are no attachment files.]

To ensure your figures meet our technical requirements, please review our figure guidelines: https://journals.plos.org/plosone/s/figures

You may also use PLOS’s free figure tool, NAAS, to help you prepare publication quality figures: https://journals.plos.org/plosone/s/figures#loc-tools-for-figure-preparation.

NAAS will assess whether your figures meet our technical requirements by comparing each figure against our figure specifications.

# Author comment: Author response to Decision Letter 2

**DOI:** 10.1371/journal.pone.0356146.r004

4 Jun 2026

Review Comments to the Author

Please use the space provided to explain your answers to the questions above. You may also include additional comments for the author, including concerns about dual publication, research ethics, or publication ethics. (Please upload your review as an attachment if it exceeds 20,000 characters)

Reviewer #1: 1-Please provide a brief summary table describing the cultural ontology knowledge graph, including the number of entities, relation types, and major cultural categories represented in the ontology.

Reply: Thank you for your suggestion. We have added Table 2 to Section 3.3, which briefly summarizes the cultural ontology knowledge graph. This ontology is constructed based on the cultural load words that actually appeared in the test subset of this experiment. It contains 186 entity nodes and 312 relation edges, covering four types of relations: equivalence, hierarchical, association, and contrast. It involves cultural categories such as religion, politics, festivals and social movements.

2-The discussion of the Cultural Semantic Consistency Index (SCI) can be strengthened by briefly explaining how SCI complements traditional metrics such as BLEU and METEOR in evaluating cultural translation quality.

Reply: Based on your suggestion, we have supplemented Section 4.3 with an explanation of the complementary relationship between the Cultural Semantic Consistency Index (SCI) and traditional metrics BLEU and METEOR. BLEU and METEOR focus on lexical and phrase-level matching, making it difficult to quantify the quality of cultural connotation delivery; while SCI, through a multilingual semantic embedding model, directly measures the alignment of the translation within the cultural knowledge space, specifically assessing cultural fidelity. Combining these three approaches provides a more comprehensive characterization of the model’s overall performance in cross-cultural translation tasks.

3-Please include a short discussion on the computational overhead introduced by ontology constraints during inference, even if only in qualitative terms, to improve understanding of practical deployment considerations.

Reply: Thank you for your suggestion. We have added a qualitative discussion regarding the computational overhead of ontology-constrained inference in the last section of the experimental part. Knowledge constraints are only activated in the last 200 denoising steps, and each computation is a dot product operation of a 30-dimensional vector, with minimal overhead; reordering only applies to 5 candidate paths. Compared to the iterative denoising overhead of the diffusion model itself, the knowledge constraint part accounts for approximately 8%~12% of the overall inference time, which is within an acceptable range. In practical deployments, further optimization can be achieved by reducing the number of candidate paths or selectively enabling constraints.

4-The description of the manually constructed cultural evaluation subset would benefit from additional details regarding the distribution of samples across the four cultural categories and the rationale for category selection.

Reply: Based on your suggestion, we have supplemented Section 3.1 with details of the cultural category distribution of the test subset. The selection of the four categories—religion, politics, festivals, and social movements—is based on their representativeness in high-frequency English news texts, the strength of their cultural specificity, and the current situation where cultural information is easily lost in machine translation. The religious category focuses on spiritual culture, the political category on institutional culture, the festival category on customary culture, and the social movement category on contemporary cultural phenomena.

5-Please clarify whether the ontology resources and cultural evaluation subset will be made publicly available after publication to facilitate future research and reproducibility.

Reply: Thank you for your attention. Regarding the accessibility of ontology resources and cultural evaluation subsets, due to the involvement of multiple parties and usage agreements, no publicly available channels have been established for their release. To meet the needs of researchers seeking to reproduce and extend these resources, we have detailed the ontology construction process, entity and relation definitions, annotation standards, and sampling strategies in our paper. Researchers requiring further information or to request access can contact us via the corresponding author’s email address; we will provide assistance within the scope permitted by the cooperation agreement. This section has been supplemented in the data availability statement.

6-The future research section could be improved by briefly discussing multilingual extension possibilities, particularly the applicability of the proposed framework to language pairs beyond English–Chinese translation.

Reply: Following your guidance, we have supplemented the conclusion with a discussion on multilingual extensions. Specifically, the proposed sparse coding and diffusion generation synergistic mechanism is language-independent: the sparse coding module does not rely on specific language priors, and the diffusion decoder can be extended by constructing multilingual cultural ontologies. This framework can be directly transferred to other language pairs such as English-Japanese and English-Arabic. For low-resource language pairs, cross-lingual pre-trained models can be used for initialization. We also emphasize the need for further adaptation for language pairs with complex morphology or large cultural distances, and related research will be an important direction for future work.

Attachment Submitted filename: Response_to_Reviewers_auresp_2.docx

# Peer review: Decision Letter 2

**DOI:** 10.1371/journal.pone.0356146.r005

**Authors:** Issa Atoum

21 Jun 2026

Dear Dr. Yu,

Thank you for submitting your manuscript to PLOS ONE. After careful consideration, we feel that it has merit but does not fully meet PLOS ONE’s publication criteria as it currently stands. Therefore, we invite you to submit a revised version of the manuscript that addresses the points raised during the review process.

==============================

While the manuscript shows some progress, the reviewer raises significant concerns regarding the adequacy of experimental validation and the lack of rigorous analysis, particularly with respect to limited ablation studies and insufficient statistical support. The comments also question the generalizability of the findings, given the small dataset, the restricted scale of the cultural ontology, and the focus solely on English–Chinese evaluation.

Please also ensure that all equations are properly formatted, as several are not rendering correctly in the PDF. Carefully review all symbols to fix any instances where they appear blank or incorrectly displayed.

==============================

Please submit your revised manuscript by Aug 05 2026 11:59PM. If you will need more time than this to complete your revisions, please reply to this message or contact the journal office at plosone@plos.org. When you're ready to submit your revision, log on to https://www.editorialmanager.com/pone/ and select the 'Submissions Needing Revision' folder to locate your manuscript file.

- A letter that responds to each point raised by the academic editor and reviewer(s). You should upload this letter as a separate file labeled 'Response to Reviewers'.
- A marked-up copy of your manuscript that highlights changes made to the original version. You should upload this as a separate file labeled 'Revised Manuscript with Track Changes'.
- An unmarked version of your revised paper without tracked changes. You should upload this as a separate file labeled 'Manuscript'.

If you would like to make changes to your financial disclosure, please include your updated statement in your cover letter. Guidelines for resubmitting your figure files are available below the reviewer comments at the end of this letter.

If applicable, we recommend that you deposit your laboratory protocols in protocols.io to enhance the reproducibility of your results. Protocols.io assigns your protocol its own identifier (DOI) so that it can be cited independently in the future. For instructions see: https://journals.plos.org/plosone/s/submission-guidelines#loc-laboratory-protocols. Additionally, PLOS ONE offers an option for publishing peer-reviewed Lab Protocol articles, which describe protocols hosted on protocols.io. Read more information on sharing protocols at https://plos.org/protocols?utm_medium=editorial-email&utm_source=authorletters&utm_campaign=protocols.

As the corresponding author, your ORCID iD is verified in the submission system and will appear in the published article. PLOS supports the use of ORCID, and we encourage all coauthors to register for an ORCID iD and use it as well. Please encourage your coauthors to verify their ORCID iD within the submission system before final acceptance, as unverified ORCID iDs will not appear in the published article. *Only*  the individual author can complete the verification step; PLOS staff *cannot*  verify ORCID iDs on behalf of authors.

We look forward to receiving your revised manuscript.

Kind regards,

Issa Atoum

Academic Editor

PLOS One

**Journal Requirements:**

If the reviewer comments include a recommendation to cite specific previously published works, please review and evaluate these publications to determine whether they are relevant and should be cited. There is no requirement to cite these works unless the editor has indicated otherwise.

[Note: HTML markup is below. Please do not edit.]

Reviewers' comments:

Reviewer’s Responses to Questions

**Comments to the Author**

Reviewer #1: (No Response)

**********

2. Is the manuscript technically sound, and do the data support the conclusions?

Reviewer #1: Partly

**********

3. Has the statistical analysis been performed appropriately and rigorously?

Reviewer #1: No

**********

4. Have the authors made all data underlying the findings in their manuscript fully available?

The PLOS Data policy

Reviewer #1: No

**********

5. Is the manuscript presented in an intelligible fashion and written in standard English?

Reviewer #1: No

**********

**Reviewer #1:** 1. The manuscript claims that the proposed diffusion-sparse coding framework significantly improves the translation of culturally loaded words; however, the contribution of each component (sparse coding, diffusion generation, time-step-aware gating, and ontology constraints) is not sufficiently isolated. Additional ablation studies are required to quantify the individual impact of each module on translation performance.

2. The cultural ontology knowledge graph contains only 186 entities and 312 relations. The manuscript does not provide sufficient evidence that this ontology is comprehensive enough to support the broad claims regarding cultural adaptability and semantic preservation across diverse news domains.

3. The manually constructed cultural evaluation subset contains only 500 sentences covering four cultural categories. The limited size and category coverage raise concerns regarding the generalizability of the reported results to broader real-world translation scenarios.

4. The proposed Cultural Semantic Consistency Index (SCI) plays a central role in evaluating cultural fidelity, yet its validation remains limited. Additional evidence is needed to demonstrate that SCI reliably correlates with human judgments of cultural translation quality.

5. The manuscript reports improvements in BLEU, METEOR, accuracy, recall, and SCI, but no statistical significance testing is provided. Statistical validation should be included to confirm that the observed performance gains are robust and not due to dataset-specific effects.

6. The reproducibility of the study remains limited because the ontology resources, cultural evaluation subset, and associated annotation materials are not publicly available. The current “available upon request” approach restricts independent verification of the results.

7. The experimental evaluation is restricted to English–Chinese news translation. Although multilingual applicability is discussed, no empirical evidence is provided to support the claimed transferability of the framework to other language pairs or cross-cultural translation settings.

Overall, the topic is relevant and the proposed framework is potentially valuable. However, substantial revisions are required to strengthen the experimental validation, reproducibility, statistical rigor, and evidence supporting the claimed cultural generalization capabilities.

**********

what does this mean?). If published, this will include your full peer review and any attached files.

If you choose “no”, your identity will remain anonymous but your review may still be made public.

**Do you want your identity to be public for this peer review?**  For information about this choice, including consent withdrawal, please see our Privacy Policy

Reviewer #1: **Yes:** Amjed abbas Ahmed

**********

[NOTE: If reviewer comments were submitted as an attachment file, they will be attached to this email and accessible via the submission site. Please log into your account, locate the manuscript record, and check for the action link "View Attachments". If this link does not appear, there are no attachment files.]

To ensure your figures meet our technical requirements, please review our figure guidelines: https://journals.plos.org/plosone/s/figures

You may also use PLOS’s free figure tool, NAAS, to help you prepare publication quality figures: https://journals.plos.org/plosone/s/figures#loc-tools-for-figure-preparation.

NAAS will assess whether your figures meet our technical requirements by comparing each figure against our figure specifications.

# Author comment: Author response to Decision Letter 3

**DOI:** 10.1371/journal.pone.0356146.r006

26 Jun 2026

Reviewer #1: 1. The manuscript claims that the proposed diffusion-sparse coding framework significantly improves the translation of culturally loaded words; however, the contribution of each component (sparse coding, diffusion generation, time-step-aware gating, and ontology constraints) is not sufficiently isolated. Additional ablation studies are required to quantify the individual impact of each module on translation performance.

Reply: Thank you for your valuable suggestions on the component contribution differentiation. we have added ablation experiments and used paired bootstrap tests (10,000 resamplings) to statistically quantify the contributions of each component. The results show that sparse coding and diffusion generation contribute the most significantly (p<0.001), while gating and knowledge constraint contributions are robust (p<0.005). Correlation analysis has been added to Section 3; please see the revised draft for details.

2. The cultural ontology knowledge graph contains only 186 entities and 312 relations. The manuscript does not provide sufficient evidence that this ontology is comprehensive enough to support the broad claims regarding cultural adaptability and semantic preservation across diverse news domains.

Reply: In response to the issues you raised, we supplemented the second section with the selection process for ontology nodes, demonstrated the representativeness of the ontology, and added coverage statistics to provide data to prove that the current ontology is sufficient to support the conclusions within the scope of this study’s evaluation. Finally, in the conclusion section, I pointed out that cross-domain migration requires expanding the ontology, while emphasizing the universality of the methodological framework itself.

3. The manually constructed cultural evaluation subset contains only 500 sentences covering four cultural categories. The limited size and category coverage raise concerns regarding the generalizability of the reported results to broader real-world translation scenarios.

Reply: Based on your feedback, we added a Bootstrap confidence interval analysis to Section 3 to verify that the 500 sentences possess sufficient statistical validity in a diagnostic testing scenario; and I added a cross-set consistency analysis to the analysis section of Table 8 to demonstrate that the conclusions do not depend on specific sample selection. Furthermore, the conclusions explicitly limit the generalization claim to high cultural load scenarios in English-Chinese news and point out the generality of the methodological framework and the boundaries of the evaluation conclusions.

4. The proposed Cultural Semantic Consistency Index (SCI) plays a central role in evaluating cultural fidelity, yet its validation remains limited. Additional evidence is needed to demonstrate that SCI reliably correlates with human judgments of cultural translation quality.

Reply: Based on your suggestion, we added a correlation verification experiment between SCI and human cultural fidelity assessment in the revised draft: 100 samples were stratified and randomly selected from the test set, and three independent bilingual experts were invited to conduct a blind assessment of the cultural information retention of the four types of model translations (ICC=0.876). The results showed that the Pearson correlation coefficient between SCI and CIR scores was r=0.914 (95%CI: [0.887, 0.936], p<0.001), confirming that SCI can reliably reflect human judgment on the quality of cultural translation.

5. The manuscript reports improvements in BLEU, METEOR, accuracy, recall, and SCI, but no statistical significance testing is provided. Statistical validation should be included to confirm that the observed performance gains are robust and not due to dataset-specific effects.

Reply: Based on your suggestion, we have added statistical tests and stability analysis to Section 3. We conducted statistical tests on the results of our model and the comparative models at each test index level. The results show that our model is significantly better than the three baseline models in all indices (p < 0.01).

6. The reproducibility of the study remains limited because the ontology resources, cultural evaluation subset, and associated annotation materials are not publicly available. The current “available upon request” approach restricts independent verification of the results.

Reply: Thank you for your concern about reproducibility. In Section 2.3, I have added a detailed process for ontology construction: (1) seed word extraction criteria based on dual screening of word frequency and translation error rate; (2) specific operational specifications for independent annotation and consistency judgment by three experts; (3) judgment rules for four types of relation edges; (4) training parameter settings for TransE embedding; (5) quality results verified by blind review by independent experts; so as to support the independent reproduction of ontology construction in different language pairs or domains based on unified steps and judgment criteria.

7. The experimental evaluation is restricted to English–Chinese news translation. Although multilingual applicability is discussed, no empirical evidence is provided to support the claimed transferability of the framework to other language pairs or cross-cultural translation settings.

Reply: Thank you for pointing out the errors. We have added a new section to the conclusion acknowledging that the current experiment is limited to English-Chinese news corpora, pointing out the language-independent design of the framework, and outlining specific paths for multilingual verification such as English-Japanese and English-Arabic. This limits the discussion of multilingual applicability to theoretical prospects and avoids making over-assertions on unverified scenarios.

Overall, the topic is relevant and the proposed framework is potentially valuable. However, substantial revisions are required to strengthen the experimental validation, reproducibility, statistical rigor, and evidence supporting the claimed cultural generalization capabilities.

Reply: Thank you for your affirmation of the relevance of this paper’s theme and the potential value of its framework. In response to your concerns regarding experimental verification, reproducibility, statistical rigor, and cultural generalization ability, we have supplemented the revised draft by adding ablation experiments and paired bootstrap tests to quantify the contributions of each component and verify the robustness of the results; we have also added a five-step cultural ontology construction process and verified the correlation between SCI and human evaluation, enhancing the credibility of the cultural adaptability indicators; and in the conclusion, we have limited the empirical boundary of this study to the English-Chinese news scenario and explicitly stated multilingual applicability as a theoretical prospect.

Attachment Submitted filename: Response_to_Reviewers_auresp_3.docx

# Peer review: Decision Letter 3

**DOI:** 10.1371/journal.pone.0356146.r007

**Authors:** Issa Atoum

10 Jul 2026

Dear Dr. Yu,

Thank you for submitting your manuscript to PLOS ONE. After careful consideration, we feel that it has merit but does not fully meet PLOS ONE’s publication criteria as it currently stands. Therefore, we invite you to submit a revised version of the manuscript that addresses the points raised during the review process.

While the reviewer has indicated that the authors have addressed the comments, the manuscript still contains two issues that must be resolved to comply with PLOS ONE requirements:

Incomplete equations and missing symbols: Several equations and mathematical expressions still appear to be missing symbols, possibly due to issues during PDF generation. Please ensure that the final PDF is complete and properly rendered. For example, in Table 1, the symbol in Gu appears to be missing. In addition, all equations should be explicitly cited and discussed in the text unless they represent standard formulations that are not part of the authors' original contribution.

Code availability for reproducibility: The source code is necessary to fully reproduce the results presented in the paper. While the datasets are available and Section 3.1 provides a discussion of the methodology, these alone are insufficient to ensure reproducibility. The authors should make the code available or provide a clear justification consistent with the journal’s reproducibility requirements.

Please submit your revised manuscript by Aug 24 2026 11:59PM. If you will need more time than this to complete your revisions, please reply to this message or contact the journal office at plosone@plos.org. When you're ready to submit your revision, log on to https://www.editorialmanager.com/pone/ and select the 'Submissions Needing Revision' folder to locate your manuscript file.

- A letter that responds to each point raised by the academic editor and reviewer(s). You should upload this letter as a separate file labeled 'Response to Reviewers'.
- A marked-up copy of your manuscript that highlights changes made to the original version. You should upload this as a separate file labeled 'Revised Manuscript with Track Changes'.
- An unmarked version of your revised paper without tracked changes. You should upload this as a separate file labeled 'Manuscript'.

If you would like to make changes to your financial disclosure, please include your updated statement in your cover letter. Guidelines for resubmitting your figure files are available below the reviewer comments at the end of this letter.

If applicable, we recommend that you deposit your laboratory protocols in protocols.io to enhance the reproducibility of your results. Protocols.io assigns your protocol its own identifier (DOI) so that it can be cited independently in the future. For instructions see: https://journals.plos.org/plosone/s/submission-guidelines#loc-laboratory-protocols. Additionally, PLOS ONE offers an option for publishing peer-reviewed Lab Protocol articles, which describe protocols hosted on protocols.io. Read more information on sharing protocols at https://plos.org/protocols?utm_medium=editorial-email&utm_source=authorletters&utm_campaign=protocols.

As the corresponding author, your ORCID iD is verified in the submission system and will appear in the published article. PLOS supports the use of ORCID, and we encourage all coauthors to register for an ORCID iD and use it as well. Please encourage your coauthors to verify their ORCID iD within the submission system before final acceptance, as unverified ORCID iDs will not appear in the published article. *Only*  the individual author can complete the verification step; PLOS staff *cannot*  verify ORCID iDs on behalf of authors.

We look forward to receiving your revised manuscript.

Kind regards,

Issa Atoum

Academic Editor

PLOS One

Journal Requirements:

If the reviewer comments include a recommendation to cite specific previously published works, please review and evaluate these publications to determine whether they are relevant and should be cited. There is no requirement to cite these works unless the editor has indicated otherwise.

[Note: HTML markup is below. Please do not edit.]

Reviewers' comments:

Reviewer’s Responses to Questions

**Comments to the Author**

Reviewer #1: All comments have been addressed

**********

2. Is the manuscript technically sound, and do the data support the conclusions?

Reviewer #1: Yes

**********

3. Has the statistical analysis been performed appropriately and rigorously?

Reviewer #1: Yes

**********

4. Have the authors made all data underlying the findings in their manuscript fully available?

The PLOS Data policy

Reviewer #1: Yes

**********

5. Is the manuscript presented in an intelligible fashion and written in standard English?

Reviewer #1: Yes

**********

Reviewer #1: Thank you for your careful and comprehensive revision of the manuscript. I appreciate the considerable effort invested in addressing the reviewers' comments throughout the revision process. The current version has been substantially strengthened in both technical rigor and presentation.The authors have adequately addressed the major concerns raised in the previous review rounds by including comprehensive ablation studies, statistical significance analyses, validation of the proposed Cultural Semantic Consistency Index against human judgments, clearer descriptions of the ontology construction process, improved discussion of the study’s limitations, and a more balanced interpretation of the framework’s applicability. The manuscript is now significantly more transparent, reproducible, and scientifically convincing. The proposed integration of sparse coding and diffusion-based generation provides a novel and technically sound contribution to culturally aware machine translation, and the experimental evaluation supports the effectiveness of the proposed approach within the stated scope.

Overall, I believe the manuscript has reached a publishable standard. Only minor editorial proofreading may be performed during production if necessary.

**********

what does this mean?). If published, this will include your full peer review and any attached files.

If you choose “no”, your identity will remain anonymous but your review may still be made public.

**Do you want your identity to be public for this peer review?**  For information about this choice, including consent withdrawal, please see our Privacy Policy

Reviewer #1: **Yes:** Amjed Abbas Ahmed

**********

[NOTE: If reviewer comments were submitted as an attachment file, they will be attached to this email and accessible via the submission site. Please log into your account, locate the manuscript record, and check for the action link "View Attachments". If this link does not appear, there are no attachment files.]

To ensure your figures meet our technical requirements, please review our figure guidelines: https://journals.plos.org/plosone/s/figures

You may also use PLOS’s free figure tool, NAAS, to help you prepare publication quality figures: https://journals.plos.org/plosone/s/figures#loc-tools-for-figure-preparation.

NAAS will assess whether your figures meet our technical requirements by comparing each figure against our figure specifications.

# Author comment: Author response to Decision Letter 4

**DOI:** 10.1371/journal.pone.0356146.r008

14 Jul 2026

While the reviewer has indicated that the authors have addressed the comments, the manuscript still contains two issues that must be resolved to comply with PLOS ONE requirements:

Incomplete equations and missing symbols: Several equations and mathematical expressions still appear to be missing symbols, possibly due to issues during PDF generation. Please ensure that the final PDF is complete and properly rendered. For example, in Table 1, the symbol in Gu appears to be missing. In addition, all equations should be explicitly cited and discussed in the text unless they represent standard formulations that are not part of the authors' original contribution.

Answer: According to the formula rendering and citation issues you pointed out, we have corrected the formula symbols in Table 1, added references to all formulas one by one, and clarified the specific role of key formulas in sparse coding, dynamic gating and cultural constraints, improving the readability of the article.

Code availability for reproducibility: The source code is necessary to fully reproduce the results presented in the paper. While the datasets are available and Section 3.1 provides a discussion of the methodology, these alone are insufficient to ensure reproducibility. The authors should make the code available or provide a clear justification consistent with the journal’s reproducibility requirements.

Answer: Thank you for pointing out the importance of code usability. We highly value the reproducibility of our research and, in accordance with journal policy, have uploaded the source code as supplementary material.

Attachment Submitted filename: Response_to_Reviewers_auresp_4.docx

# Editor report: Decision Letter 4

**DOI:** 10.1371/journal.pone.0356146.r009

**Authors:** Issa Atoum

16 Jul 2026

Dear Dr. Yu,

Thank you for submitting your manuscript to PLOS ONE. After careful consideration, we feel that it has merit but does not fully meet PLOS ONE’s publication criteria as it currently stands. Therefore, we invite you to submit a revised version of the manuscript that addresses the points raised during the review process.

==============================

Please submit your revised manuscript by Aug 30 2026 11:59PM. If you will need more time than this to complete your revisions, please reply to this message or contact the journal office at plosone@plos.org. When you're ready to submit your revision, log on to https://www.editorialmanager.com/pone/ and select the 'Submissions Needing Revision' folder to locate your manuscript file.

- A letter that responds to each point raised by the academic editor and reviewer(s). You should upload this letter as a separate file labeled 'Response to Reviewers'.
- A marked-up copy of your manuscript that highlights changes made to the original version. You should upload this as a separate file labeled 'Revised Manuscript with Track Changes'.
- An unmarked version of your revised paper without tracked changes. You should upload this as a separate file labeled 'Manuscript'.

If you would like to make changes to your financial disclosure, please include your updated statement in your cover letter. Guidelines for resubmitting your figure files are available below the reviewer comments at the end of this letter.

If applicable, we recommend that you deposit your laboratory protocols in protocols.io to enhance the reproducibility of your results. Protocols.io assigns your protocol its own identifier (DOI) so that it can be cited independently in the future. For instructions see: https://journals.plos.org/plosone/s/submission-guidelines#loc-laboratory-protocols. Additionally, PLOS ONE offers an option for publishing peer-reviewed Lab Protocol articles, which describe protocols hosted on protocols.io. Read more information on sharing protocols at https://plos.org/protocols?utm_medium=editorial-email&utm_source=authorletters&utm_campaign=protocols.

As the corresponding author, your ORCID iD is verified in the submission system and will appear in the published article. PLOS supports the use of ORCID, and we encourage all coauthors to register for an ORCID iD and use it as well. Please encourage your coauthors to verify their ORCID iD within the submission system before final acceptance, as unverified ORCID iDs will not appear in the published article. *Only*  the individual author can complete the verification step; PLOS staff *cannot*  verify ORCID iDs on behalf of authors.

We look forward to receiving your revised manuscript.

Kind regards,

Issa Atoum

Academic Editor

PLOS One

Journal Requirements:

If the reviewer comments include a recommendation to cite specific previously published works, please review and evaluate these publications to determine whether they are relevant and should be cited. There is no requirement to cite these works unless the editor has indicated otherwise.

Please review your reference list to ensure that it is complete and correct. If you have cited papers that have been retracted, please include the rationale for doing so in the manuscript text, or remove these references and replace them with relevant current references. Any changes to the reference list should be mentioned in the rebuttal letter that accompanies your revised manuscript. If you need to cite a retracted article, indicate the article’s retracted status in the References list and also include a citation and full reference for the retraction notice.

[Note: HTML markup is below. Please do not edit.]

[NOTE: If reviewer comments were submitted as an attachment file, they will be attached to this email and accessible via the submission site. Please log into your account, locate the manuscript record, and check for the action link "View Attachments". If this link does not appear, there are no attachment files.]

To ensure your figures meet our technical requirements, please review our figure guidelines: https://journals.plos.org/plosone/s/figures

You may also use PLOS’s free figure tool, NAAS, to help you prepare publication quality figures: https://journals.plos.org/plosone/s/figures#loc-tools-for-figure-preparation.

NAAS will assess whether your figures meet our technical requirements by comparing each figure against our figure specifications.

This is the third time that equation symbols have not rendered correctly. Please check the generated file before approval. The issue affects not only Table 1 but multiple equations throughout the manuscript. For example, in Table 1, the formula displays <code>ℝ ， gu∈[0,1]</code> with an empty square (□) before **g** , indicating a missing symbol. Please review all equations and mathematical symbols carefully.

# Author comment: Author response to Decision Letter 5

**DOI:** 10.1371/journal.pone.0356146.r010

21 Jul 2026

PONE-D-26-07189R4

Combining a diffusion model with a sparse coding strategy to improve the machine translation accuracy of culturally loaded words in English news texts

PLOS One

Dear Dr. Yu,

Thank you for submitting your manuscript to PLOS ONE. After careful consideration, we feel that it has merit but does not fully meet PLOS ONE’s publication criteria as it currently stands. Therefore, we invite you to submit a revised version of the manuscript that addresses the points raised during the review process.

Answer: We sincerely thank you and the reviewers for your positive feedback and valuable comments. We have revised the manuscript according to each reviewer’s comment, paying particular attention to the display issues of formula symbols, correcting rendering errors in mathematical symbols and table variables, and generating a new PDF for verification. The revised manuscript has been uploaded in its entirety, and we kindly request your review. We hope the manuscript will meet PLOS ONE’s publication standards. Thank you again for your support and guidance!

==============================

This is the third time that equation symbols have not rendered correctly. Please check the generated file before approval. The issue affects not only Table 1 but multiple equations throughout the manuscript. For example, in Table 1, the formula displays ℝ ， □gu∈[0,1] with an empty square (□) before g, indicating a missing symbol. Please review all equations and mathematical symbols carefully.

Answer: We sincerely apologize for the issue you pointed out regarding the display of formula symbols. We have rectified the mathematical symbols with rendering errors: we specifically corrected "□gu" in Table 1 and checked all inline and cross-line formulas one by one to ensure that the symbols were rendered correctly. The revised version has been recompiled and a new PDF has been generated for verification. Thank you for your meticulous correction!

==============================

Please submit your revised manuscript by Aug 30 2026 11:59PM. If you will need more time than this to complete your revisions, please reply to this message or contact the journal office at plosone@plos.org. When you're ready to submit your revision, log on to https://www.editorialmanager.com/pone/ and select the 'Submissions Needing Revision' folder to locate your manuscript file.

Please include the following items when submitting your revised manuscript:

�A letter that responds to each point raised by the academic editor and reviewer(s). You should upload this letter as a separate file labeled 'Response to Reviewers'.

�A marked-up copy of your manuscript that highlights changes made to the original version. You should upload this as a separate file labeled 'Revised Manuscript with Track Changes'.

�An unmarked version of your revised paper without tracked changes. You should upload this as a separate file labeled 'Manuscript'.

If you would like to make changes to your financial disclosure, please include your updated statement in your cover letter. Guidelines for resubmitting your figure files are available below the reviewer comments at the end of this letter.

Answer: Thank you for providing the detailed revision submission guidelines. We have prepared all materials as required: 1) A separate response letter to each reviewer’s comment, titled "Response to Reviewers"; 2) An uploaded revised manuscript with tracked changes, named "Revised Manuscript with Track Changes"; 3) An uploaded final manuscript without any markings, named "Manuscript". All figures and tables have been resubmitted according to the guidelines. These revisions address all reviewer comments; your review is appreciated.

If applicable, we recommend that you deposit your laboratory protocols in protocols.io to enhance the reproducibility of your results. Protocols.io assigns your protocol its own identifier (DOI) so that it can be cited independently in the future. For instructions see: https://journals.plos.org/plosone/s/submission-guidelines#loc-laboratory-protocols. Additionally, PLOS ONE offers an option for publishing peer-reviewed Lab Protocol articles, which describe protocols hosted on protocols.io. Read more information on sharing protocols at https://plos.org/protocols?utm_medium=editorial-email&utm_source=authorletters&utm_campaign=protocols.

Answer: Thank you for your suggestion regarding sharing the experimental protocol. We fully understand the protocols.io platform and its value in improving research reproducibility. Considering that this research primarily involves model architecture and algorithm implementation, and does not involve traditional wet testing procedures, we have not yet established a dedicated experimental protocol on protocols.io. However, we have described the model parameters, training process, and code implementation details in detail in the manuscript and methods sections, and will provide access to the core code in the data availability statement to ensure the reproducibility of the research results to the greatest extent possible. We are happy to cooperate if there are any further requests.

As the corresponding author, your ORCID iD is verified in the submission system and will appear in the published article. PLOS supports the use of ORCID, and we encourage all coauthors to register for an ORCID iD and use it as well. Please encourage your coauthors to verify their ORCID iD within the submission system before final acceptance, as unverified ORCID iDs will not appear in the published article. Only the individual author can complete the verification step; PLOS staff cannot verify ORCID iDs on behalf of authors.

Answer: Thank you for your reminder regarding ORCID verification. As the corresponding author, I verified my own ORCID in the system upon submission. I also notified all co-authors via email, urging them to log in to the submission system and verify their respective ORCIDs as soon as possible before final acceptance of the article, ensuring that all named authors' ORCIDs are displayed correctly upon publication. I will continue to follow up on this matter and will reconfirm the verification status of all authors before acceptance.

We look forward to receiving your revised manuscript.

Kind regards,

Issa Atoum

Academic Editor

PLOS One

Journal Requirements:

If the reviewer comments include a recommendation to cite specific previously published works, please review and evaluate these publications to determine whether they are relevant and should be cited. There is no requirement to cite these works unless the editor has indicated otherwise.

Please review your reference list to ensure that it is complete and correct. If you have cited papers that have been retracted, please include the rationale for doing so in the manuscript text, or remove these references and replace them with relevant current references. Any changes to the reference list should be mentioned in the rebuttal letter that accompanies your revised manuscript. If you need to cite a retracted article, indicate the article’s retracted status in the References list and also include a citation and full reference for the retraction notice.

Answer: Thank you for your reminder regarding the references. We have reviewed the full-text reference list and confirmed that all cited references have not been retracted and are formatted correctly and completely. Any additions or deletions to the references were clearly indicated in the reply email. We will continue to monitor the status of the references to ensure the reliability of the cited content. Thank you for your meticulous review!

[Note: HTML markup is below. Please do not edit.]

[NOTE: If reviewer comments were submitted as an attachment file, they will be attached to this email and accessible via the submission site. Please log into your account, locate the manuscript record, and check for the action link "View Attachments". If this link does not appear, there are no attachment files.]

To ensure your figures meet our technical requirements, please review our figure guidelines: https://journals.plos.org/plosone/s/figures

You may also use PLOS’s free figure tool, NAAS, to help you prepare publication quality figures: https://journals.plos.org/plosone/s/figures#loc-tools-for-figure-preparation.

NAAS will assess whether your figures meet our technical requirements by comparing each figure against our figure specifications.

Answer: Thank you for providing the figures guide and the NAAS tool link. We have carefully reviewed the relevant technical requirements to ensure that the documents meet the specifications regarding format, resolution, size, and file size. The revised manuscript includes figures that meet publication quality requirements. If you encounter any technical issues, we will contact the journal support promptly. Thank you for your detailed guidance!

Copilot said:

This is the third time that equation symbols have not rendered correctly. Please check the generated file before approval. The issue affects not only Table 1 but multiple equations throughout the manuscript. For example, in Table 1, the formula displays ℝ ， gu∈[0,1] with an empty square (□) before g, indicating a missing symbol. Please review all equations and mathematical symbols carefully.

Answer: We sincerely apologize for the issue you pointed out regarding the display of formula symbols. We have rectified the mathematical symbols with rendering errors: we specifically corrected "□gu" in Table 1 and checked all inline and cross-line formulas one by one to ensure that the symbols were rendered correctly. The revised version has been recompiled and a new PDF has been generated for verification. Thank you for your meticulous correction!

Attachment Submitted filename: replyy letter.docx

# Editor report: Decision Letter 5

**DOI:** 10.1371/journal.pone.0356146.r011

**Authors:** Issa Atoum

23 Jul 2026

Dear Dr. Yu,

Thank you for submitting your manuscript to PLOS ONE. After careful consideration, we feel that it has merit but does not fully meet PLOS ONE’s publication criteria as it currently stands. Therefore, we invite you to submit a revised version of the manuscript that addresses the points raised during the review process.

==============================

Unfortunately, the equations are still not displayed correctly. Please try to use LaTeX. Moreover, the provided code is not fully reproducible because it is isolated from the datasets and corresponding outputs. If sharing the datasets is not possible, please provide executable notebooks that reproduce the reported results and demonstrate the complete analysis workflow.

==============================

Please submit your revised manuscript by Sep 06 2026 11:59PM. If you will need more time than this to complete your revisions, please reply to this message or contact the journal office at plosone@plos.org. When you're ready to submit your revision, log on to https://www.editorialmanager.com/pone/ and select the 'Submissions Needing Revision' folder to locate your manuscript file.

- A letter that responds to each point raised by the academic editor and reviewer(s). You should upload this letter as a separate file labeled 'Response to Reviewers'.
- A marked-up copy of your manuscript that highlights changes made to the original version. You should upload this as a separate file labeled 'Revised Manuscript with Track Changes'.
- An unmarked version of your revised paper without tracked changes. You should upload this as a separate file labeled 'Manuscript'.

If you would like to make changes to your financial disclosure, please include your updated statement in your cover letter. Guidelines for resubmitting your figure files are available below the reviewer comments at the end of this letter.

If applicable, we recommend that you deposit your laboratory protocols in protocols.io to enhance the reproducibility of your results. Protocols.io assigns your protocol its own identifier (DOI) so that it can be cited independently in the future. For instructions see: https://journals.plos.org/plosone/s/submission-guidelines#loc-laboratory-protocols. Additionally, PLOS ONE offers an option for publishing peer-reviewed Lab Protocol articles, which describe protocols hosted on protocols.io. Read more information on sharing protocols at https://plos.org/protocols?utm_medium=editorial-email&utm_source=authorletters&utm_campaign=protocols.

As the corresponding author, your ORCID iD is verified in the submission system and will appear in the published article. PLOS supports the use of ORCID, and we encourage all coauthors to register for an ORCID iD and use it as well. Please encourage your coauthors to verify their ORCID iD within the submission system before final acceptance, as unverified ORCID iDs will not appear in the published article. *Only*  the individual author can complete the verification step; PLOS staff *cannot*  verify ORCID iDs on behalf of authors.

We look forward to receiving your revised manuscript.

Kind regards,

Issa Atoum

Academic Editor

PLOS One

Journal Requirements:

If the reviewer comments include a recommendation to cite specific previously published works, please review and evaluate these publications to determine whether they are relevant and should be cited. There is no requirement to cite these works unless the editor has indicated otherwise.

[Note: HTML markup is below. Please do not edit.]

[NOTE: If reviewer comments were submitted as an attachment file, they will be attached to this email and accessible via the submission site. Please log into your account, locate the manuscript record, and check for the action link "View Attachments". If this link does not appear, there are no attachment files.]

To ensure your figures meet our technical requirements, please review our figure guidelines: https://journals.plos.org/plosone/s/figures

You may also use PLOS’s free figure tool, NAAS, to help you prepare publication quality figures: https://journals.plos.org/plosone/s/figures#loc-tools-for-figure-preparation.

NAAS will assess whether your figures meet our technical requirements by comparing each figure against our figure specifications.

# Author comment: Author response to Decision Letter 6

**DOI:** 10.1371/journal.pone.0356146.r012

28 Jul 2026

ear Dr. Yu,

Thank you for submitting your manuscript to PLOS ONE. After careful consideration, we feel that it has merit but does not fully meet PLOS ONE’s publication criteria as it currently stands. Therefore, we invite you to submit a revised version of the manuscript that addresses the points raised during the review process.

==============================

Unfortunately, the equations are still not displayed correctly. Please try to use LaTeX. Moreover, the provided code is not fully reproducible because it is isolated from the datasets and corresponding outputs. If sharing the datasets is not possible, please provide executable notebooks that reproduce the reported results and demonstrate the complete analysis workflow.

==============================

Answer: Thank you for your valuable feedback. Regarding the formula display issue, after review, we found that some formula symbols in the original manuscript were mistakenly converted to plain text during the format conversion process, resulting in rendering distortion. Therefore, we have rewritten all formulas using standard LaTeX syntax and output files for verification. Error-free compiled PDF files are included as supplementary files in the folder. Regarding code and data reproduction, the WMT18 and TED corpora used in this study are publicly available resources. To ensure reproducibility, we have provided complete executable files in the supplementary materials, including preprocessing, model training, and evaluation procedures, and fixed random seeds and hyperparameters. Approximate test sets can be reconstructed from the publicly available corpora according to the instructions.

Please submit your revised manuscript by Sep 06 2026 11:59PM. If you will need more time than this to complete your revisions, please reply to this message or contact the journal office at plosone@plos.org. When you're ready to submit your revision, log on to https://www.editorialmanager.com/pone/ and select the 'Submissions Needing Revision' folder to locate your manuscript file.

Answer: Thank you for your letter and deadline reminder. We confirm that we will submit the revised manuscript through the system by 23:59 on September 6, 2026. Regarding the reviewers' comments, we have corrected all formulas to LaTeX format and provided detailed data construction instructions to ensure reproducibility. We will contact the editorial office promptly if necessary. Thank you again for your support!

Please include the following items when submitting your revised manuscript:

�A letter that responds to each point raised by the academic editor and reviewer(s). You should upload this letter as a separate file labeled 'Response to Reviewers'.

�A marked-up copy of your manuscript that highlights changes made to the original version. You should upload this as a separate file labeled 'Revised Manuscript with Track Changes'.

�An unmarked version of your revised paper without tracked changes. You should upload this as a separate file labeled 'Manuscript'.

If you would like to make changes to your financial disclosure, please include your updated statement in your cover letter. Guidelines for resubmitting your figure files are available below the reviewer comments at the end of this letter.

Answer: Thank you for your detailed instructions regarding the submission of the revised manuscript. We have prepared the following as required: a letter responding to each of the reviewers' comments, a revised manuscript with revision marks, and a final, unmarked manuscript. All documents were uploaded to the system before the deadline. Regarding the supplementary instructions on resubmitting financial disclosures and charts, we will carefully review each requirement to ensure that the materials are complete and compliant. Thank you again for your guidance!

If applicable, we recommend that you deposit your laboratory protocols in protocols.io to enhance the reproducibility of your results. Protocols.io assigns your protocol its own identifier (DOI) so that it can be cited independently in the future. For instructions see: https://journals.plos.org/plosone/s/submission-guidelines#loc-laboratory-protocols. Additionally, PLOS ONE offers an option for publishing peer-reviewed Lab Protocol articles, which describe protocols hosted on protocols.io. Read more information on sharing protocols at https://plos.org/protocols?utm_medium=editorial-email&utm_source=authorletters&utm_campaign=protocols.

Answer: Thank you for your suggestion regarding sharing the experimental protocol. We fully understand the protocols.io platform and its value in improving research reproducibility. Considering that this research primarily involves model architecture and algorithm implementation, and does not involve traditional wet testing procedures, we have not yet established a dedicated experimental protocol on protocols.io. However, we have described the model parameters, training process, and code implementation details in detail in the manuscript and methods sections, and will provide access to the core code in the data availability statement to ensure the reproducibility of the research results to the greatest extent possible. We are happy to cooperate if there are any further requests.

We look forward to receiving your revised manuscript.

Kind regards,

Issa Atoum

Academic Editor

PLOS One

Journal Requirements:

If the reviewer comments include a recommendation to cite specific previously published works, please review and evaluate these publications to determine whether they are relevant and should be cited. There is no requirement to cite these works unless the editor has indicated otherwise.

Answer: Thank you for your reminder regarding the references. We have reviewed the full-text reference list and confirmed that all cited references have not been retracted and are formatted correctly and completely. Any additions or deletions to the references were clearly indicated in the reply email. We will continue to monitor the status of the references to ensure the reliability of the cited content. Thank you for your meticulous review!

[Note: HTML markup is below. Please do not edit.]

[NOTE: If reviewer comments were submitted as an attachment file, they will be attached to this email and accessible via the submission site. Please log into your account, locate the manuscript record, and check for the action link "View Attachments". If this link does not appear, there are no attachment files.]

To ensure your figures meet our technical requirements, please review our figure guidelines: https://journals.plos.org/plosone/s/figures

You may also use PLOS’s free figure tool, NAAS, to help you prepare publication quality figures: https://journals.plos.org/plosone/s/figures#loc-tools-for-figure-preparation.

NAAS will assess whether your figures meet our technical requirements by comparing each figure against our figure specifications.

Answer: Thank you for providing the figures guide and the NAAS tool link. We have carefully reviewed the relevant technical requirements to ensure that the documents meet the specifications regarding format, resolution, size, and file size. The revised manuscript includes figures that meet publication quality requirements. If you encounter any technical issues, we will contact the journal support promptly. Thank you for your detailed guidance!

Attachment Submitted filename: Response_to_Reviewers_auresp_6.docx

# Editor report: Decision Letter 6

**DOI:** 10.1371/journal.pone.0356146.r013

**Authors:** Issa Atoum

31 Jul 2026

Combining a diffusion model with a sparse coding strategy to improve the machine translation accuracy of culturally loaded words in English news texts

PONE-D-26-07189R6

Dear Dr. Yu,

We’re pleased to inform you that your manuscript has been judged scientifically suitable for publication and will be formally accepted for publication once it meets all outstanding technical requirements.

Consider only adding "element of / belongs to" in table 1 (seventh line).

Within one week, you’ll receive an e-mail detailing the required amendments. When these have been addressed, you’ll receive a formal acceptance letter and your manuscript will be scheduled for publication.

An invoice will be generated when your article is formally accepted. Please note, if your institution has a publishing partnership with PLOS and your article meets the relevant criteria, all or part of your publication costs will be covered. Please make sure your user information is up-to-date by logging into Editorial Manager at Editorial Manager® and clicking the ‘Update My Information' link at the top of the page. For questions related to billing, please contact billing support.

If your institution or institutions have a press office, please notify them about your upcoming paper to help maximize its impact. If they’ll be preparing press materials, please inform our press team as soon as possible -- no later than 48 hours after receiving the formal acceptance. Your manuscript will remain under strict press embargo until 2 pm Eastern Time on the date of publication. For more information, please contact onepress@plos.org.

Kind regards,

Issa Atoum

Academic Editor

PLOS One

Additional Editor Comments (optional):

Reviewers' comments:

# Editor report: Acceptance letter

**DOI:** 10.1371/journal.pone.0356146.r014

**Authors:** Issa Atoum

PONE-D-26-07189R6

PLOS One

Dear Dr. Yu,

I'm pleased to inform you that your manuscript has been deemed suitable for publication in PLOS One. Congratulations! Your manuscript is now being handed over to our production team.

At this stage, our production department will prepare your paper for publication. This includes ensuring the following:

* All references, tables, and figures are properly cited

* All relevant supporting information is included in the manuscript submission,

* There are no issues that prevent the paper from being properly typeset

You will receive further instructions from the production team, including instructions on how to review your proof when it is ready. Please keep in mind that we are working through a large volume of accepted articles, so please give us a few days to review your paper and let you know the next and final steps.

Lastly, if your institution or institutions have a press office, please let them know about your upcoming paper now to help maximize its impact. If they'll be preparing press materials, please inform our press team within the next 48 hours. Your manuscript will remain under strict press embargo until 2 pm Eastern Time on the date of publication. For more information, please contact onepress@plos.org.

You will receive an invoice from PLOS for your publication fee after your manuscript has reached the completed accept phase. If you receive an email requesting payment before acceptance or for any other service, this may be a phishing scheme. Learn how to identify phishing emails and protect your accounts at https://explore.plos.org/phishing.

If we can help with anything else, please email us at customercare@plos.org.

Thank you for submitting your work to PLOS One and supporting open access.

Kind regards,

PLOS ONE Editorial Office Staff

on behalf of

Dr. Issa Atoum

Academic Editor

PLOS One
